# Supplementary material for: Eicosapentaenoic acid induces macrophage Mox polarization to prevent diabetic cardiomyopathy
Source: EMBO Rep. 2024 Oct 31;25(12):5507–36. doi: 10.1038/s44319-024-00271-x (PMC11624267; doi:10.1038/s44319-024-00271-x)
Supplement: Supplementary file 1 — Appendix [file 44319_2024_271_MOESM1_ESM.pdf]

Table of Contents

1. Appendix Tables 2

Appendix Table S1 2

Appendix Table S2 2

Appendix Table S3 3

2. Appendix Figures 4

Appendix Figure S1 4

Appendix Figure S2 5

Appendix Figure S3 6

Appendix Figure S4 7

Appendix Figure S5 7

Appendix Figure S6 8

Appendix Figure S7 9

Appendix Figure S8 10

3. Appendix Figure Legends 10

1. Appendix Tables

Appendix Table S1. The clinical information of subjects

	Ctrl	ox-PAPC	DMSO	EPA
Age (year)	55.33±3.94	54.83±4.47	49.83±3.2	59.33±3.57
Male	3	3	3	3
Female	3	3	3	3
HbA_{1c} (%)	9.18±0.66	9.6±0.69	9.52±0.74	9.6±0.62
FPG (mmol/l)	11.54±1.55	11.69±1.19	10.36±0.5	12.86±1.72
BMI (kg/m^2)	23.6±1.24	25.67±0.97	24.9±1.4	23.6±0.6
Duration of T2DM (year)	9.67±2.93	11.67±3.53	7.67±2.62	12.67±2.76

BMI: Body mass index; FPG: Fasting plasma glucose; HbA_{1c}: Glycosylated hemoglobin.

Appendix Table S2. Composition of the experimental diets

**Ingredient (g/kg)**	**Normal diet**	**HFD diet**	**HFD+EPA diet**
Soybean	70	70	50
EPA	-	-	20
Lard	-	230	230
β-cornstarch	397.49	110.62	110.62
α-(dextrinized) cornstarch	132	36.87	36.87
Casein	200	250	250
Sucrose	100	200	200
AIN-93 mineral mixture	35	35	35
AIN-93 vitamin mixture	10	10	10
L-Cystine	3	3.75	3.75
Choline bitatrate	2.5	2.5	2.5
Cellulose	50	50	50
Tert-Butylhydroquinone	0.01	0.06	0.06
Cholesterol	-	1.2	1.2
Protein	203	253.75	253.75
Carbohydrates	629.49	347.49	347.49
Fat	70	300	300

Appendix Table S3. Primer sequences

**Gene (mouse)**	**Forward Primer Sequence**	**Reverse Primer Sequence**
Arg1	ACATTGGCTTGCGAGACGTA	ATCACCTTGCCAATCCCCAG
Ccl2	CAGGTCACTTTGAGTCCCCT	TGGTATACATGGAGGGGCTG
Gapdh	TGGTGAAGGTCGGTGTGAAC	GCTCCTGGAAGATGGTGATGG
Hmox1	AGGGTCAGGTGTCCAGAGAA	CTTCCAGGGCCGTGTAGATA
Ifn-γ	ATCTGGAGGAACTGGCAAAA	TTCAAGACTTCAAAGAGTCTGAGG
Il-1β	AAATGCCACCTTTTGACAGTGATG	GCAGCCCTTCATCTTTTGGG
Il-6	CCAGGTAGCTATGGTACTCCAGAA	GCTACCAAACTGGATATAATCAGGA
Il-10	GGCAGAGAACCATGGCCCAGAA	AATCGATGACAGCGCCTCAGCC
Nos2	CAGCTGGGCTGTACAAACCTT	CATTGGAAGTGAAGCGTTTCG
Nox4	CTTGGTGAATGCCCTCAACT	TTCTGGGATCCTCATTCTGG
Srxn1	GTGCACAACGTACCAATCG	GCCCCCAAAGGAATAGTAGTAG
Tgf-β1	AGGAAGGACCTGGGTTGGAAG	CGTCTCGACCCACGTAGTAGACG
Tnf-α	ATCTACCTGGGAGGCGTCTT	GAGTGGCACAAGGAACTGGT
TrxR1	CCCACTTGCCCCAACTGTT	GGGAGTGTCTTGGAGGGAC
Vegf-α	CTGCCGTCCGATTGAGACC	CCCCTCCTTGTACCACTGTC

2. Appendix Figures

Appendix Figure S1

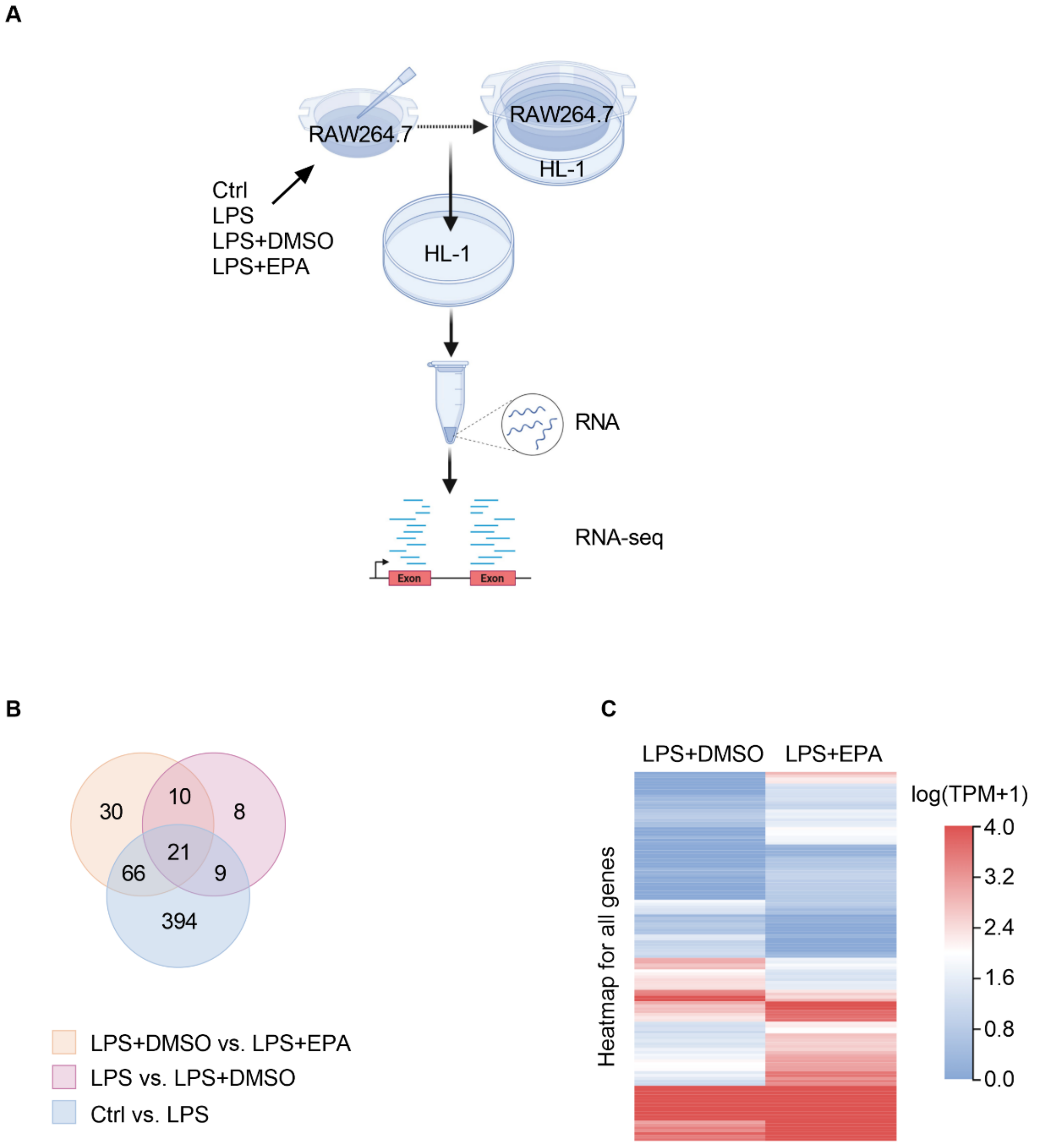

Appendix Figure S2

A

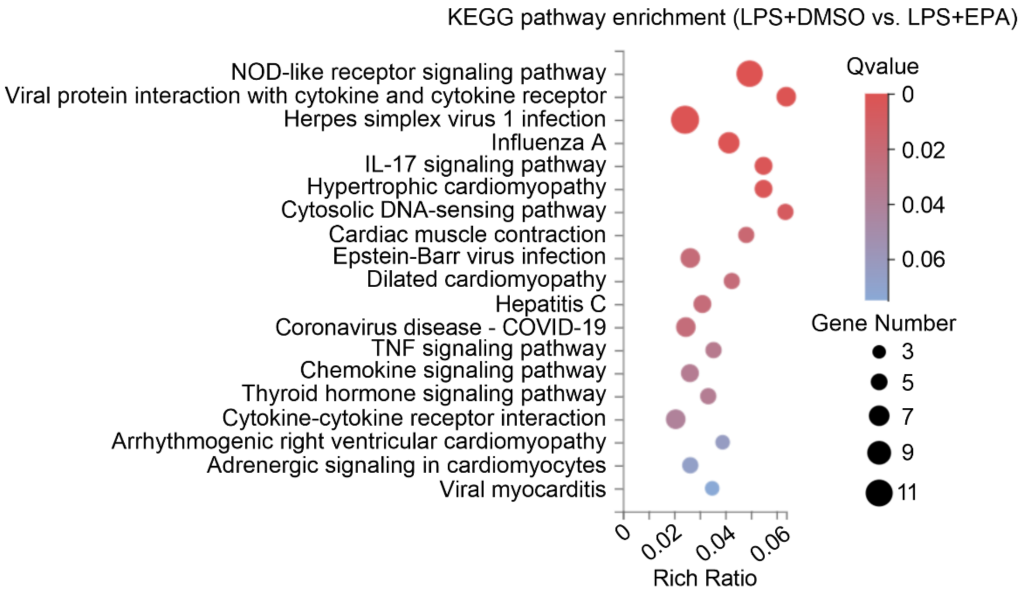

B

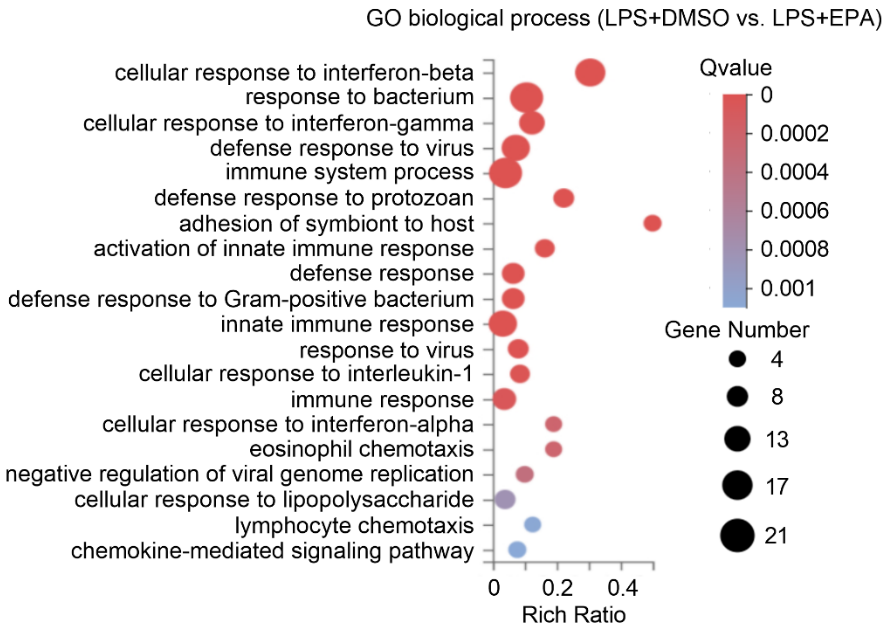

Appendix Figure S3

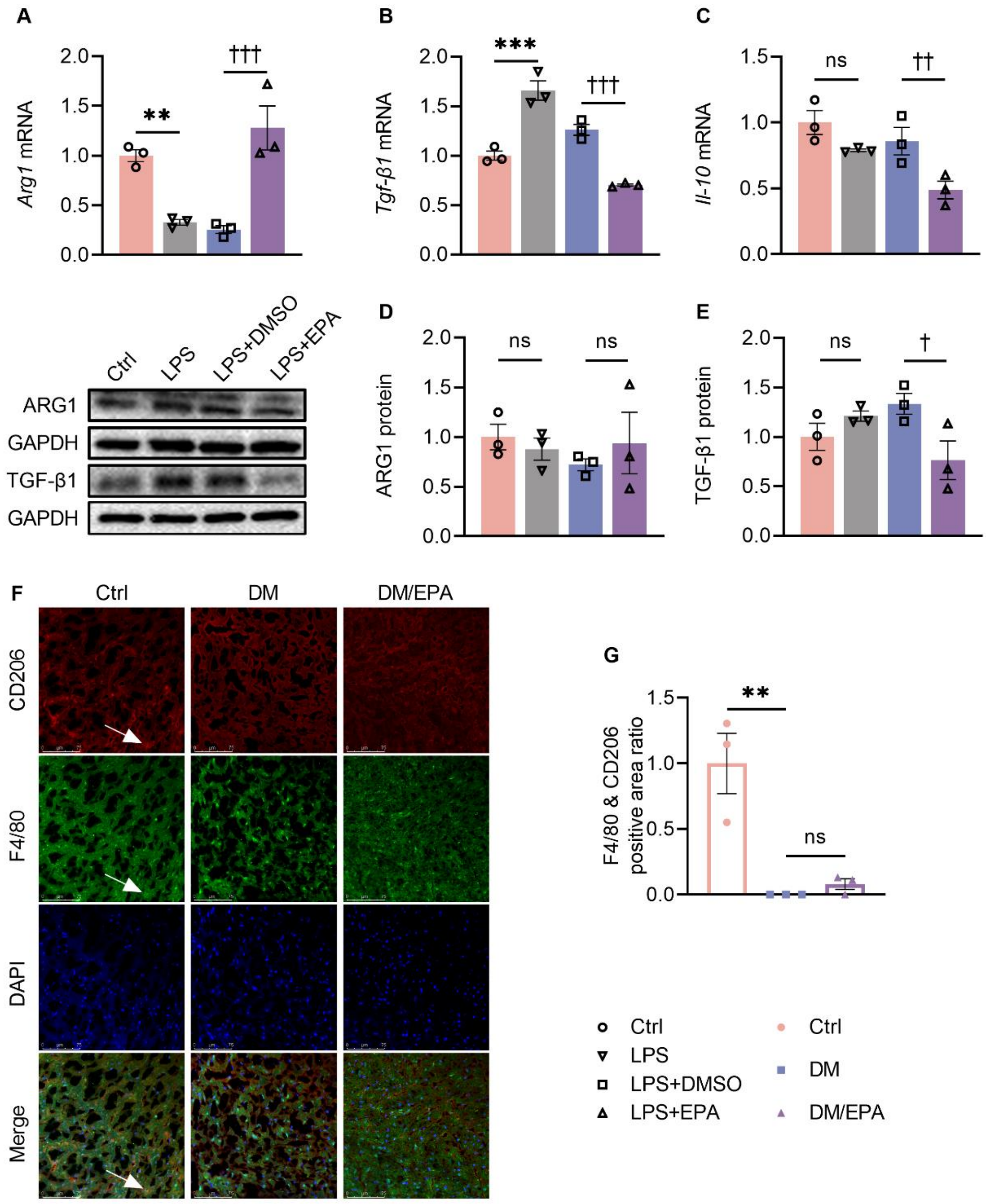

Appendix Figure S4

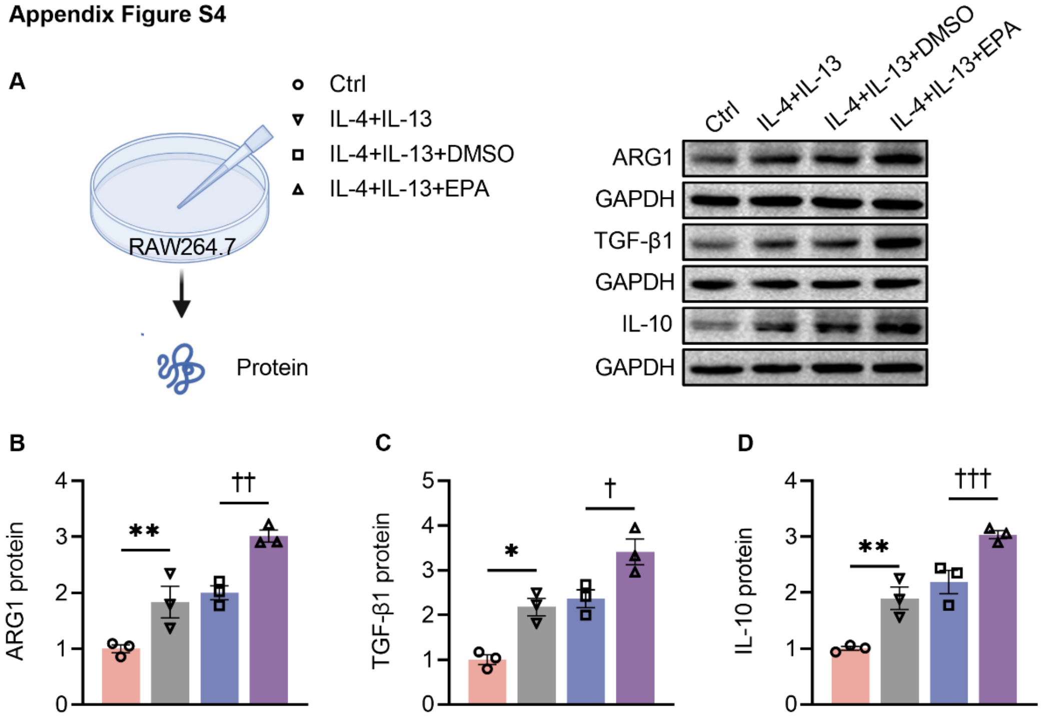

Appendix Figure S5

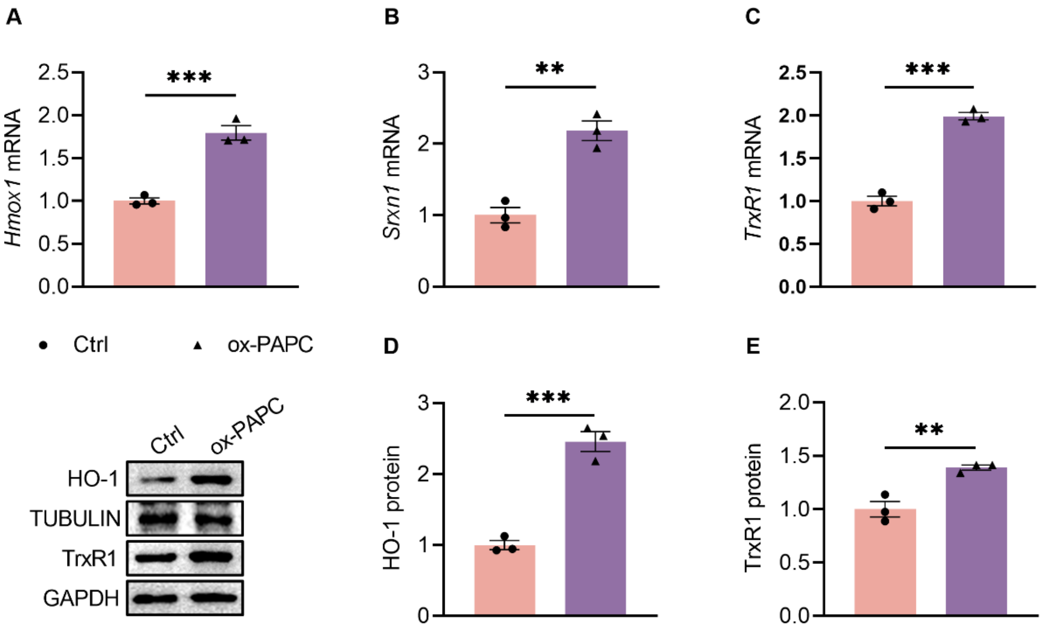

Appendix Figure S6

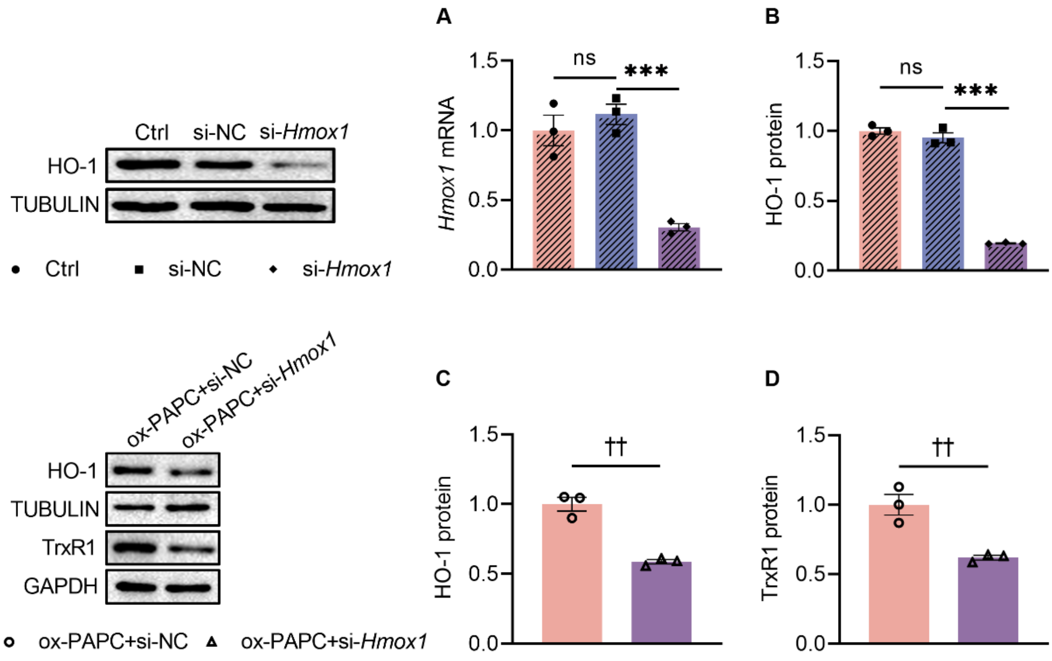

Appendix Figure S7

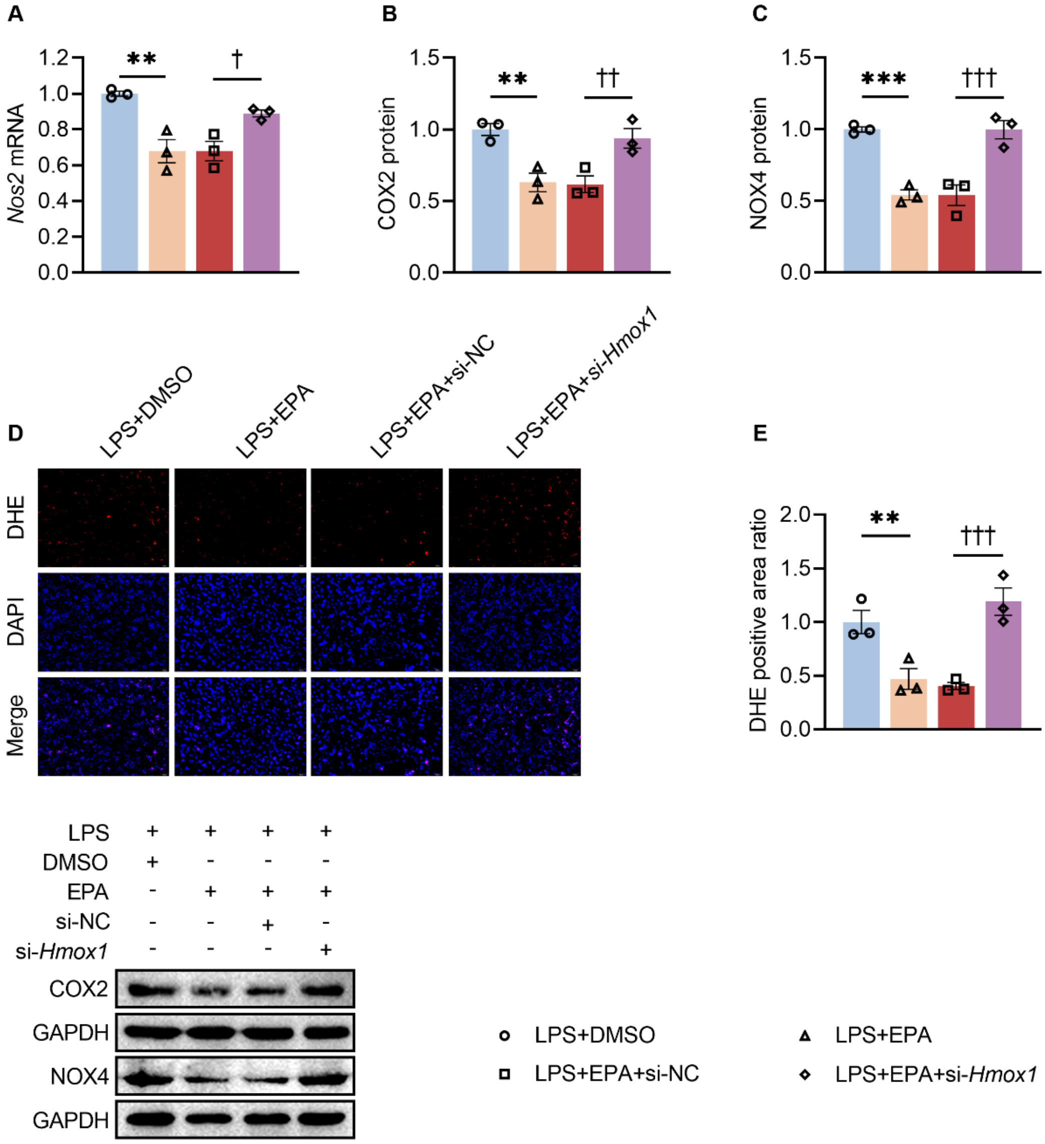

Appendix Figure S8

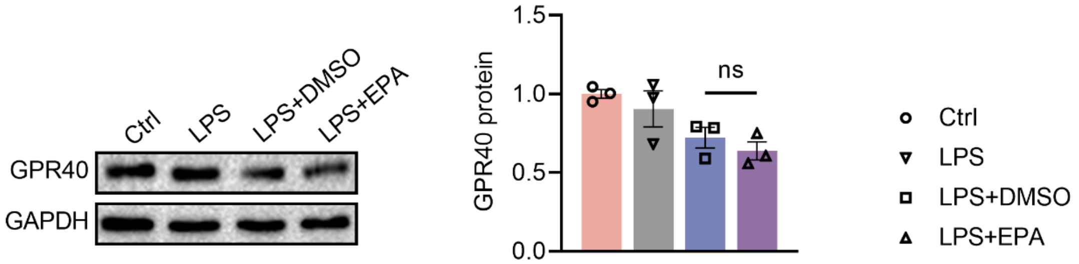

3. Appendix Figure Legends

Appendix Figure S1. EPA affected the mRNA expression profile of HL-1 cells co-cultured with M1 polarized macrophages. RNA-seq was performed for analysis of EPA's effect on global mRNA expression profile of HL-1 cells co-cultured with LPS-treated RAW264.7 cells. (**A**) Schematic representation of the RNA-seq protocol. (**B**) Venn diagram showing comparisons of altered gene numbers among the groups. (**C**) Heat map comparison of the global mRNA expression profiles in LPS+DMSO and LPS+EPA groups. Abbreviations: EPA, eicosapentaenoic acid; LPS, lipopolysaccharides; RNA-seq, RNA-sequencing. Groups: Ctrl, control; LPS, HL-1 cells co-cultured with LPS-stimulated RAW264.7 cells; LPS+DMSO, HL-1 cells co-cultured with LPS-stimulated RAW264.7 cells and treated with DMSO; LPS+EPA, HL-1 cells co-cultured with LPS-stimulated RAW264.7 cells and treated with EPA.

Appendix Figure S2. EPA modulated immune-associated signaling pathways in cardiomyocytes co-cultured with M1 polarized macrophages. (**A**) Comparison of KEGG pathway enrichment between LPS+DMSO and LPS+EPA groups. (**B**) GO analysis for differential biological processes between LPS+DMSO and LPS+EPA groups. Abbreviations: KEGG, Kyoto Encyclopedia of Genes and Genomes; GO, Gene Ontology. Other abbreviations are the same as in Appendix Figure S1. Groups: LPS+DMSO, HL-1 cells co-cultured with LPS-stimulated RAW264.7 cells and treated with DMSO; LPS+EPA, HL-1 cells co-cultured with LPS-stimulated RAW264.7 cells and treated with EPA.

Appendix Figure S3. EPA had no profound effect on the induction of macrophage

M2 polarization. (**A-C**) mRNA expression of *Arg1*, *Tgf-β1* and *Il-10* in the RAW264.7 cells were measured by qRT-PCR. (**D, E**) Protein expression of ARG1 and TGF-β1 were determined by Western blot. (**F, G**) Immunofluorescence staining of CD206 (red) and F4/80 (green) with merged positive area quantified (bar=75 μm). The white arrows point to regions with CD206 and F4/80 fluorescence. Data information: For (**A-E, G**), the data are normalized to Ctrl. Data are represented as individual data points of $n = 3$ (**A-E, G**) biological replicates and means ± SEM. For (**A-E**), $^{**}P<0.01$, $^{***}P<0.001$, LPS vs. Ctrl; $^{\dagger}P<0.05$, $^{\dagger\dagger}P<0.01$, $^{\dagger\dagger\dagger}P<0.001$, LPS+EPA vs. LPS+DMSO; ns, not significant. Analysis by one-way ANOVA. For (**G**), $^{**}P<0.01$, DM vs. Ctrl; ns, not significant. Analysis by one-way ANOVA. Abbreviations: *Arg1*/ARG1, arginase 1; DAPI, 4',6-diamidino-2-phenylindole; *Il-10*, interleukin-10; *Tgf-β1*/TGF-β1, transforming factor-beta 1. Other abbreviations are the same as in Appendix Figure S1. Groups: For (**A-E**), Ctrl, control; LPS, LPS-stimulated RAW264.7 cells; LPS+DMSO, LPS-stimulated RAW264.7 cells treated with DMSO; LPS+EPA, LPS-stimulated RAW264.7 cells treated with EPA. For (**F, G**), Ctrl, control; DM, diabetes mellitus; DM/EPA, diabetic mice supplemented with EPA.

Appendix Figure S4. EPA promoted M2 polarization *in vitro.* (**A**) Schematic representation of the experimental protocol. M2 polarization was induced in RAW264.7 cells using IL-4 and IL-13 *in vitro*. (**B-D**) Protein expression of ARG1, TGF-β1 and IL-10 were determined by Western blot. Data information: For (**B-D**), the data are normalized to Ctrl. Data are represented as individual data points of $n = 3$ (**B-D**) biological replicates and means ± SEM. $^{*}P<0.05$, $^{**}P<0.01$, IL-4+IL-13 vs. Ctrl; $^{\dagger}P<0.05$, $^{\dagger\dagger}P<0.01$, $^{\dagger\dagger\dagger}P<0.001$, IL-4+IL-13+EPA vs. IL-4+IL-13+DMSO. Analysis by one-way ANOVA. Abbreviations: IL-4, interleukin-4; IL-13, interleukin-13. Other abbreviations are the same as in Appendix Figures S1 and S3. Groups: Ctrl, control; IL-4+IL-13, IL-4 and IL-13-stimulated RAW264.7 cells; IL-4+IL-13+DMSO, IL-4 and IL-13-stimulated RAW264.7 cells treated with DMSO; IL-4+IL-13+EPA, IL-4 and IL-13-stimulated RAW264.7 cells treated with EPA.

Appendix Figure S5. ox-PAPC established Mox polarization of macrophages. (**A-**

C) mRNA expression of *Hmox1, Srxn1* and *TrxR1*. (**D, E**) Protein expression of HO-1 and TrxR1. Data information: For (**A-E**), the data are normalized to Ctrl. Data are represented as individual data points of $n = 3$ (**A-E**) biological replicates and means ± SEM. **P<0.01, ***P<0.001, ox-PAPC vs. Ctrl. Analysis by two-tailed unpaired Student's t-test. Abbreviations: *Hmox1*/HO-1, heme oxygenase 1; ox-PAPC, oxidized 1-palmitoyl-2-arachidonoyl-sn-glycero-3-phosphocholine; *Srxn1*, sulfiredoxin-1; *TrxR1*/TrxR1, thioredoxin reductase 1. Groups: Ctrl, control; ox-PAPC, ox-PAPC-treated RAW264.7 cells.

Appendix Figure S6. HO-1 maintained the Mox phenotype of macrophages. To verify the efficacy of gene silencing, (**A, B**) mRNA and protein levels of *Hmox1* were determined in RAW264.7 cells. To study the effect of HO-1 in the maintenance of macrophage Mox phenotype, (**C, D**) protein levels of HO-1 and TrxR1 were measured in RAW264.7 cells. Data information: For (**A, B**), the data are normalized to Ctrl. For (**C, D**), the data are normalized to ox-PAPC+si-NC. Data are represented as individual data points of $n = 3$ (**A-D**) biological replicates and means ± SEM. For (**A, B**), ***P<0.001, si-*Hmox1* vs. si-NC; ns, not significant. Analysis by one-way ANOVA; For (**C, D**), $^{††}P$<0.01, ox-PAPC+ si-*Hmox1* vs. ox-PAPC+si-NC. Analysis by two-tailed unpaired Student's t-test. Abbreviations: si-*Hmox1*, *Hmox1* siRNA; si-NC, negative control siRNA. Other abbreviations are the same as in Appendix Figure S5. Groups: Ctrl, control; si-NC, negative control siRNA for *Hmox1*; si-*Hmox1*, *Hmox1* siRNA; ox-PAPC+si-NC, ox-PAPC-induced Mox polarized RAW264.7 cells were transfected with si-NC; ox-PAPC+si-*Hmox1*, ox-PAPC-induced Mox polarized RAW264.7 cells were transfected with si-*Hmox1*.

Appendix Figure S7. HO-1 played a critical role in EPA inhibition of oxidative stress in M1 polarized macrophages. (**A**) mRNA expression of *Nos2*. (**B, C**) Protein expression of COX2 and NOX4. (**D, E**) DHE assay (bar=100 μm) by double staining with DAPI (blue) and DHE (red), and ratio of DHE positive cells. Data information: For (**A-C, E**), the data are normalized to LPS+DMSO. Data are represented as individual data points of $n = 3$ (**A-C, E**) biological replicates and means ± SEM.

**P<0.01, ***P<0.001, LPS+EPA vs. LPS+DMSO; $^{\dagger}P$<0.05, $^{\dagger\dagger}P$<0.01, $^{\dagger\dagger\dagger}P$<0.001, LPS+EPA+si-*Hmox1* vs. LPS+EPA+si-NC. Analysis by one-way ANOVA. Abbreviations: COX2, cyclooxygenase-2; DHE, dihydroethidium; *Nos2*, nitric oxide synthase 2; *Nox4*/NOX4, NADPH oxidase 4. Other abbreviations are the same as in Appendix Figures S1, S3 and S6. Groups: LPS+DMSO, LPS-stimulated RAW264.7 cells treated with DMSO; LPS+EPA, LPS-stimulated RAW264.7 cells treated with EPA; LPS+EPA+si-NC, LPS-stimulated RAW264.7 cells treated with EPA and si-NC; LPS+EPA+si-*Hmox1*, LPS-stimulated RAW264.7 cells treated with EPA and si-*Hmox1*.

Appendix Figure S8. EPA had no profound effect on GPR40 protein levels of M1 polarized macrophages. Protein levels of GPR40 and HO-1. Data information: The data are normalized to Ctrl. Data are represented as individual data points of $n = 3$ biological replicates and means ± SEM. ns, not significant. Analysis by two-tailed unpaired Student's t-test. Abbreviations: GPR40, G-protein-coupled receptor 40; Other abbreviations are the same as in Appendix Figure S1. Groups: Ctrl, control; LPS, LPS-stimulated RAW264.7 cells; LPS+DMSO, LPS-stimulated RAW264.7 cells treated with DMSO; LPS+EPA, LPS-stimulated RAW264.7 cells treated with EPA.
