# Supplementary figures and images for: Eicosapentaenoic acid induces macrophage Mox polarization to prevent diabetic cardiomyopathy (part 1 of 2)
Source: EMBO Rep. 2024 Oct 31;25(12):5507–36. doi: 10.1038/s44319-024-00271-x (PMC11624267; doi:10.1038/s44319-024-00271-x)

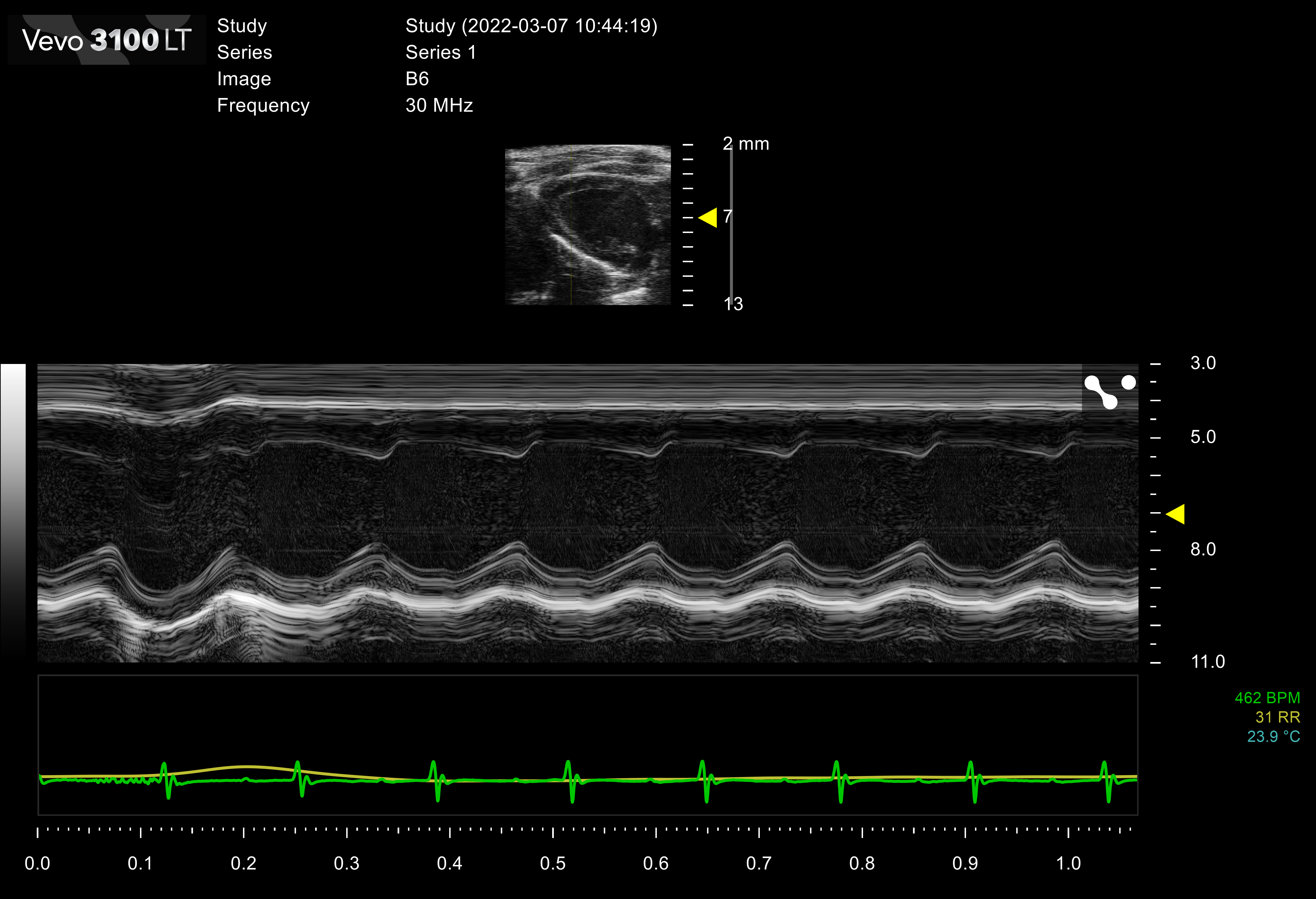

Supplement: Supplementary file 3 — Source data Fig. 1 [file 44319_2024_271_MOESM3_ESM.zip › Figure 1/Fig. 1A M mode-Ctrl group.tif]

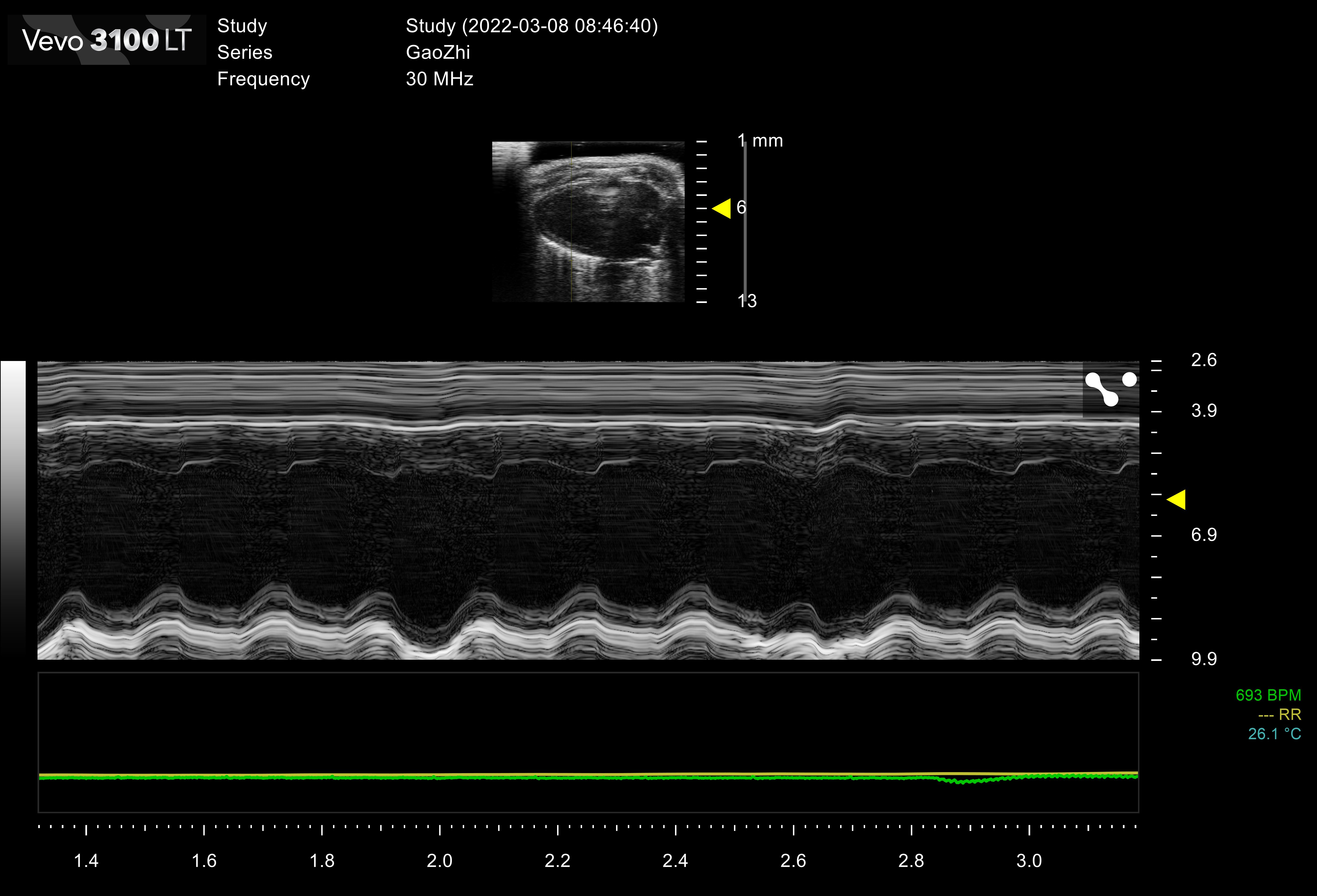

Supplement: Supplementary file 3 — Source data Fig. 1 [file 44319_2024_271_MOESM3_ESM.zip › Figure 1/Fig. 1A M mode-DM group.tif]

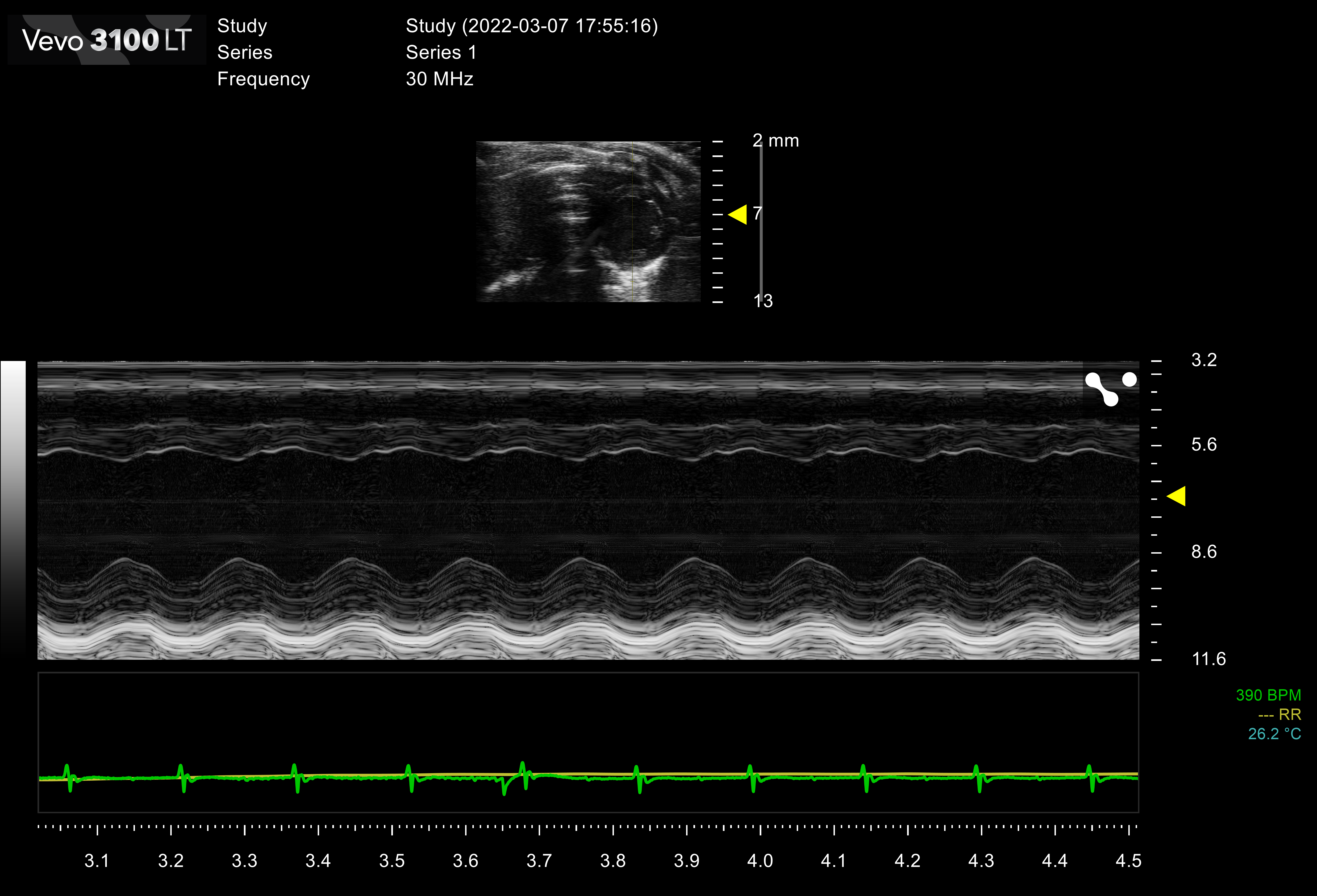

Supplement: Supplementary file 3 — Source data Fig. 1 [file 44319_2024_271_MOESM3_ESM.zip › Figure 1/Fig. 1A M mode-DM&EPA group.tif]

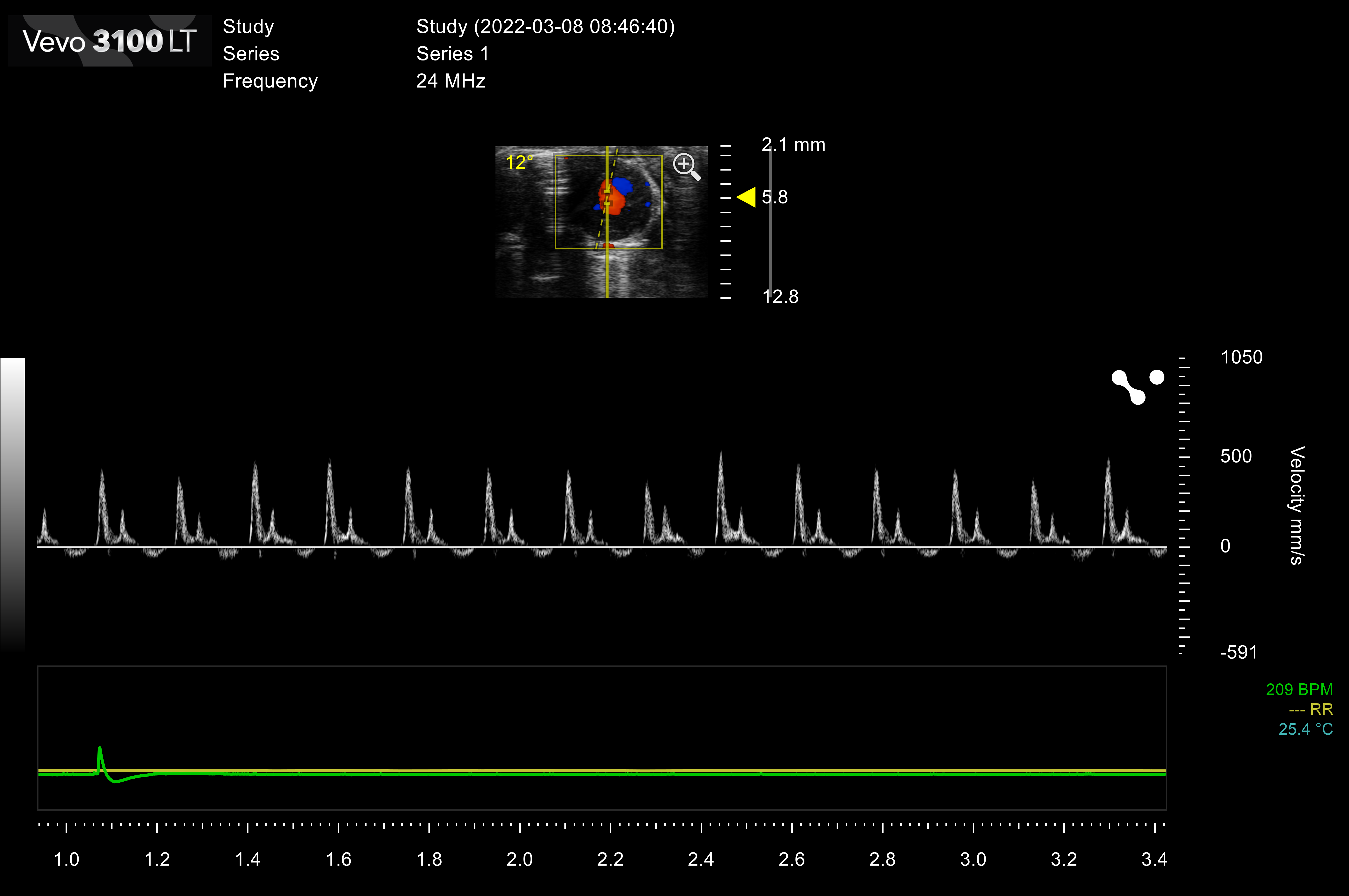

Supplement: Supplementary file 3 — Source data Fig. 1 [file 44319_2024_271_MOESM3_ESM.zip › Figure 1/Fig. 1A PW Doppler mode-Ctrl group.tif]

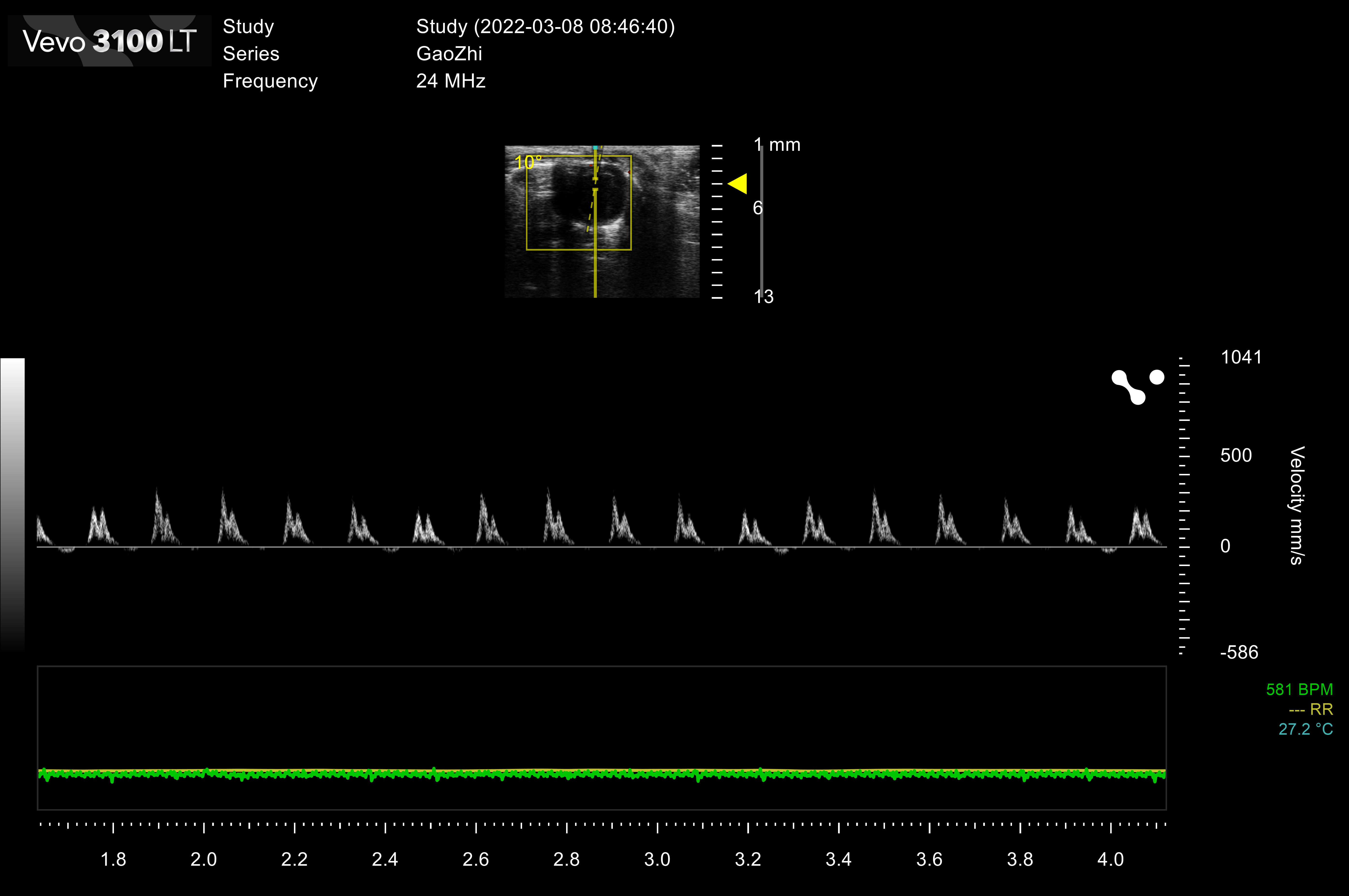

Supplement: Supplementary file 3 — Source data Fig. 1 [file 44319_2024_271_MOESM3_ESM.zip › Figure 1/Fig. 1A PW Doppler mode-DM group.tif]

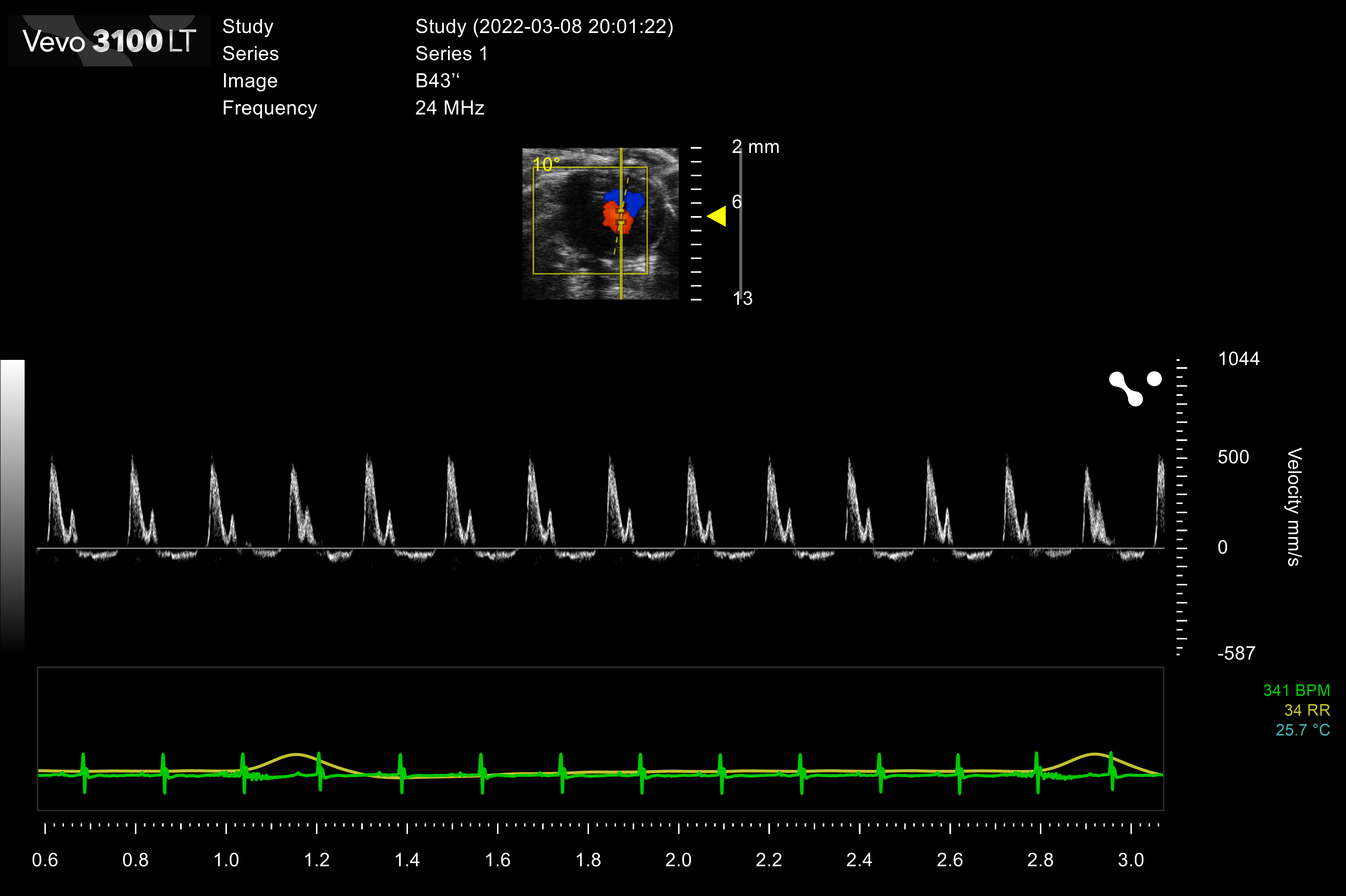

Supplement: Supplementary file 3 — Source data Fig. 1 [file 44319_2024_271_MOESM3_ESM.zip › Figure 1/Fig. 1A PW Doppler mode-DM&EPA group.tif]

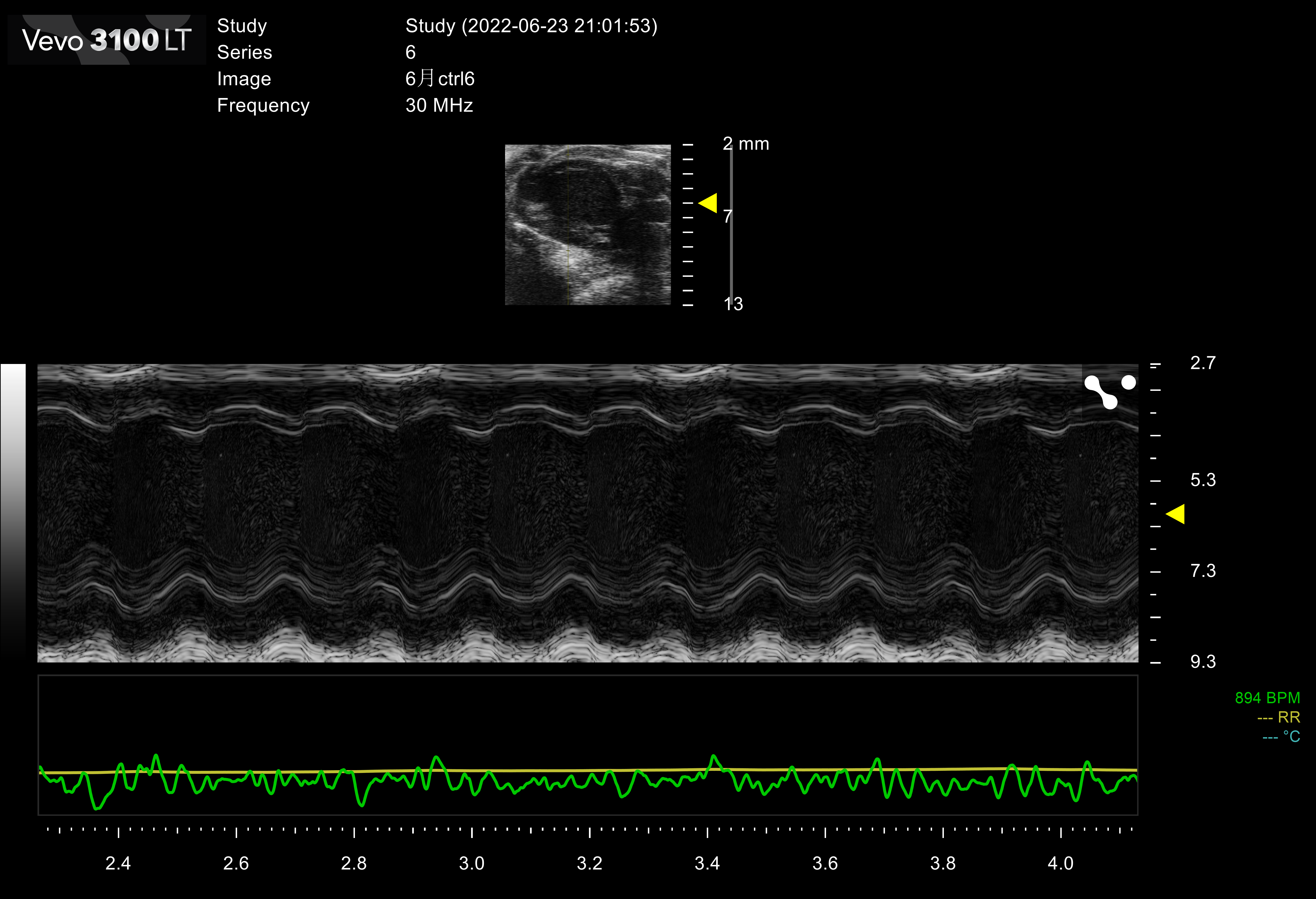

Supplement: Supplementary file 3 — Source data Fig. 1 [file 44319_2024_271_MOESM3_ESM.zip › Figure 1/Fig. 1E M mode-Ctrl group.tif]

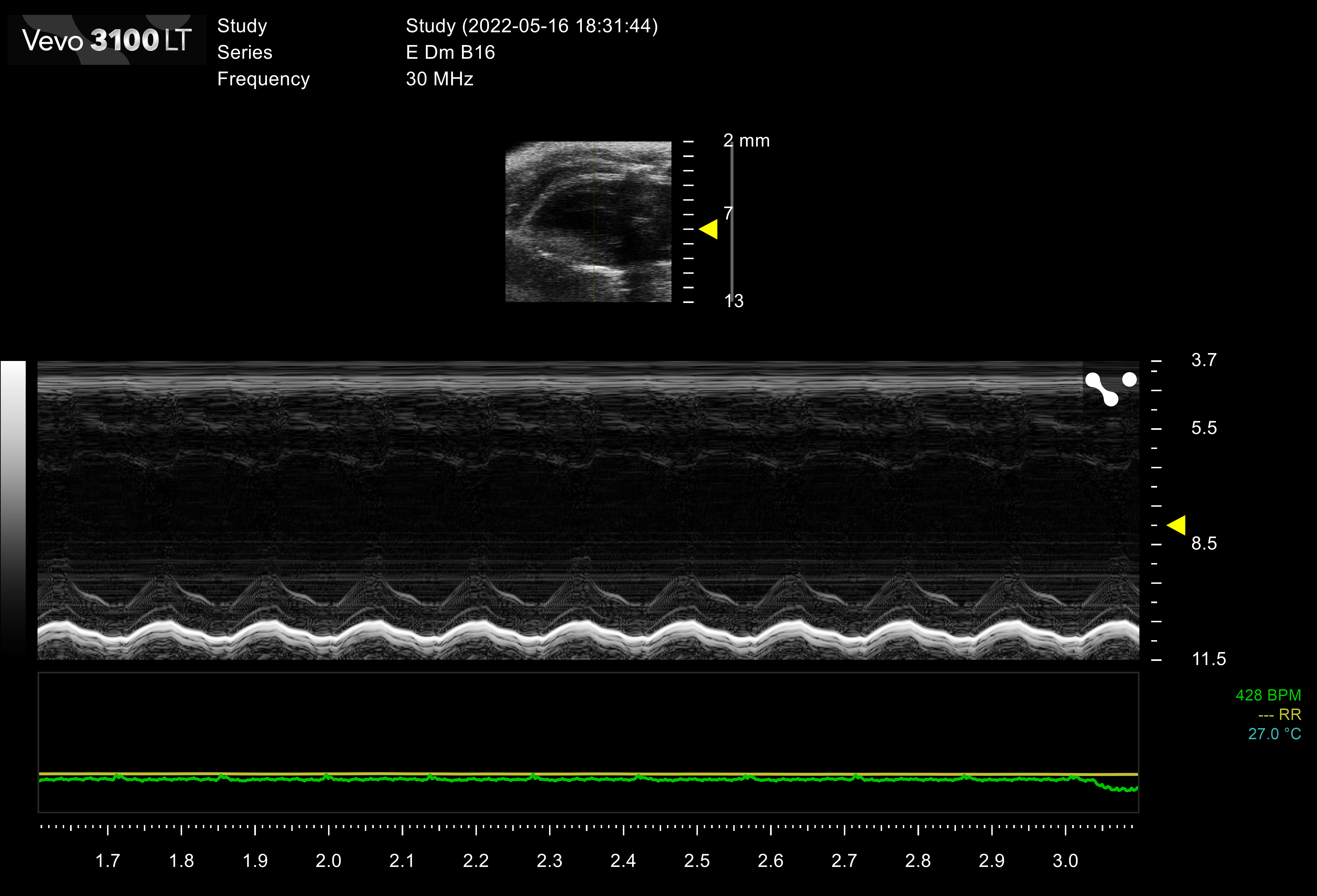

Supplement: Supplementary file 3 — Source data Fig. 1 [file 44319_2024_271_MOESM3_ESM.zip › Figure 1/Fig. 1E M mode-DM group.tif]

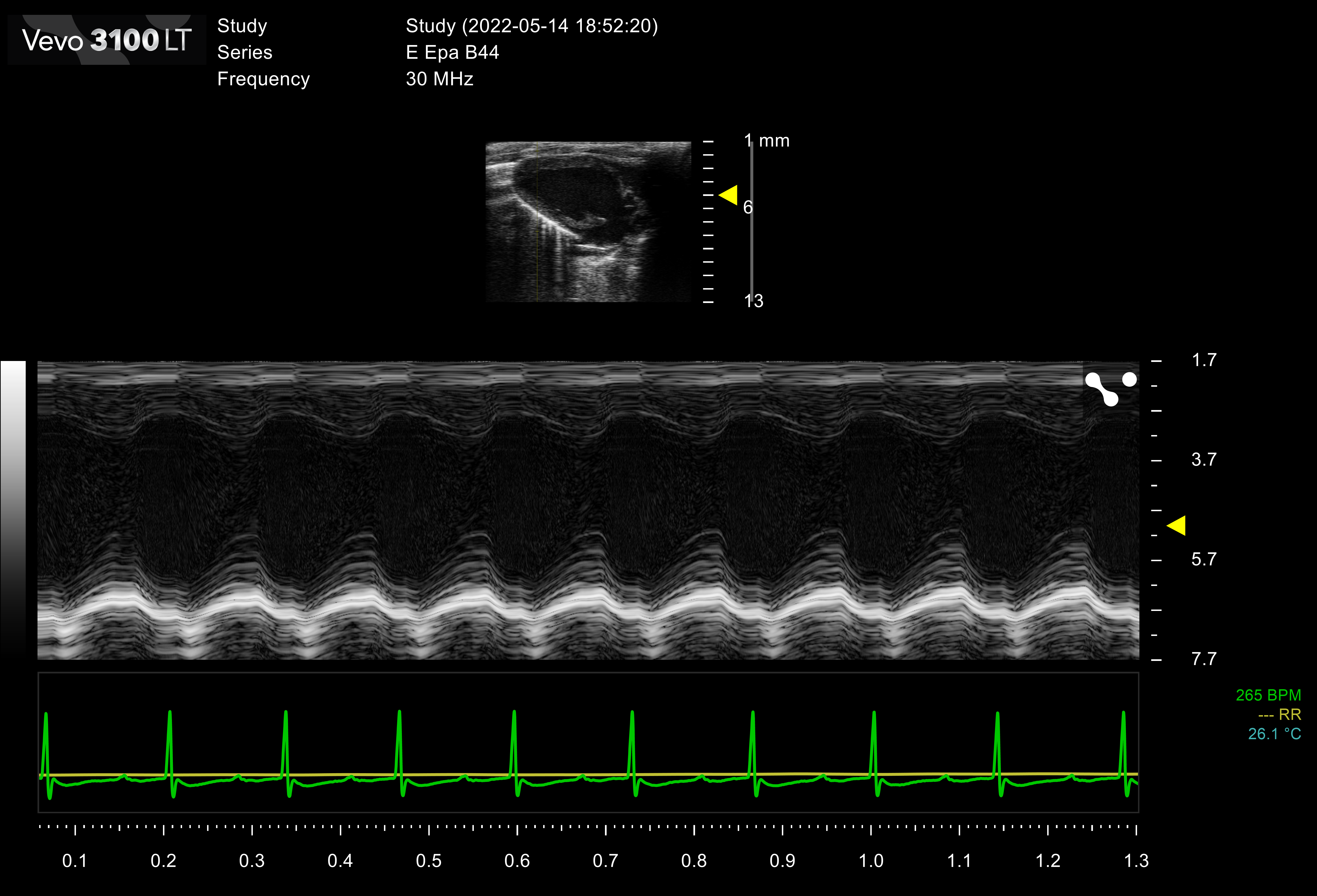

Supplement: Supplementary file 3 — Source data Fig. 1 [file 44319_2024_271_MOESM3_ESM.zip › Figure 1/Fig. 1E M mode-DM&EPA group.tif]

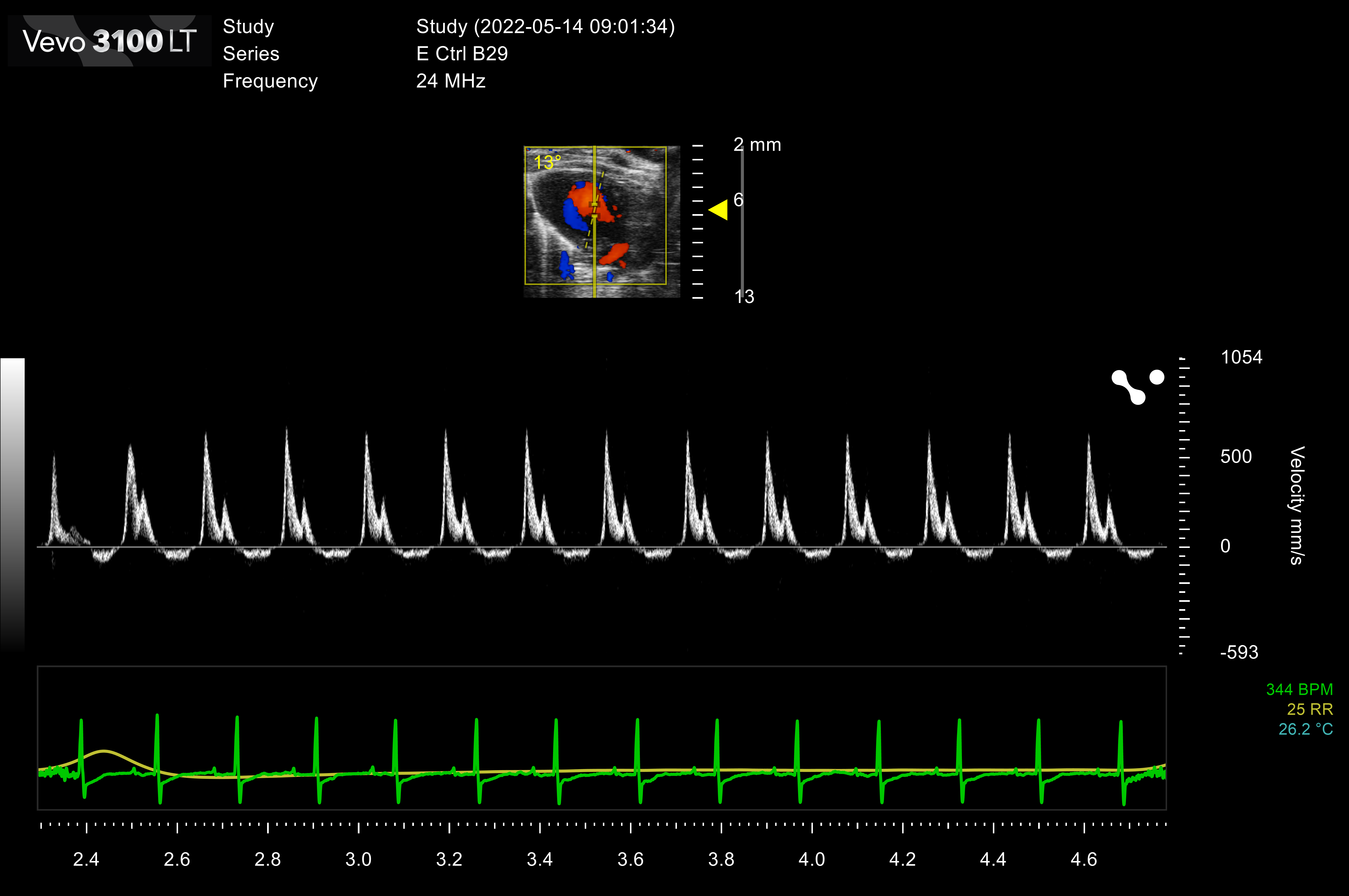

Supplement: Supplementary file 3 — Source data Fig. 1 [file 44319_2024_271_MOESM3_ESM.zip › Figure 1/Fig. 1E PW Doppler mode-Ctrl group.tif]

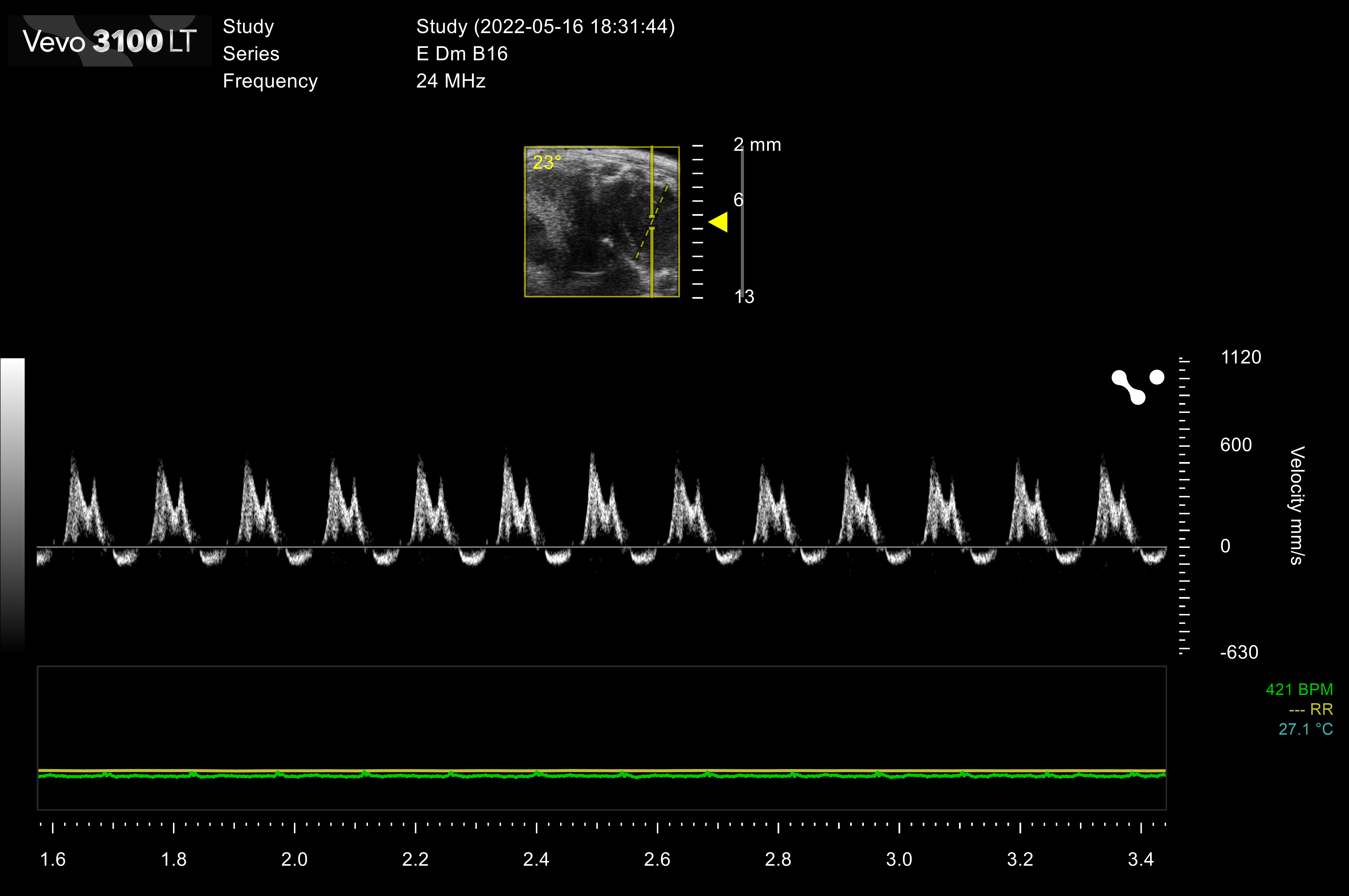

Supplement: Supplementary file 3 — Source data Fig. 1 [file 44319_2024_271_MOESM3_ESM.zip › Figure 1/Fig. 1E PW Doppler mode-DM group.tif]

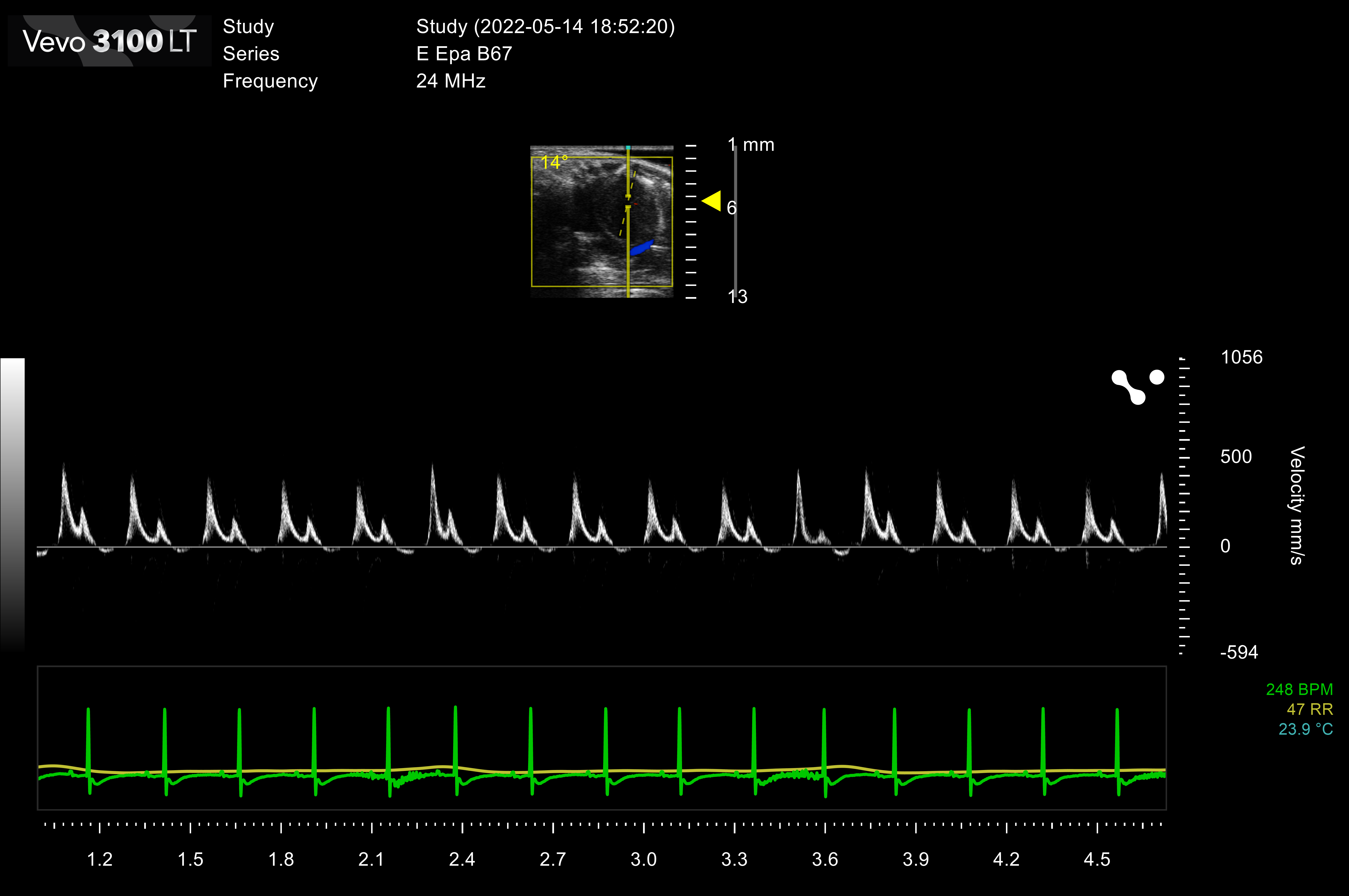

Supplement: Supplementary file 3 — Source data Fig. 1 [file 44319_2024_271_MOESM3_ESM.zip › Figure 1/Fig. 1E PW Doppler mode-DM&EPA group.tif]

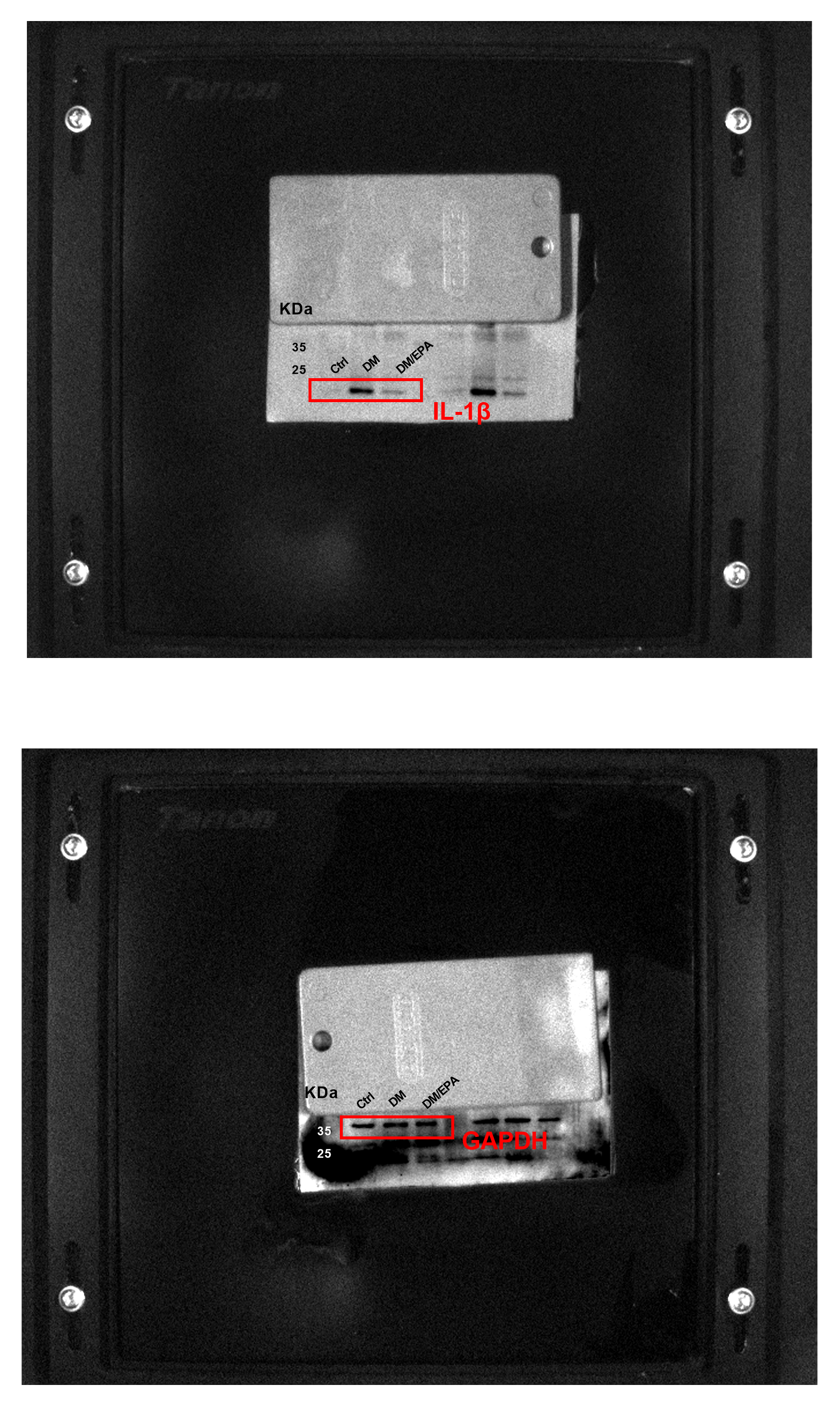

Supplement: Supplementary file 4 — Source data Fig. 2 [file 44319_2024_271_MOESM4_ESM.zip › Figure 2/Fig. 2D IL-1¦Â&GAPDH.tif]

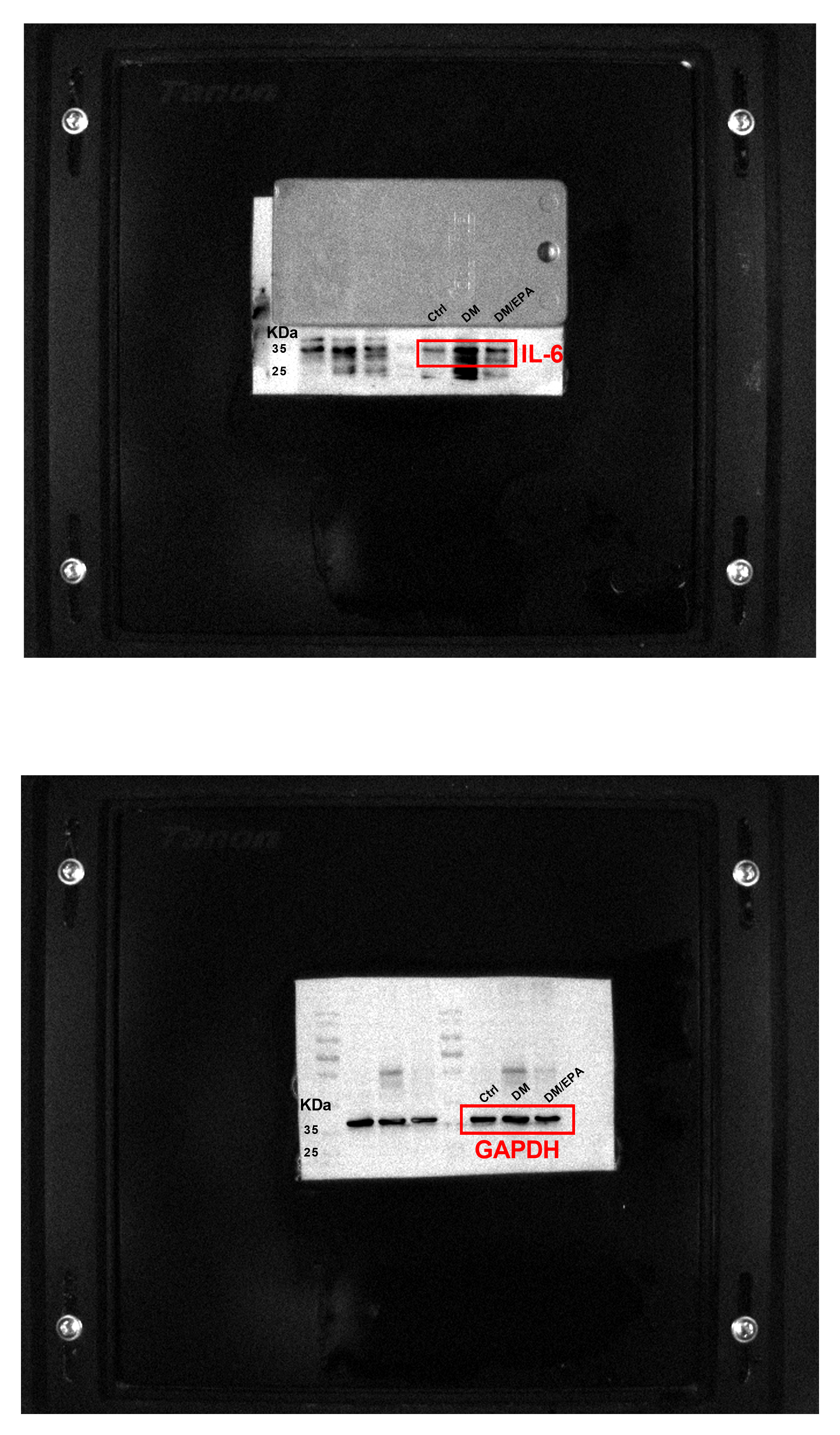

Supplement: Supplementary file 4 — Source data Fig. 2 [file 44319_2024_271_MOESM4_ESM.zip › Figure 2/Fig. 2E IL-6&GAPDH.tif]

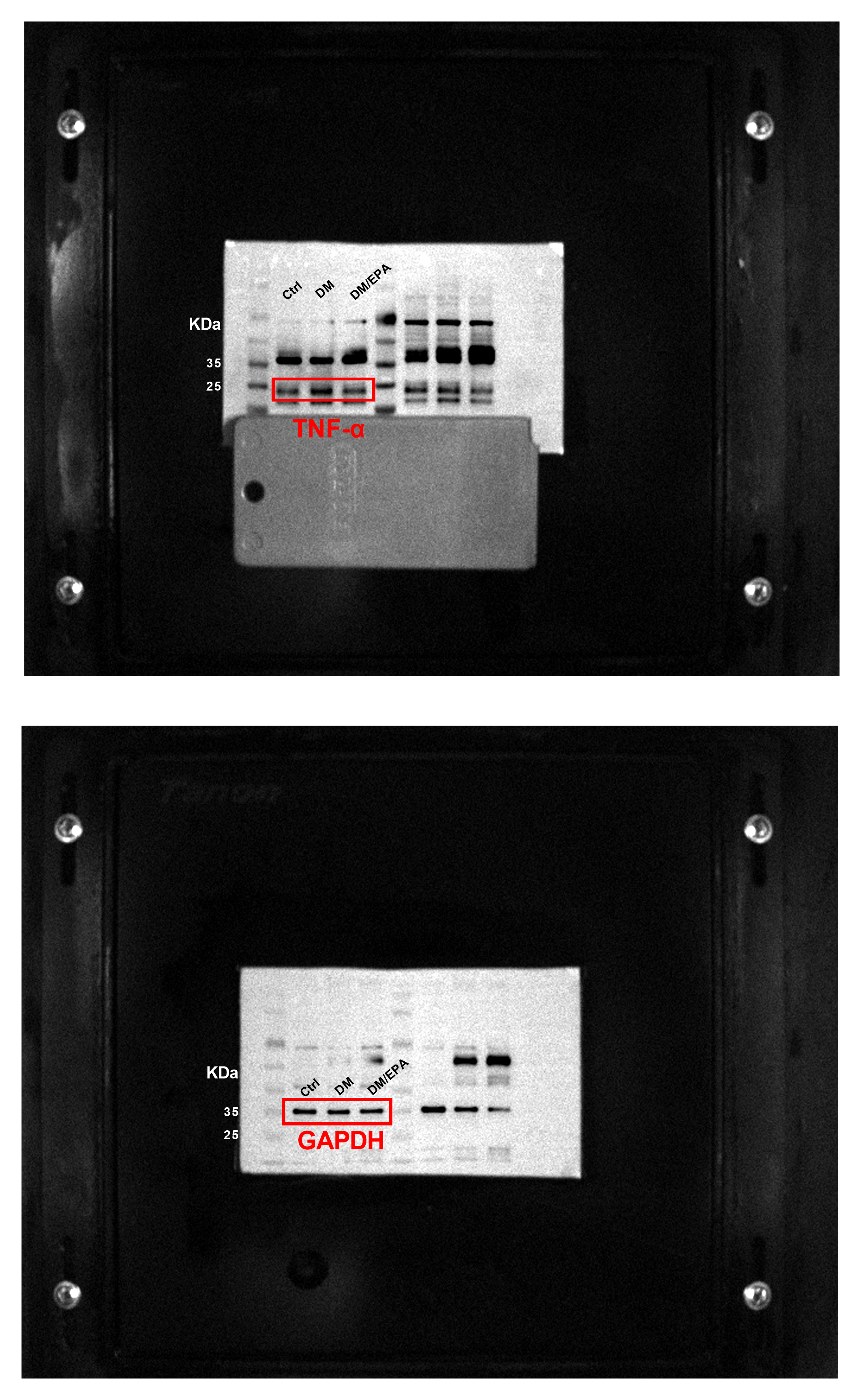

Supplement: Supplementary file 4 — Source data Fig. 2 [file 44319_2024_271_MOESM4_ESM.zip › Figure 2/Fig. 2F TNF-¦Á&GAPDH.tif]

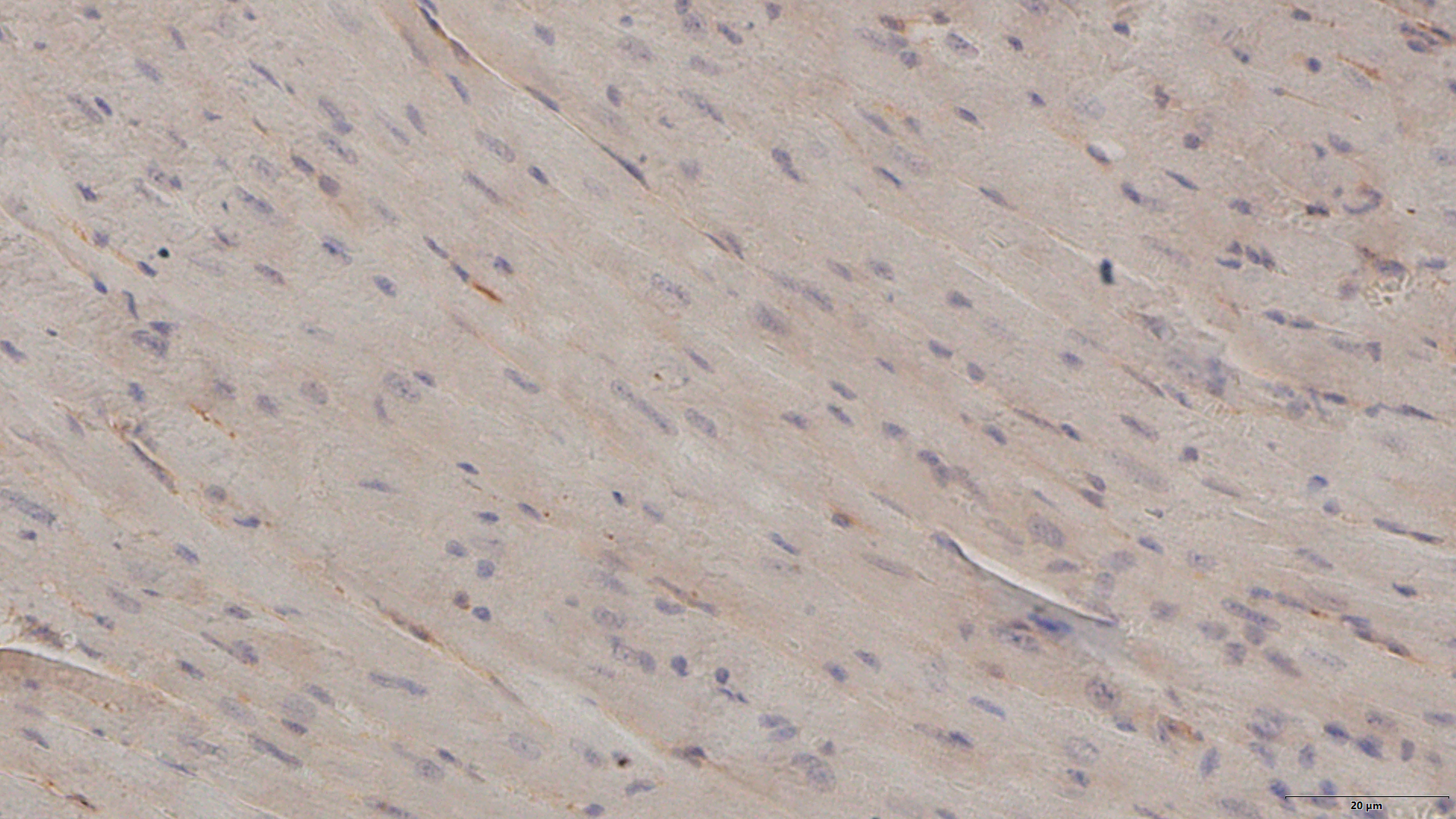

Supplement: Supplementary file 4 — Source data Fig. 2 [file 44319_2024_271_MOESM4_ESM.zip › Figure 2/Fig. 2G IL-1¦Â-Ctrl group.tif]

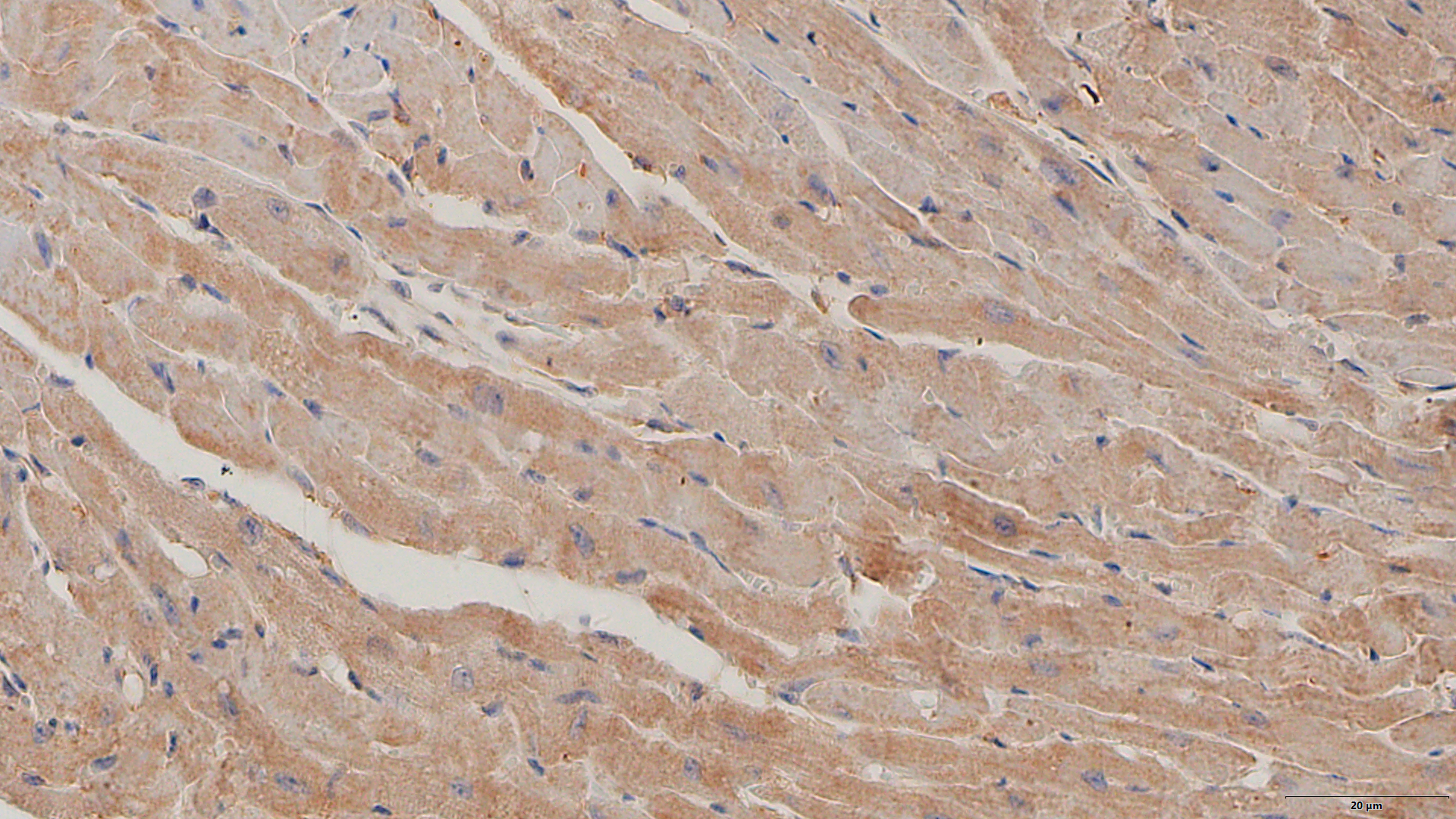

Supplement: Supplementary file 4 — Source data Fig. 2 [file 44319_2024_271_MOESM4_ESM.zip › Figure 2/Fig. 2G IL-1¦Â-DM group.tif]

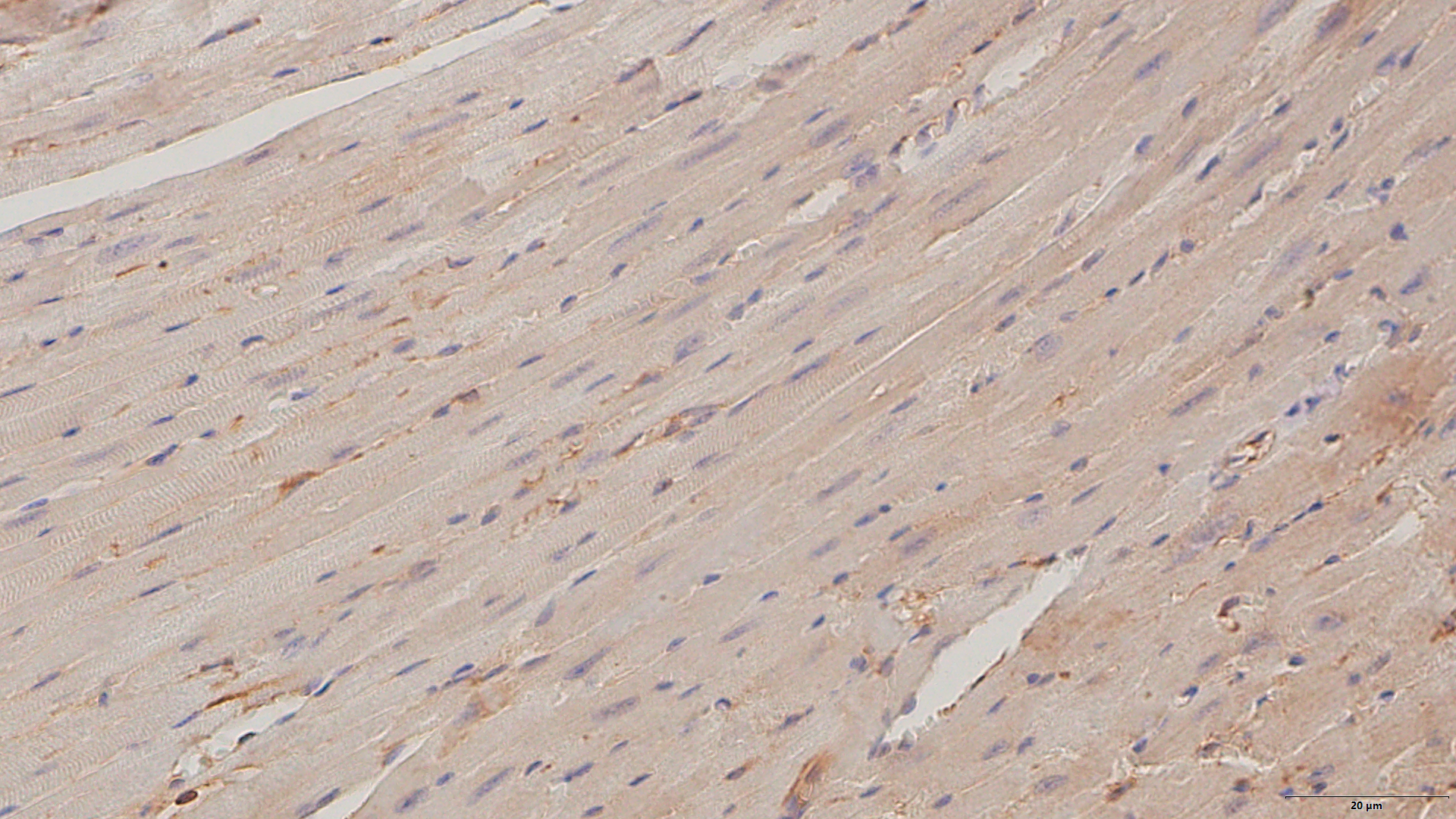

Supplement: Supplementary file 4 — Source data Fig. 2 [file 44319_2024_271_MOESM4_ESM.zip › Figure 2/Fig. 2G IL-1¦Â-DM&EPA group.tif]

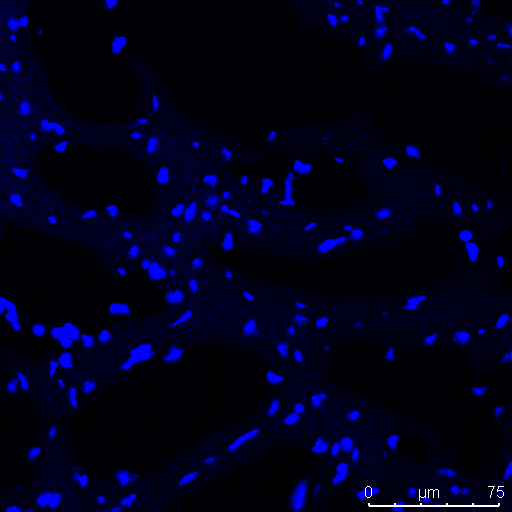

Supplement: Supplementary file 4 — Source data Fig. 2 [file 44319_2024_271_MOESM4_ESM.zip › Figure 2/Fig. 2I Ctrl-DAPI.tif]

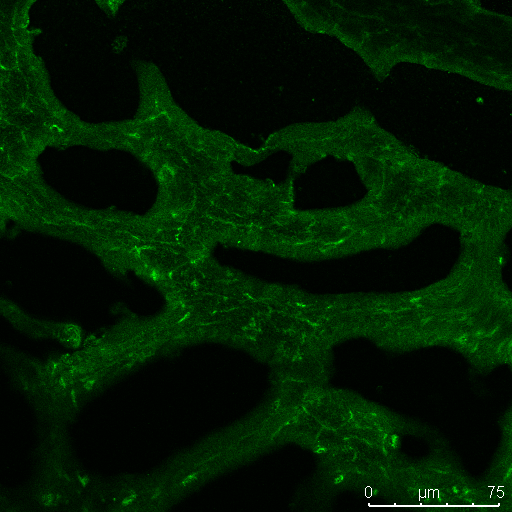

Supplement: Supplementary file 4 — Source data Fig. 2 [file 44319_2024_271_MOESM4_ESM.zip › Figure 2/Fig. 2I Ctrl-F480.tif]

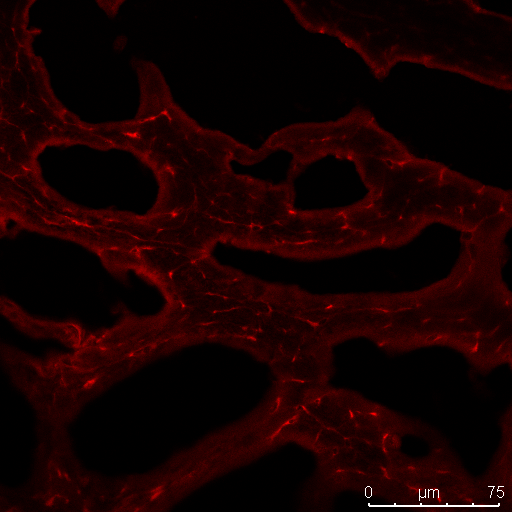

Supplement: Supplementary file 4 — Source data Fig. 2 [file 44319_2024_271_MOESM4_ESM.zip › Figure 2/Fig. 2I Ctrl-INOS.tif]

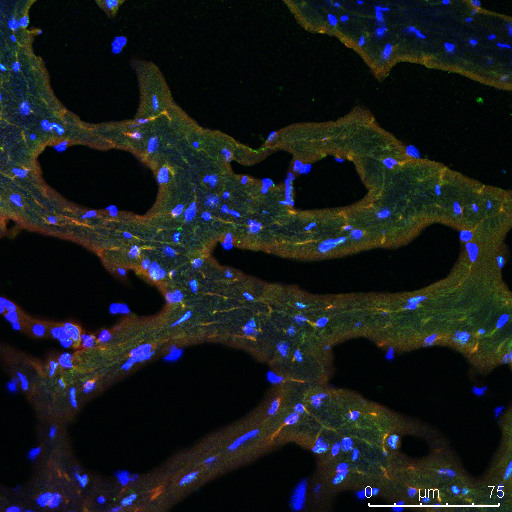

Supplement: Supplementary file 4 — Source data Fig. 2 [file 44319_2024_271_MOESM4_ESM.zip › Figure 2/Fig. 2I Ctrl-Merge.tif]

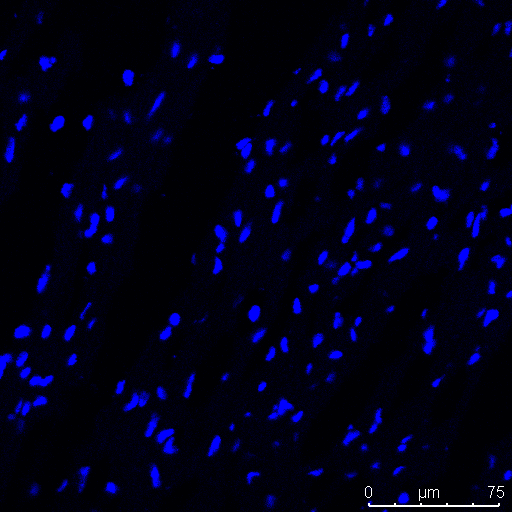

Supplement: Supplementary file 4 — Source data Fig. 2 [file 44319_2024_271_MOESM4_ESM.zip › Figure 2/Fig. 2I DM&EPA-DAPI.tif]

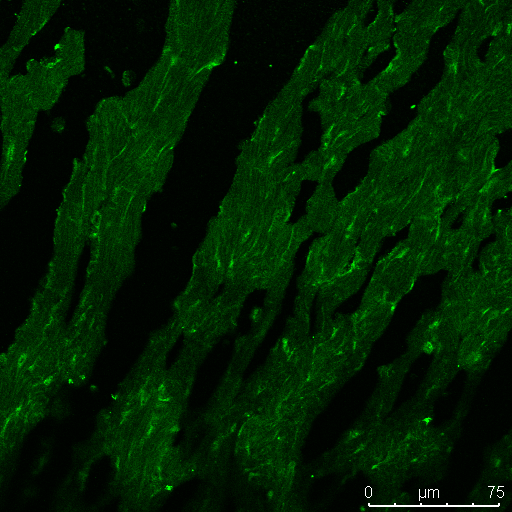

Supplement: Supplementary file 4 — Source data Fig. 2 [file 44319_2024_271_MOESM4_ESM.zip › Figure 2/Fig. 2I DM&EPA-F480.tif]

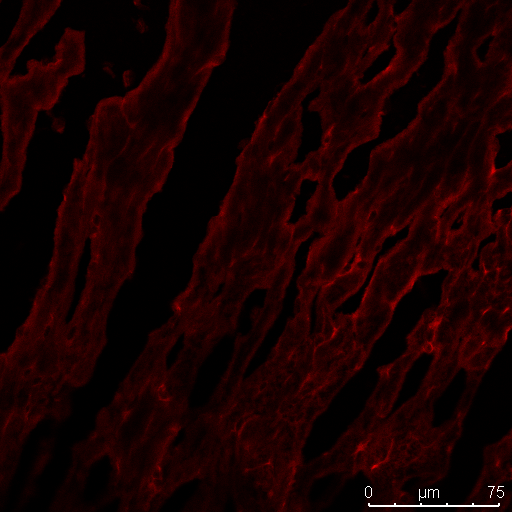

Supplement: Supplementary file 4 — Source data Fig. 2 [file 44319_2024_271_MOESM4_ESM.zip › Figure 2/Fig. 2I DM&EPA-INOS.tif]

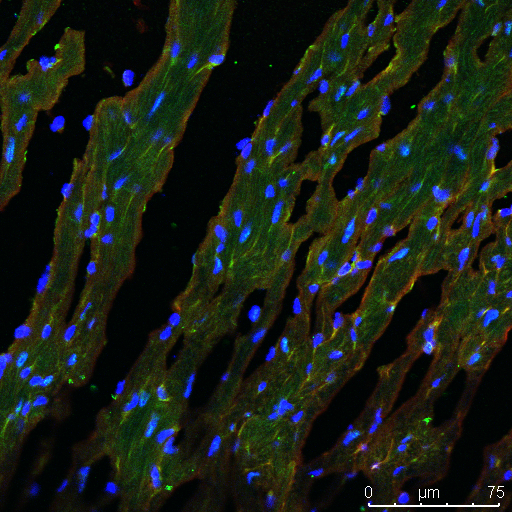

Supplement: Supplementary file 4 — Source data Fig. 2 [file 44319_2024_271_MOESM4_ESM.zip › Figure 2/Fig. 2I DM&EPA-Merge.tif]

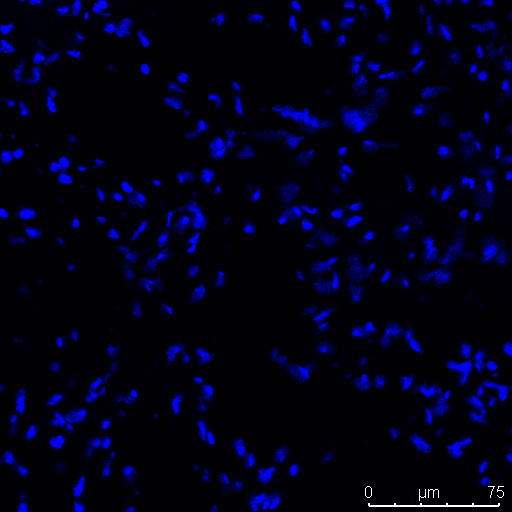

Supplement: Supplementary file 4 — Source data Fig. 2 [file 44319_2024_271_MOESM4_ESM.zip › Figure 2/Fig. 2I DM-DAPI.tif]

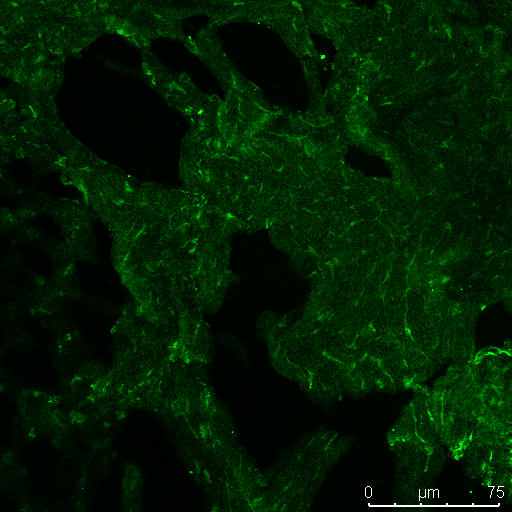

Supplement: Supplementary file 4 — Source data Fig. 2 [file 44319_2024_271_MOESM4_ESM.zip › Figure 2/Fig. 2I DM-F480.tif]

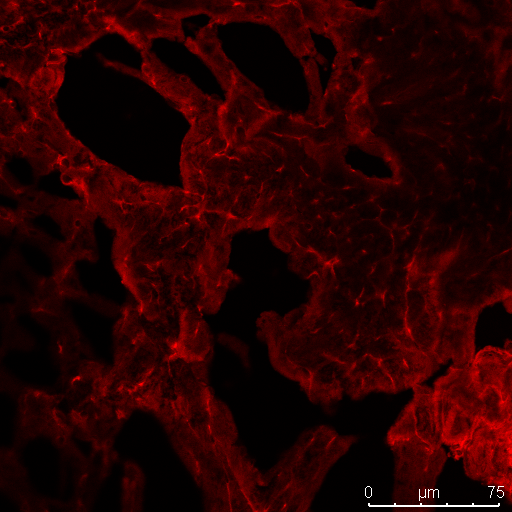

Supplement: Supplementary file 4 — Source data Fig. 2 [file 44319_2024_271_MOESM4_ESM.zip › Figure 2/Fig. 2I DM-INOS.tif]

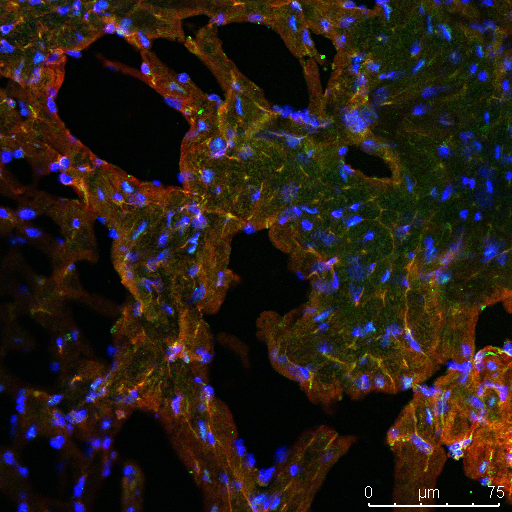

Supplement: Supplementary file 4 — Source data Fig. 2 [file 44319_2024_271_MOESM4_ESM.zip › Figure 2/Fig. 2I DM-Merge.tif]

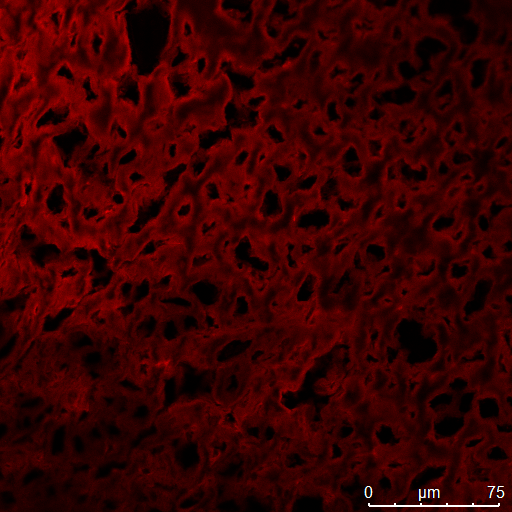

Supplement: Supplementary file 4 — Source data Fig. 2 [file 44319_2024_271_MOESM4_ESM.zip › Figure 2/Fig. 2J Ctrl-CD86.tif]

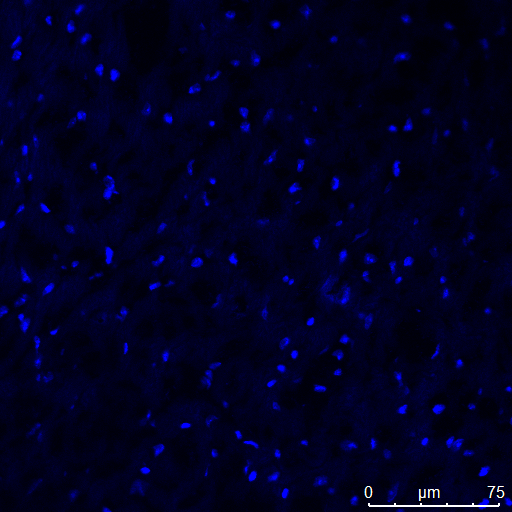

Supplement: Supplementary file 4 — Source data Fig. 2 [file 44319_2024_271_MOESM4_ESM.zip › Figure 2/Fig. 2J Ctrl-DAPI.tif]

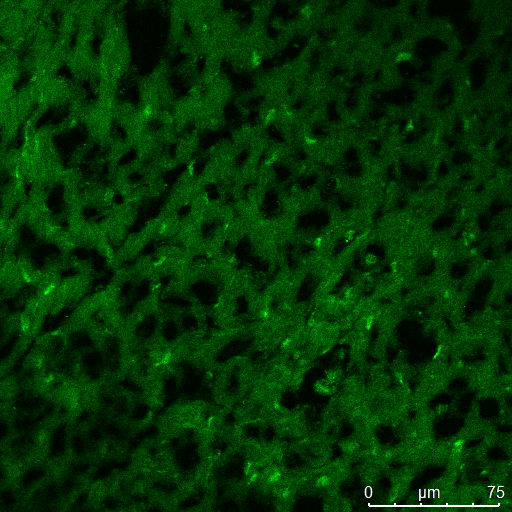

Supplement: Supplementary file 4 — Source data Fig. 2 [file 44319_2024_271_MOESM4_ESM.zip › Figure 2/Fig. 2J Ctrl-F480.tif]

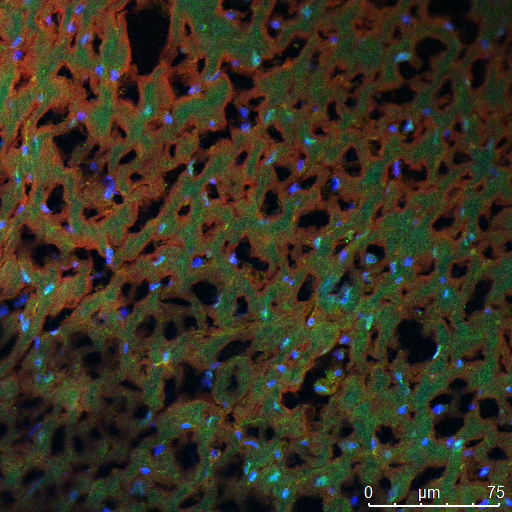

Supplement: Supplementary file 4 — Source data Fig. 2 [file 44319_2024_271_MOESM4_ESM.zip › Figure 2/Fig. 2J Ctrl-Merge.tif]

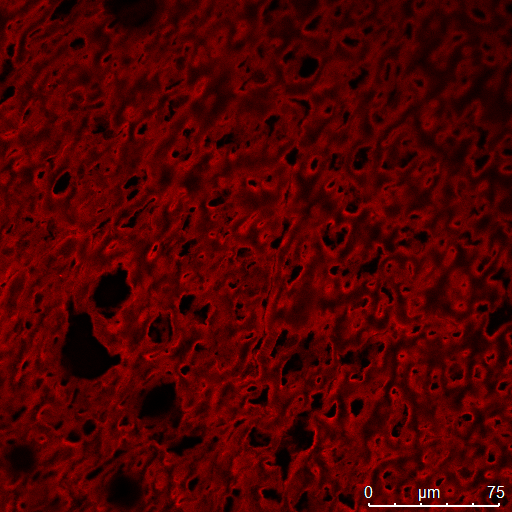

Supplement: Supplementary file 4 — Source data Fig. 2 [file 44319_2024_271_MOESM4_ESM.zip › Figure 2/Fig. 2J DM&EPA-CD86.tif]

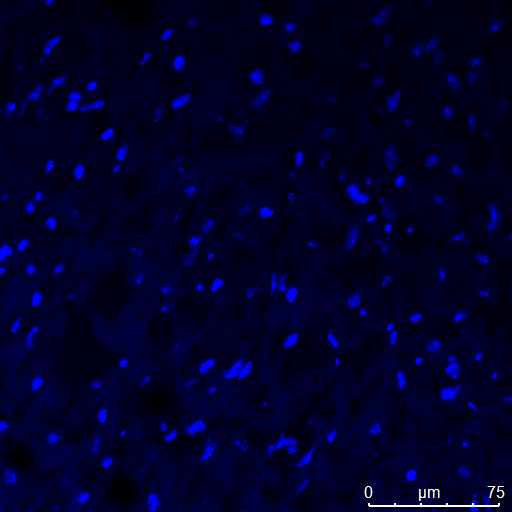

Supplement: Supplementary file 4 — Source data Fig. 2 [file 44319_2024_271_MOESM4_ESM.zip › Figure 2/Fig. 2J DM&EPA-DAPI.tif]

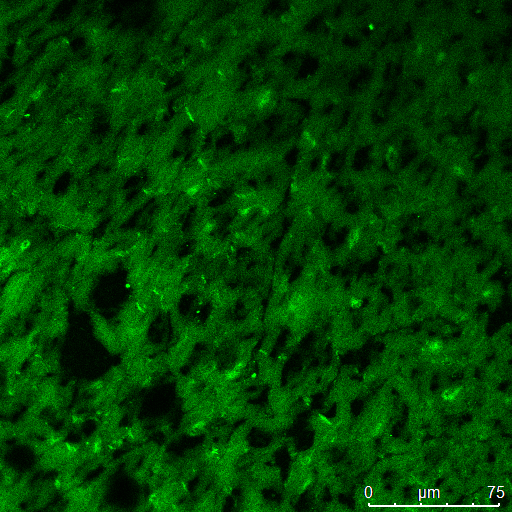

Supplement: Supplementary file 4 — Source data Fig. 2 [file 44319_2024_271_MOESM4_ESM.zip › Figure 2/Fig. 2J DM&EPA-F480.tif]

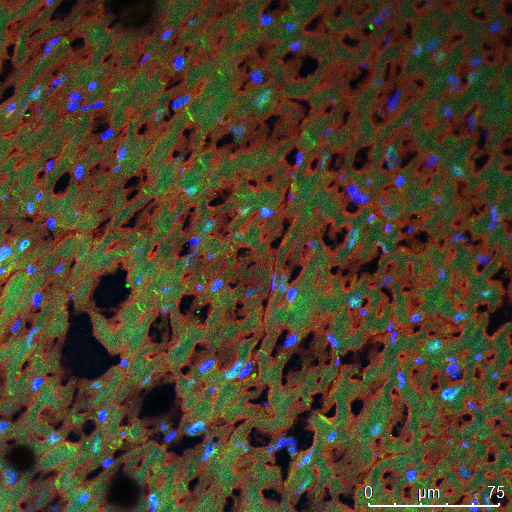

Supplement: Supplementary file 4 — Source data Fig. 2 [file 44319_2024_271_MOESM4_ESM.zip › Figure 2/Fig. 2J DM&EPA-Merge.tif]

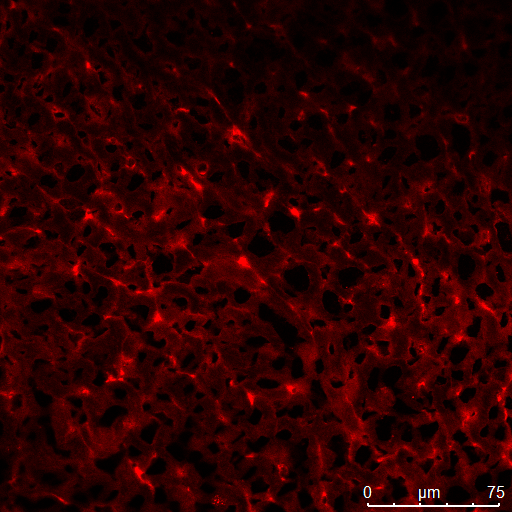

Supplement: Supplementary file 4 — Source data Fig. 2 [file 44319_2024_271_MOESM4_ESM.zip › Figure 2/Fig. 2J DM-CD86.tif]

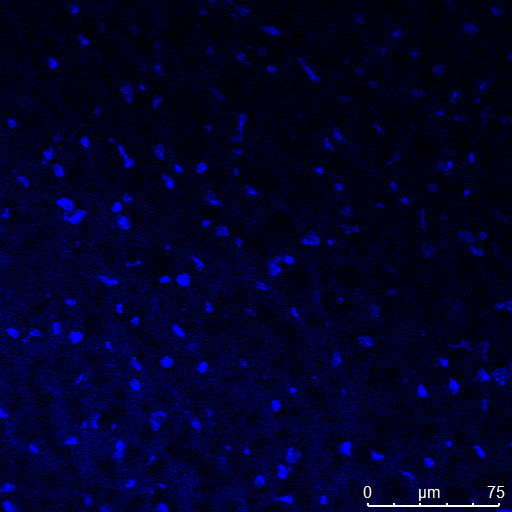

Supplement: Supplementary file 4 — Source data Fig. 2 [file 44319_2024_271_MOESM4_ESM.zip › Figure 2/Fig. 2J DM-DAPI.tif]

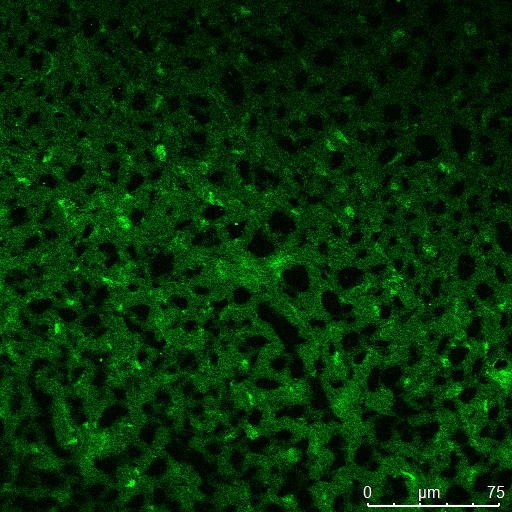

Supplement: Supplementary file 4 — Source data Fig. 2 [file 44319_2024_271_MOESM4_ESM.zip › Figure 2/Fig. 2J DM-F480.tif]

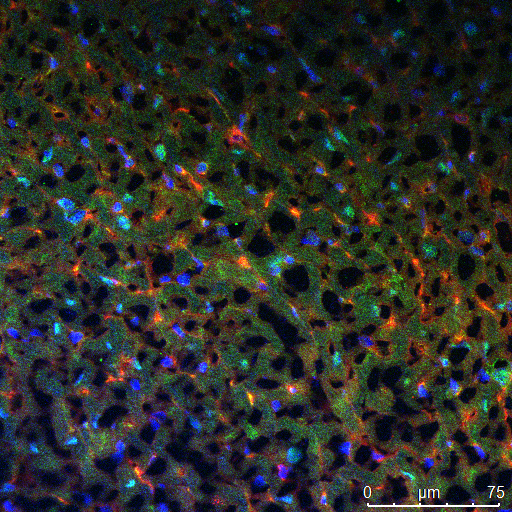

Supplement: Supplementary file 4 — Source data Fig. 2 [file 44319_2024_271_MOESM4_ESM.zip › Figure 2/Fig. 2J DM-Merge.tif]

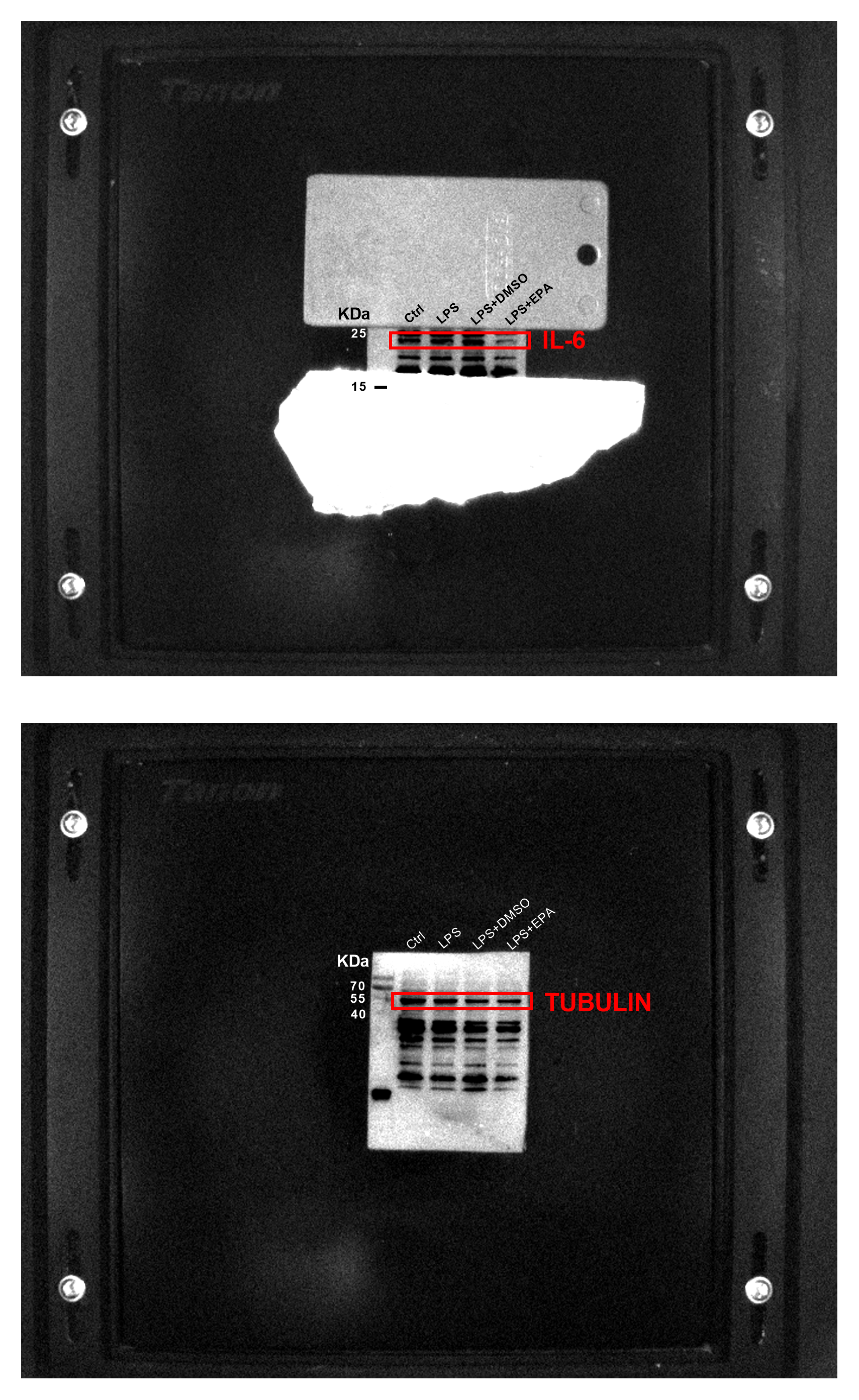

Supplement: Supplementary file 5 — Source data Fig. 3 [file 44319_2024_271_MOESM5_ESM.zip › Figure 3/Fig. 3A IL-6&TUBULIN.tif]

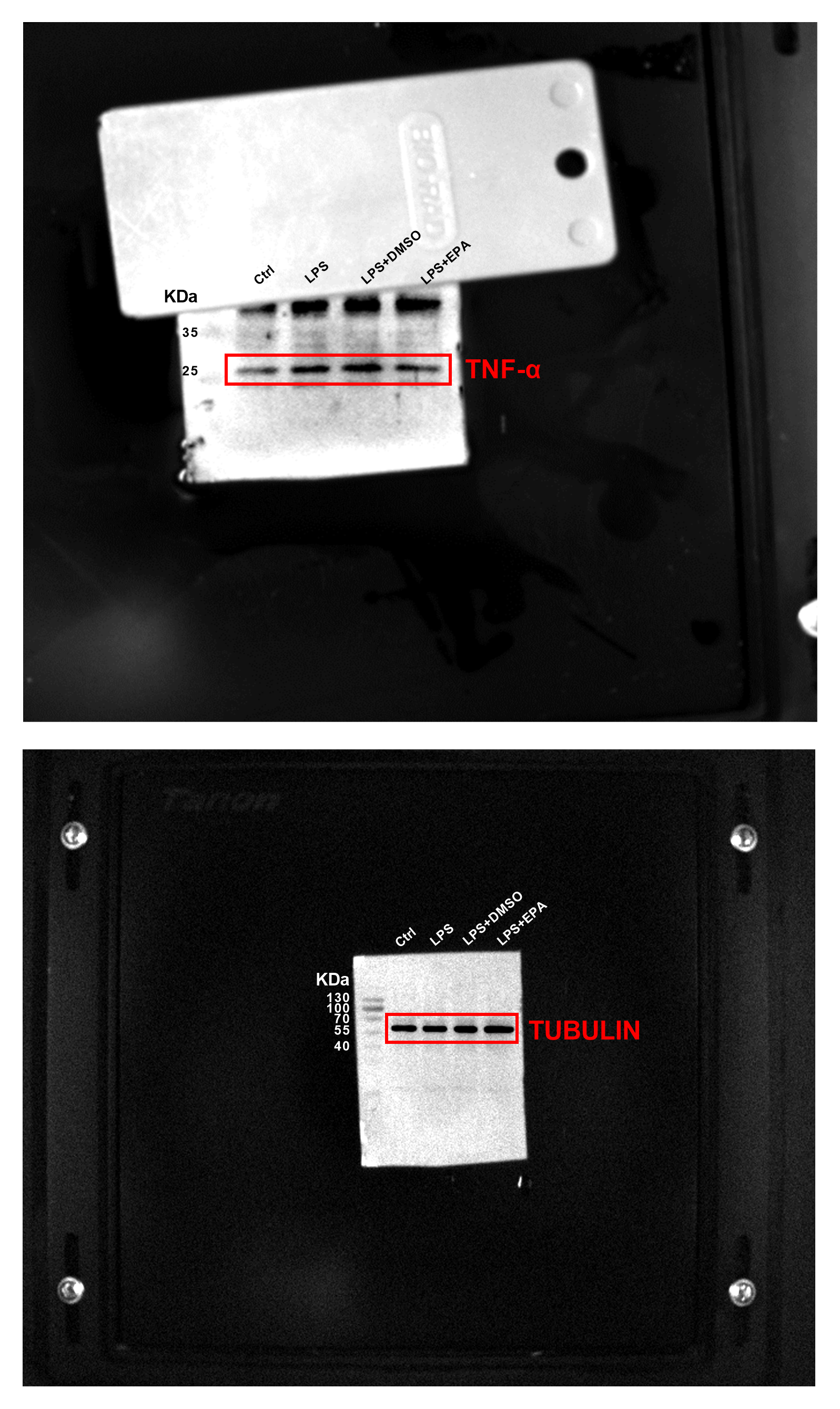

Supplement: Supplementary file 5 — Source data Fig. 3 [file 44319_2024_271_MOESM5_ESM.zip › Figure 3/Fig. 3A TNF-¦Á&TUBULIN.tif]

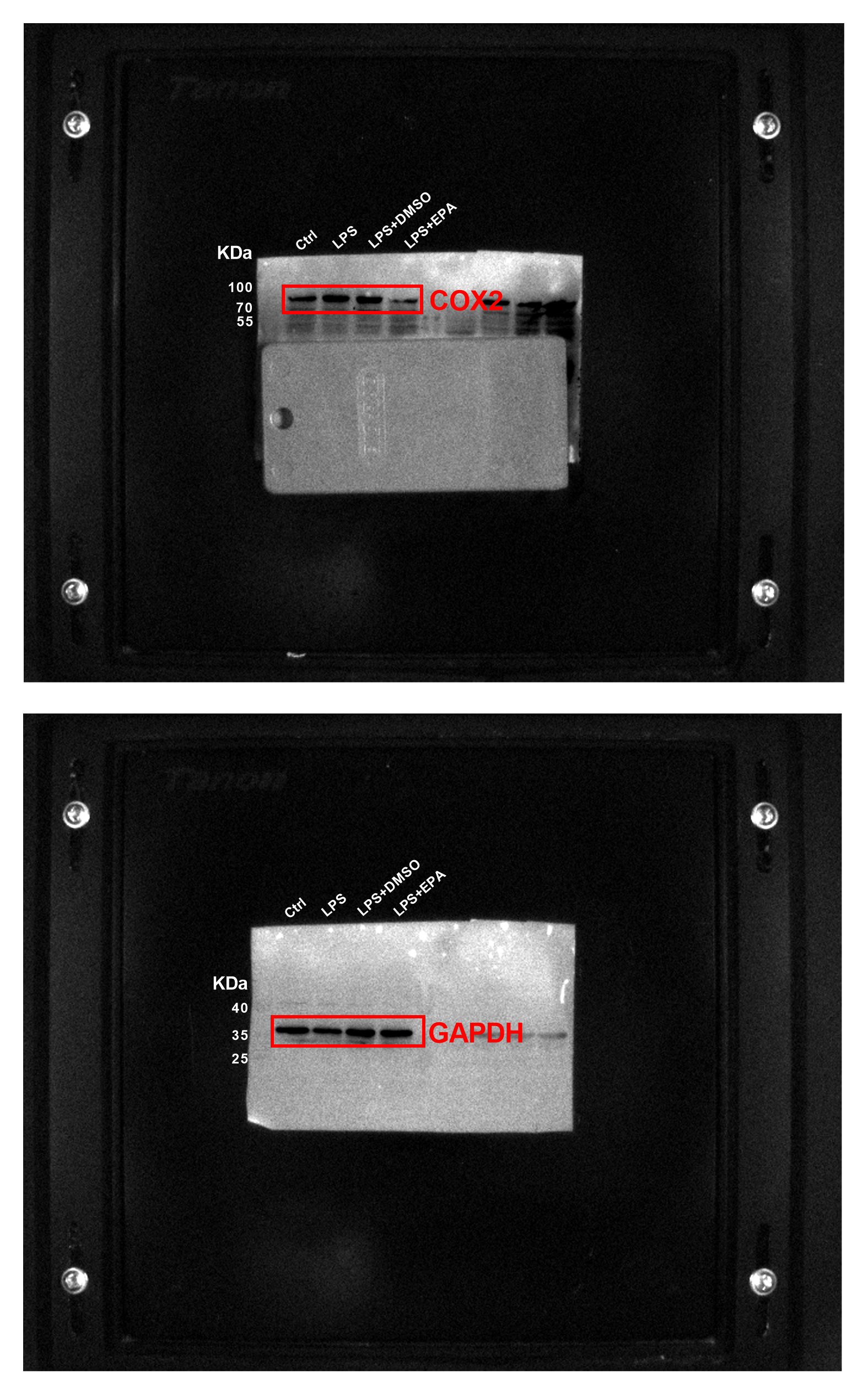

Supplement: Supplementary file 5 — Source data Fig. 3 [file 44319_2024_271_MOESM5_ESM.zip › Figure 3/Fig. 3G COX2&GAPDH.tif]

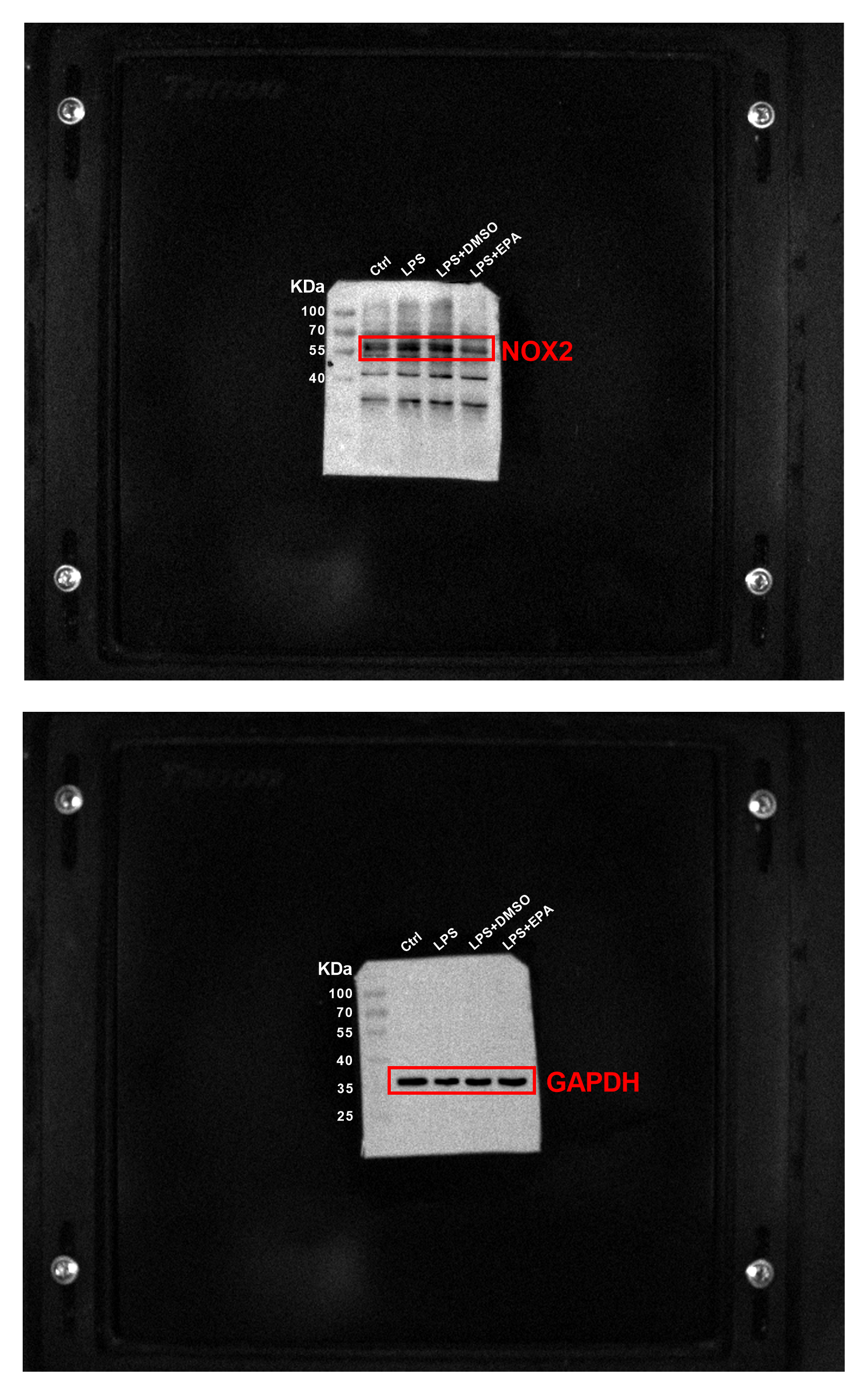

Supplement: Supplementary file 5 — Source data Fig. 3 [file 44319_2024_271_MOESM5_ESM.zip › Figure 3/Fig. 3G NOX2&GAPDH.tif]

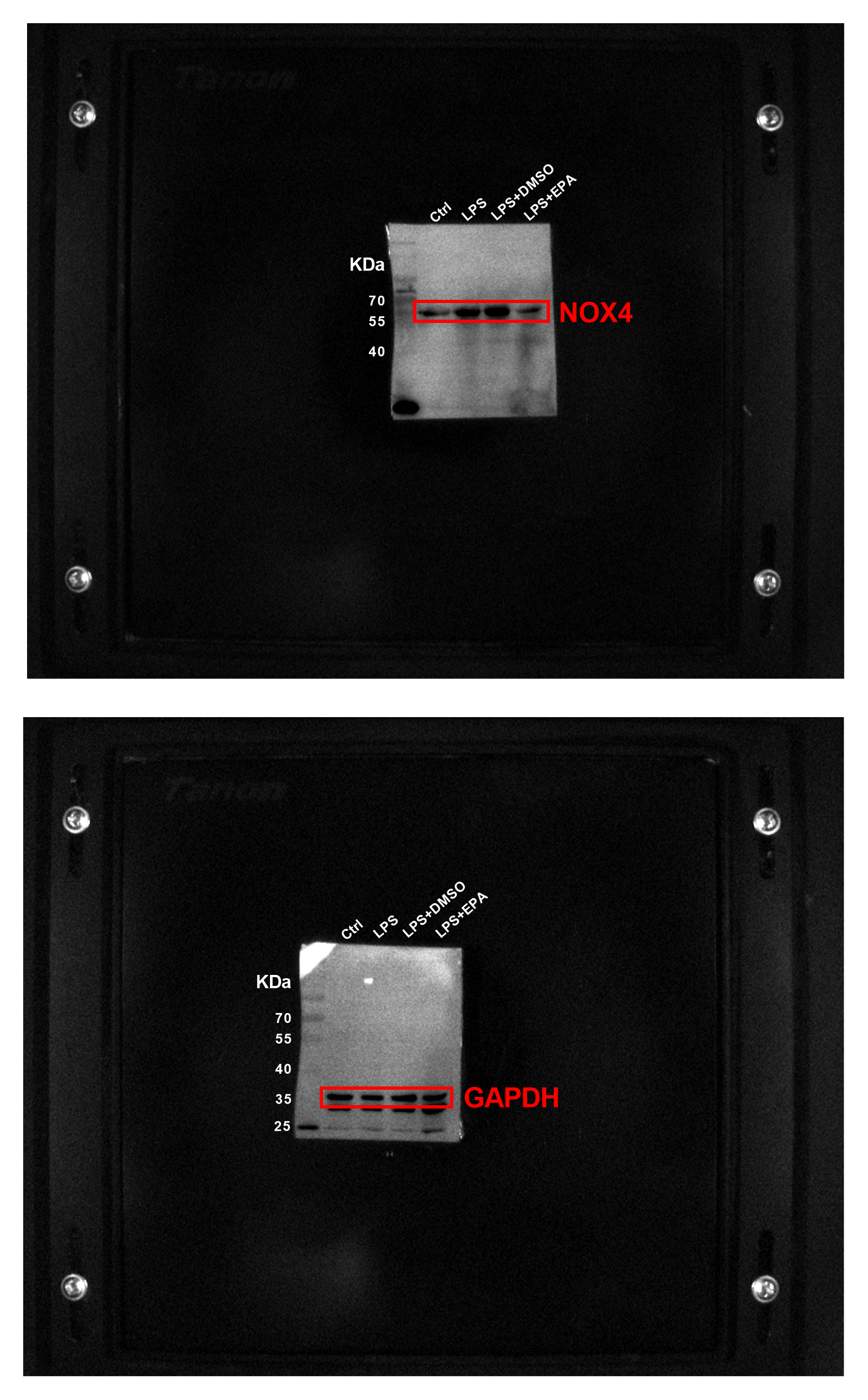

Supplement: Supplementary file 5 — Source data Fig. 3 [file 44319_2024_271_MOESM5_ESM.zip › Figure 3/Fig. 3G NOX4&GAPDH.tif]

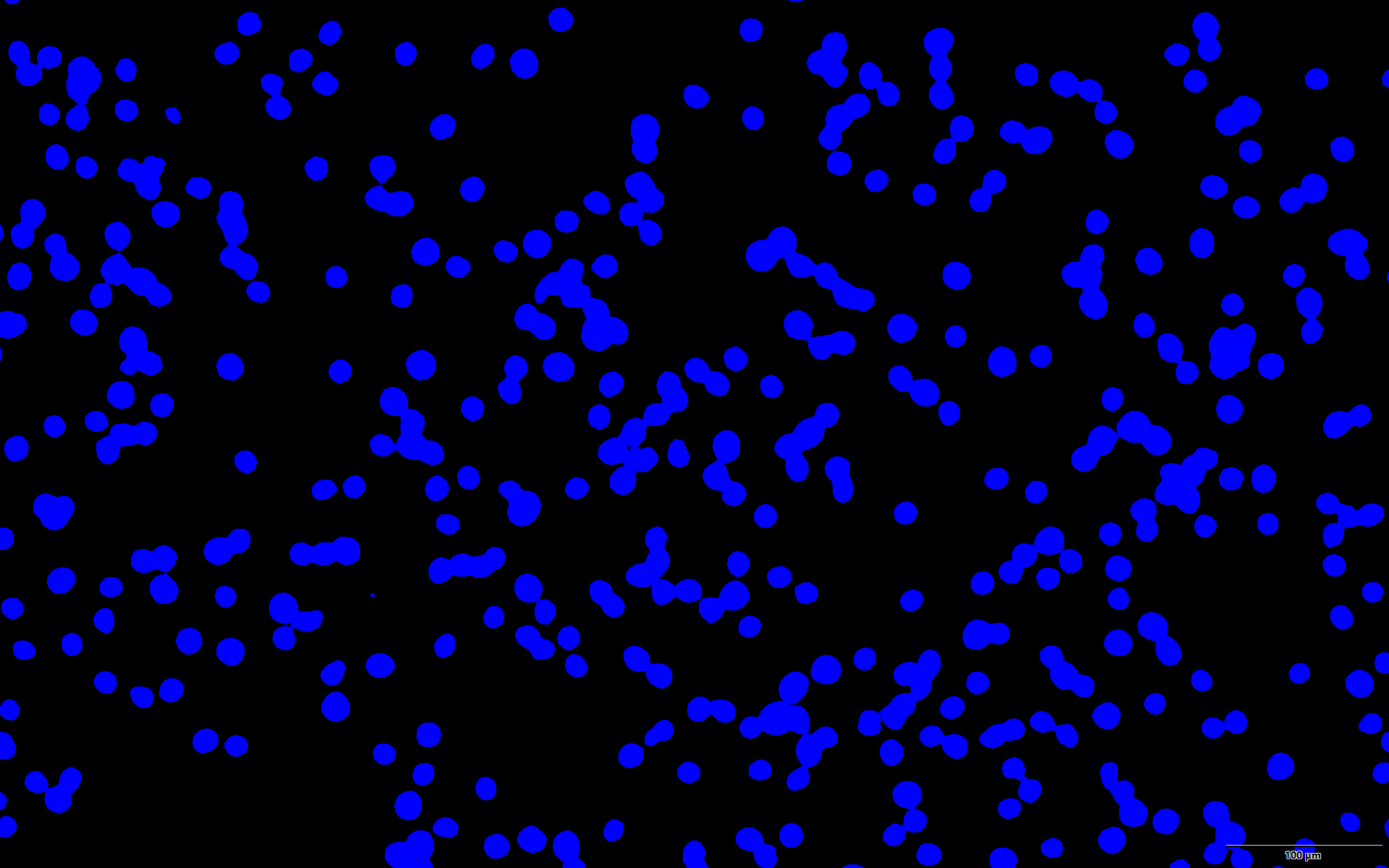

Supplement: Supplementary file 5 — Source data Fig. 3 [file 44319_2024_271_MOESM5_ESM.zip › Figure 3/Fig. 3J Ctrl-DAPI.tif]

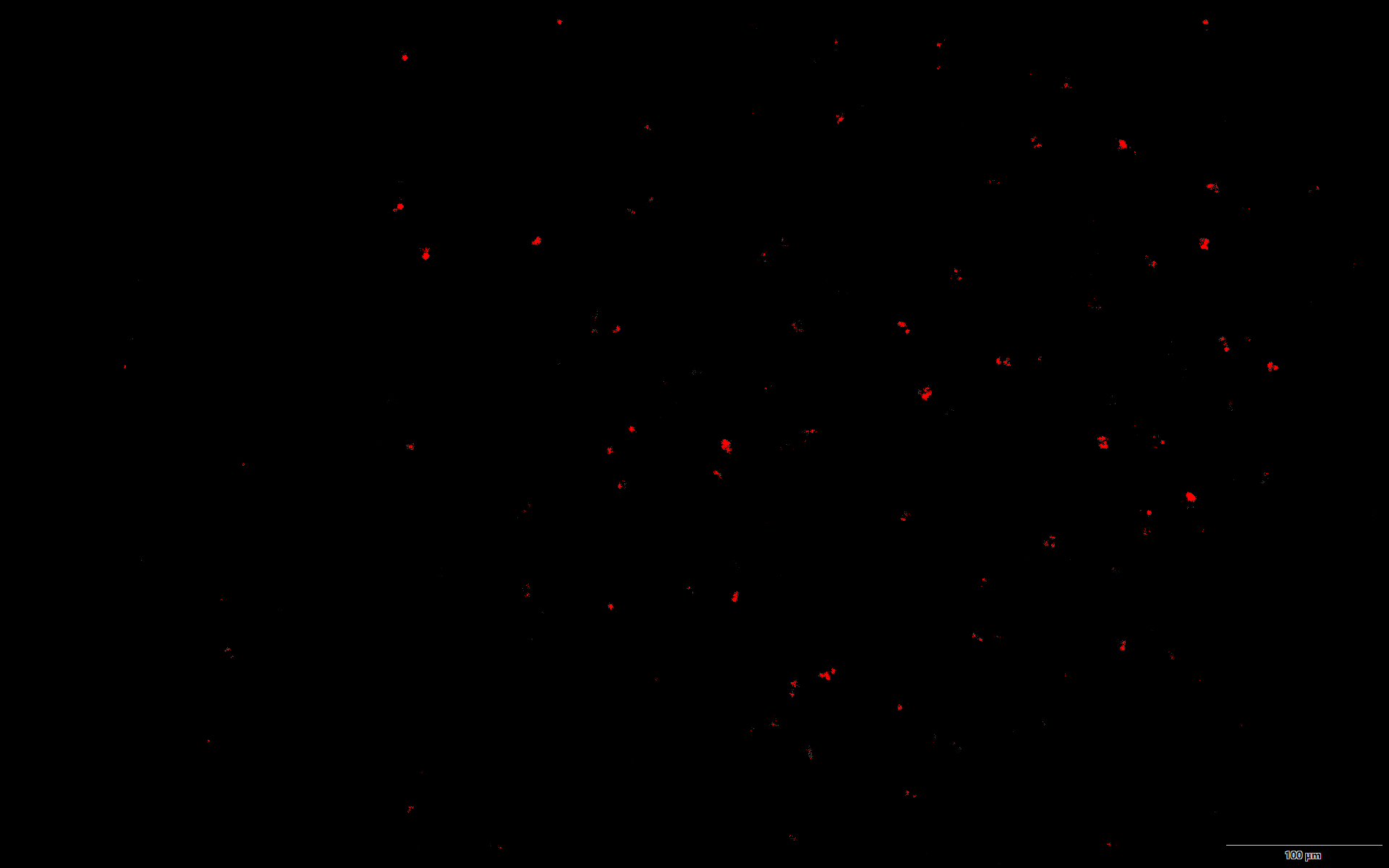

Supplement: Supplementary file 5 — Source data Fig. 3 [file 44319_2024_271_MOESM5_ESM.zip › Figure 3/Fig. 3J Ctrl-DHE.tif]

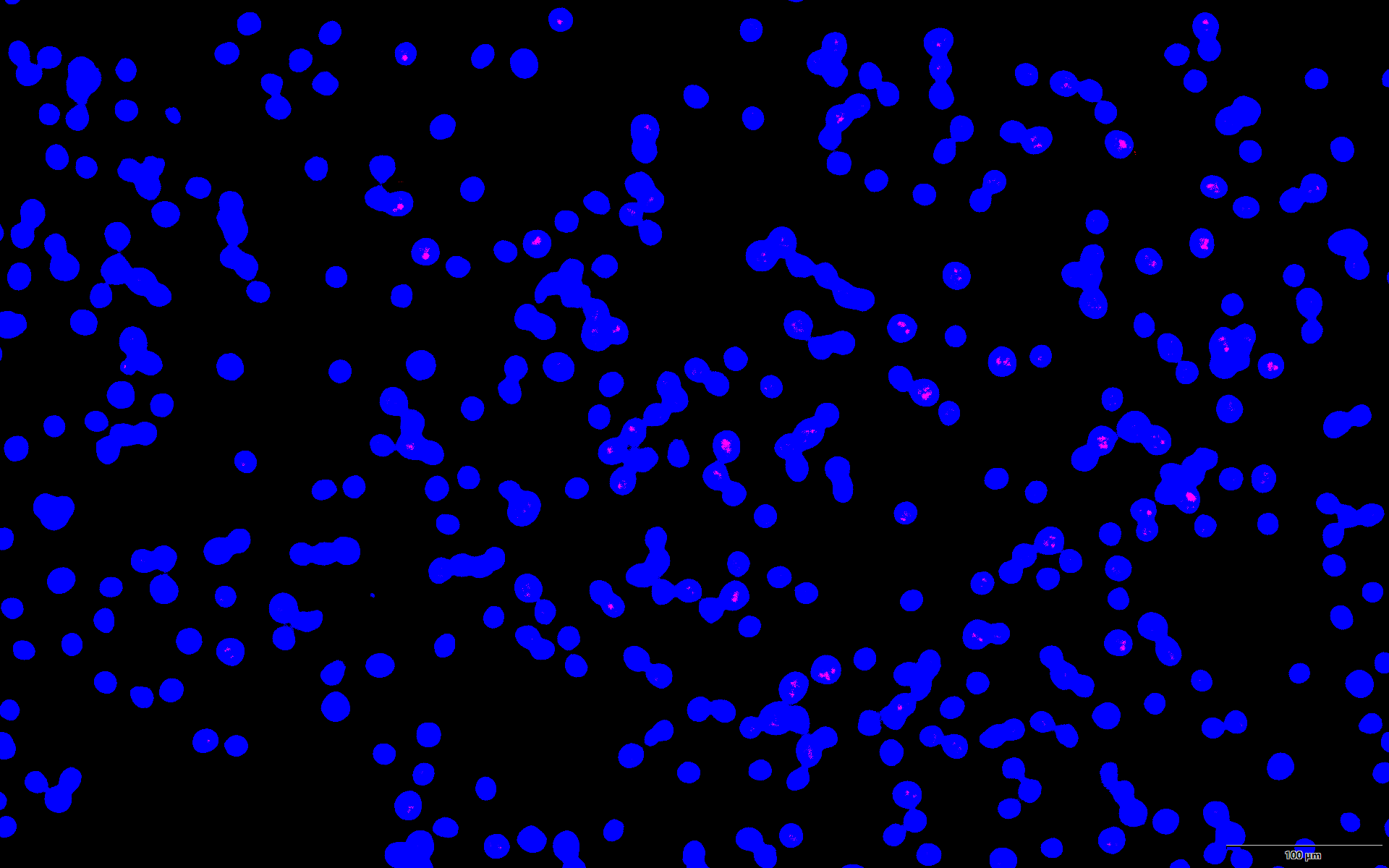

Supplement: Supplementary file 5 — Source data Fig. 3 [file 44319_2024_271_MOESM5_ESM.zip › Figure 3/Fig. 3J Ctrl-Merge.tif]

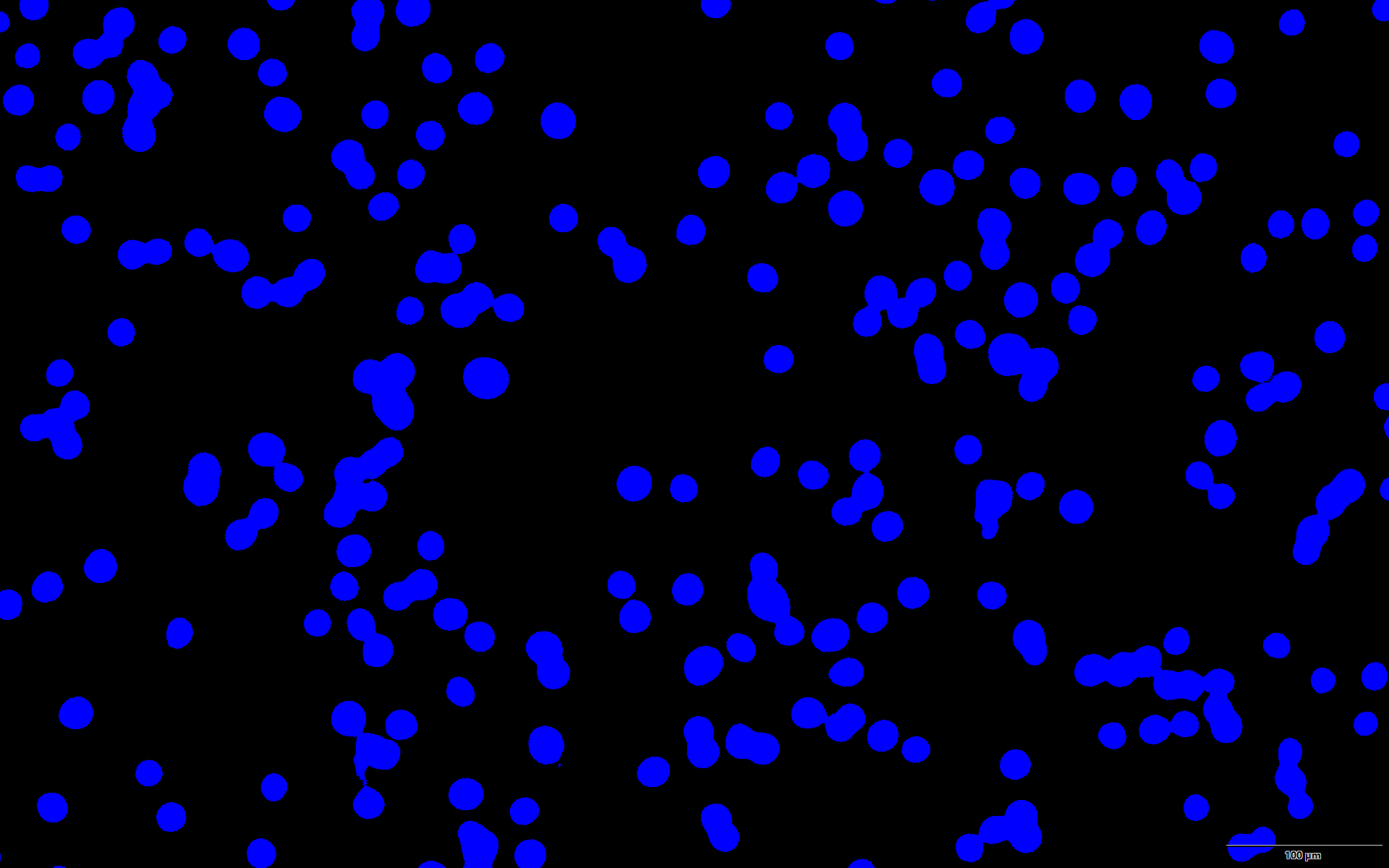

Supplement: Supplementary file 5 — Source data Fig. 3 [file 44319_2024_271_MOESM5_ESM.zip › Figure 3/Fig. 3J LPS+DMSO-DAPI.tif]

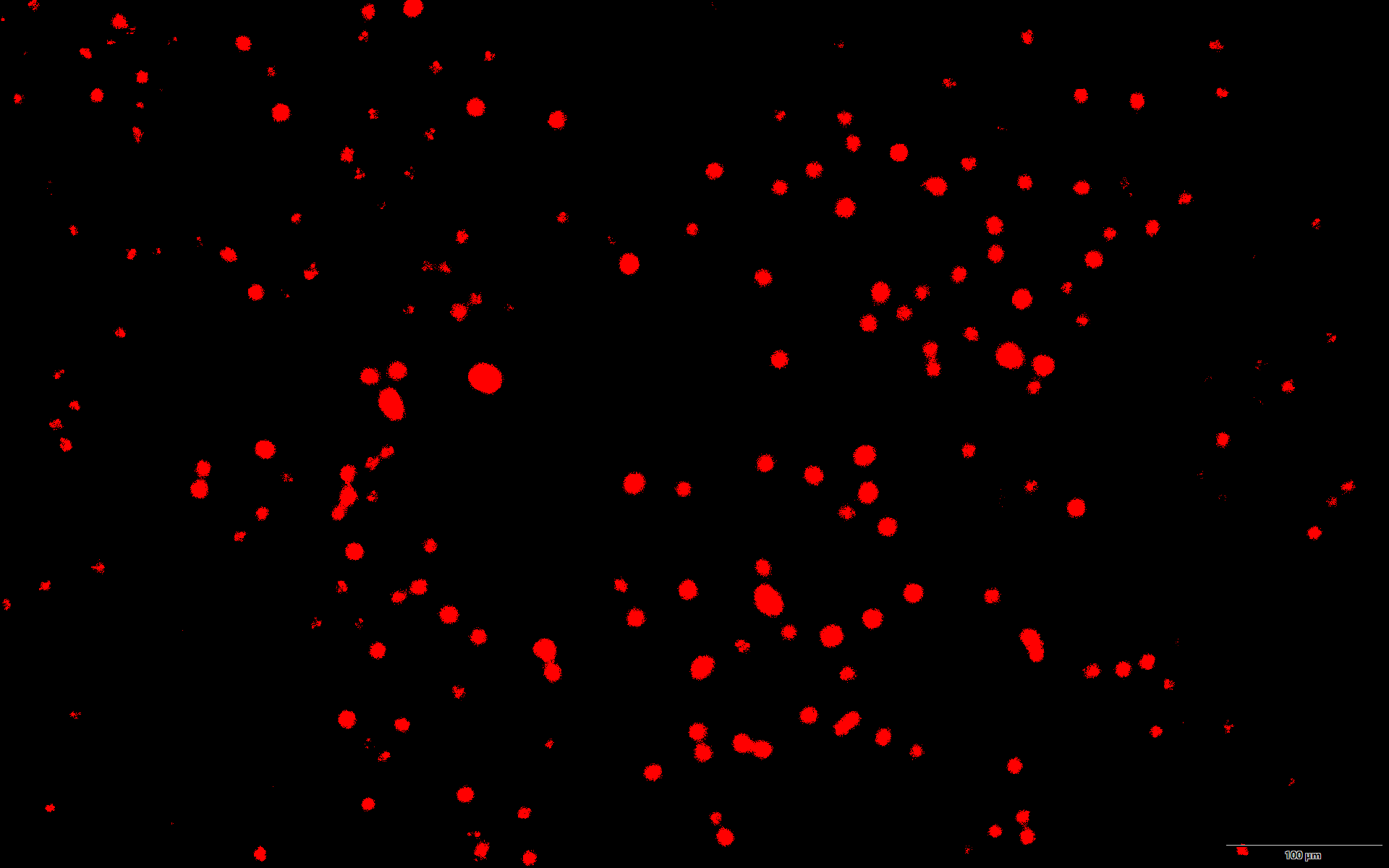

Supplement: Supplementary file 5 — Source data Fig. 3 [file 44319_2024_271_MOESM5_ESM.zip › Figure 3/Fig. 3J LPS+DMSO-DHE.tif]

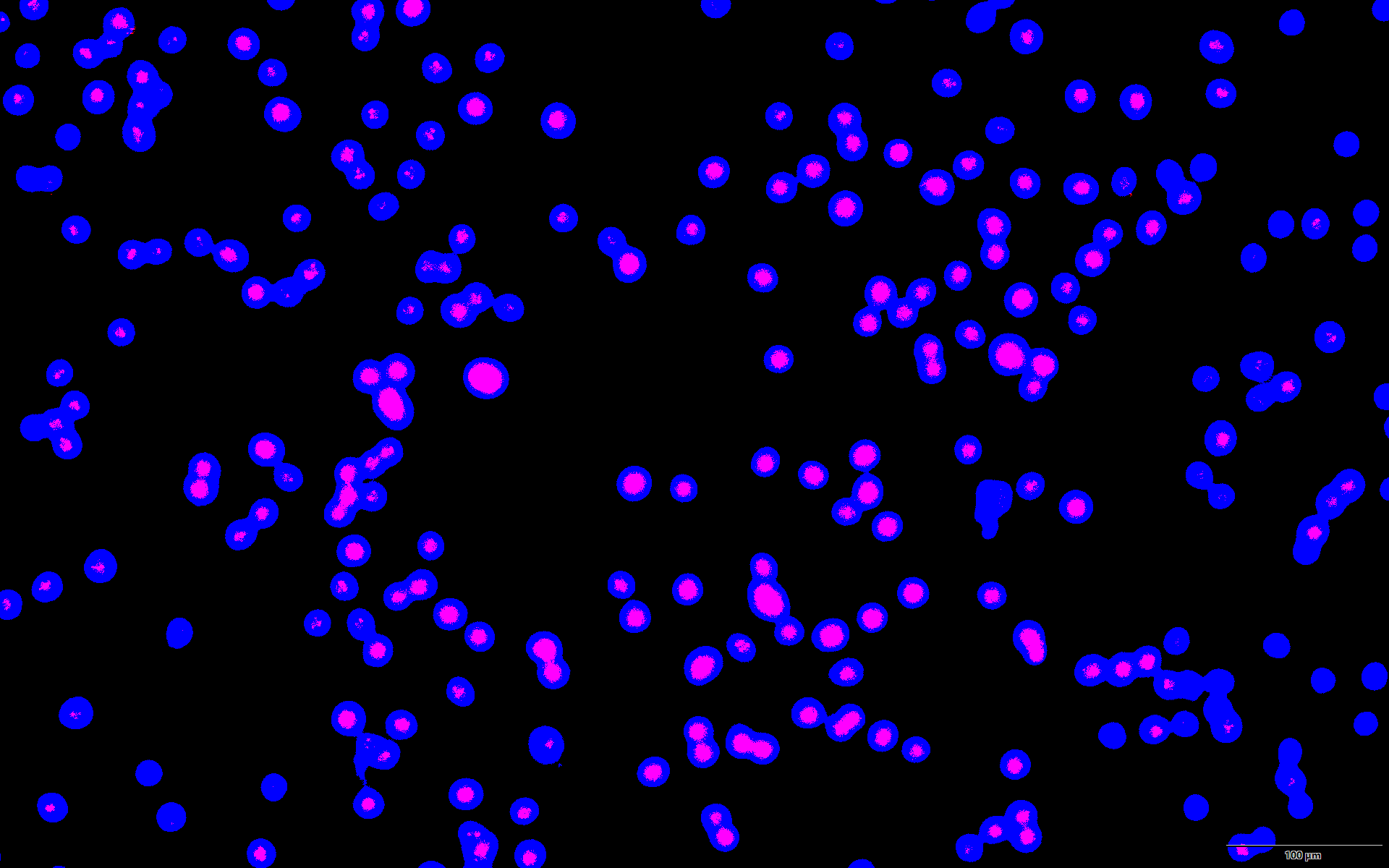

Supplement: Supplementary file 5 — Source data Fig. 3 [file 44319_2024_271_MOESM5_ESM.zip › Figure 3/Fig. 3J LPS+DMSO-Merge.tif]

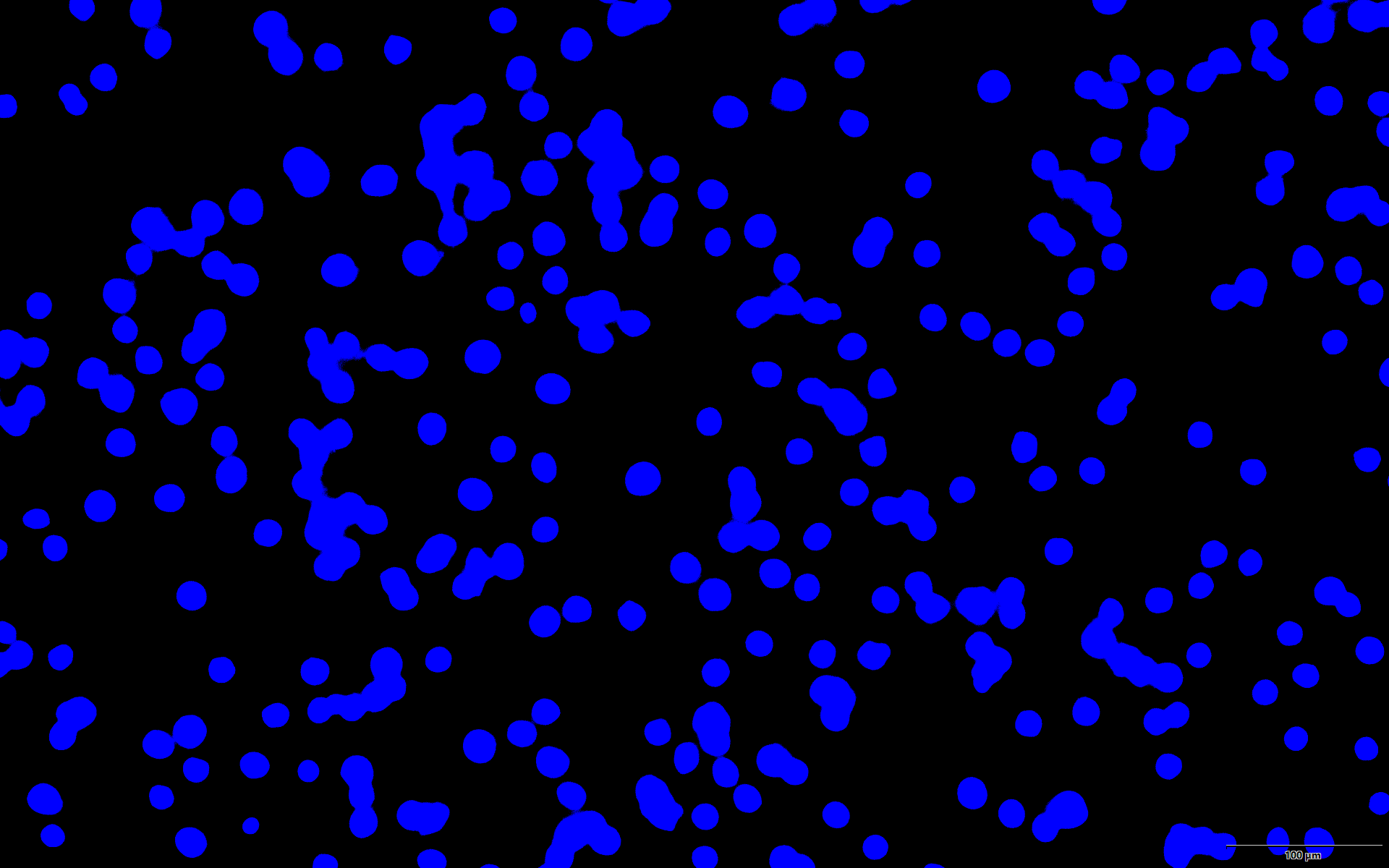

Supplement: Supplementary file 5 — Source data Fig. 3 [file 44319_2024_271_MOESM5_ESM.zip › Figure 3/Fig. 3J LPS+EPA-DAPI.tif]

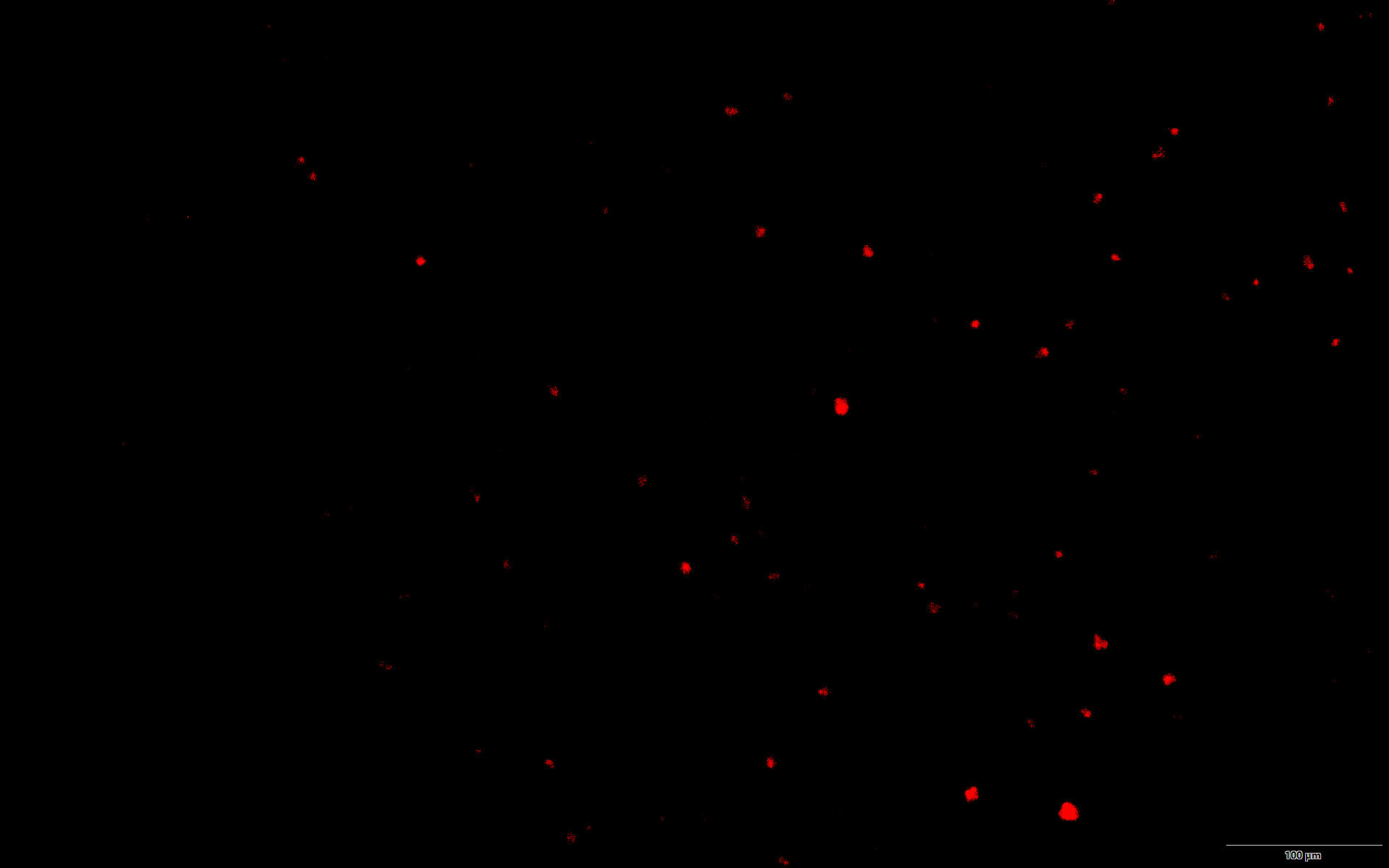

Supplement: Supplementary file 5 — Source data Fig. 3 [file 44319_2024_271_MOESM5_ESM.zip › Figure 3/Fig. 3J LPS+EPA-DHE.tif]

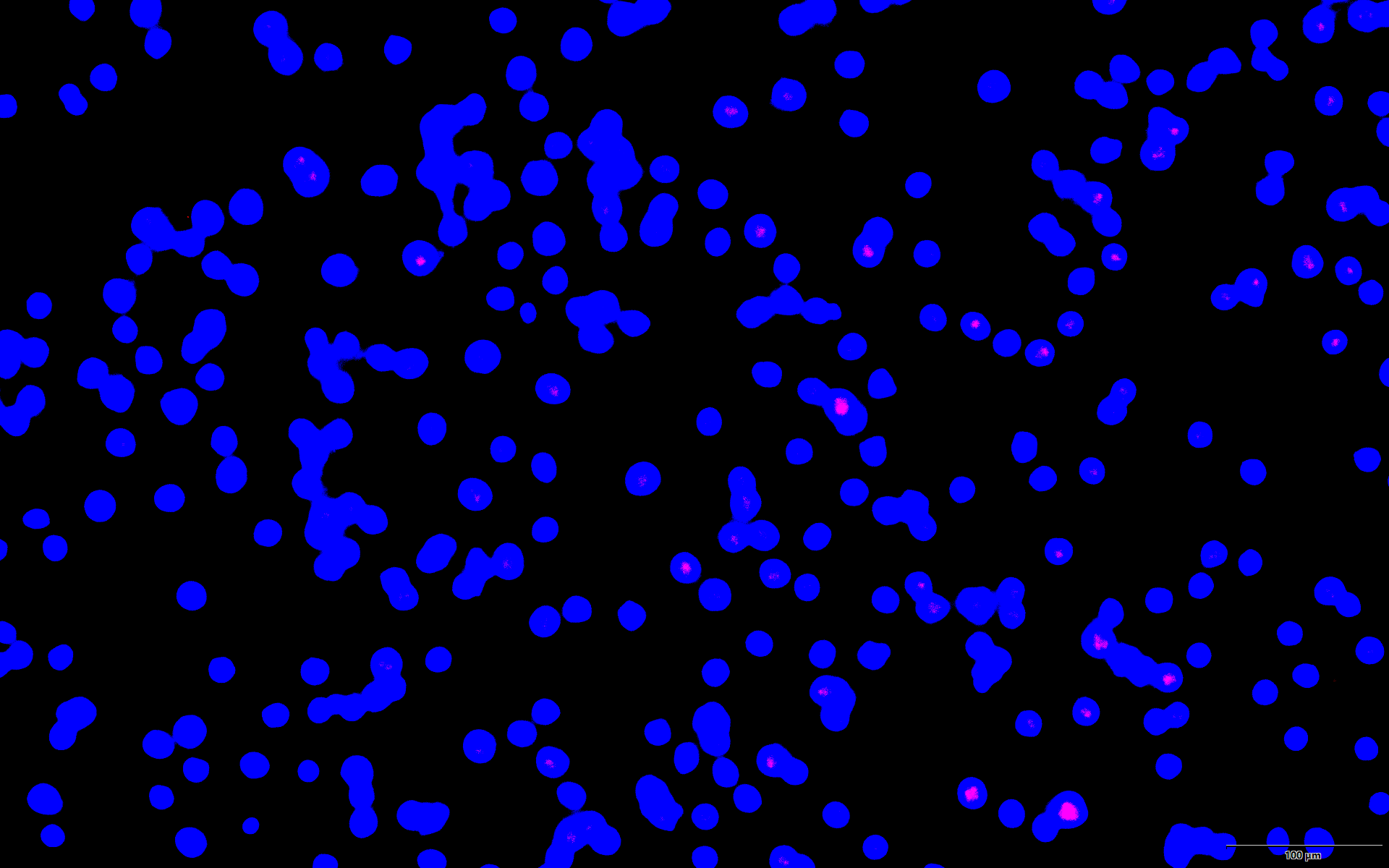

Supplement: Supplementary file 5 — Source data Fig. 3 [file 44319_2024_271_MOESM5_ESM.zip › Figure 3/Fig. 3J LPS+EPA-Merge.tif]

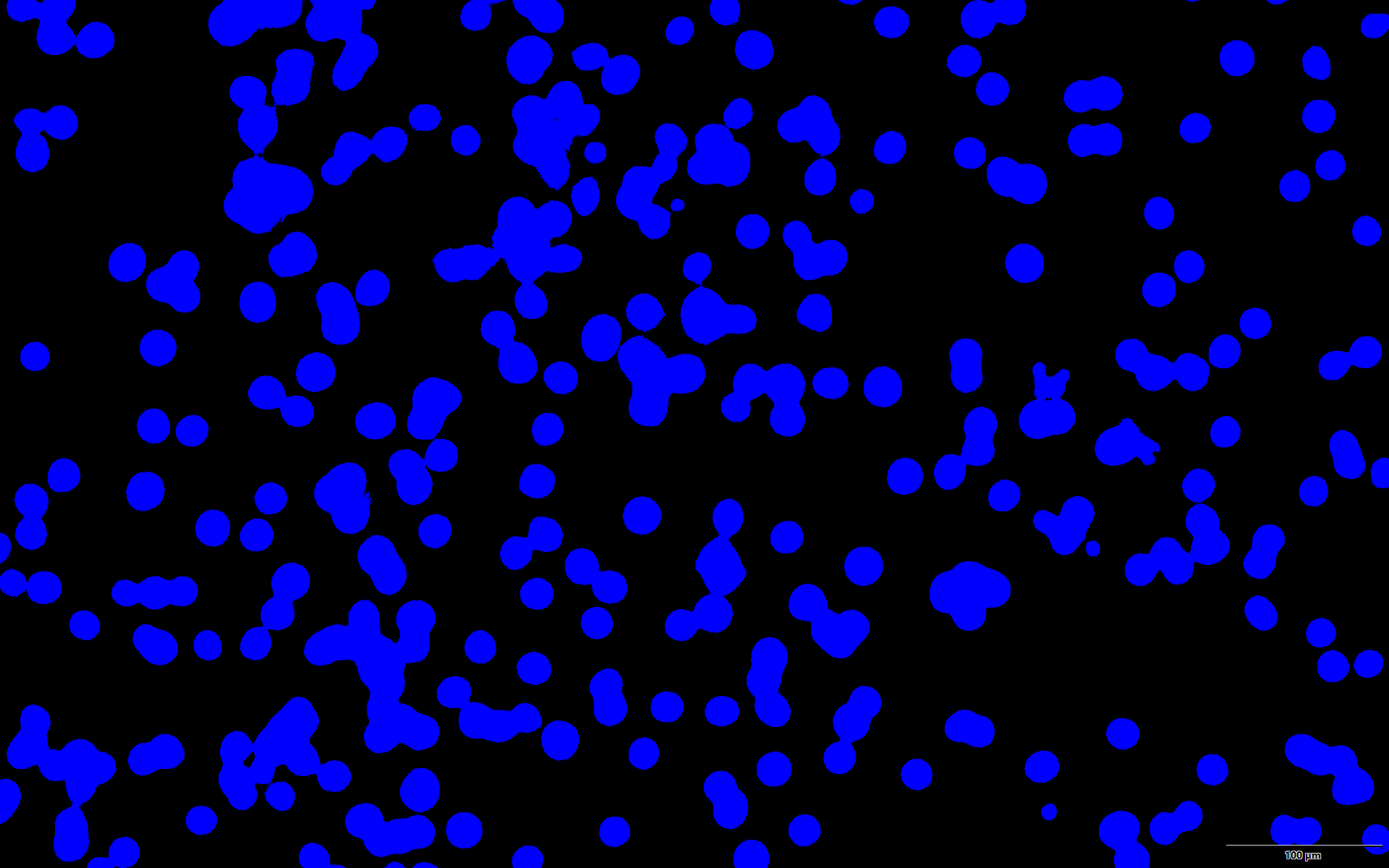

Supplement: Supplementary file 5 — Source data Fig. 3 [file 44319_2024_271_MOESM5_ESM.zip › Figure 3/Fig. 3J LPS-DAPI.tif]

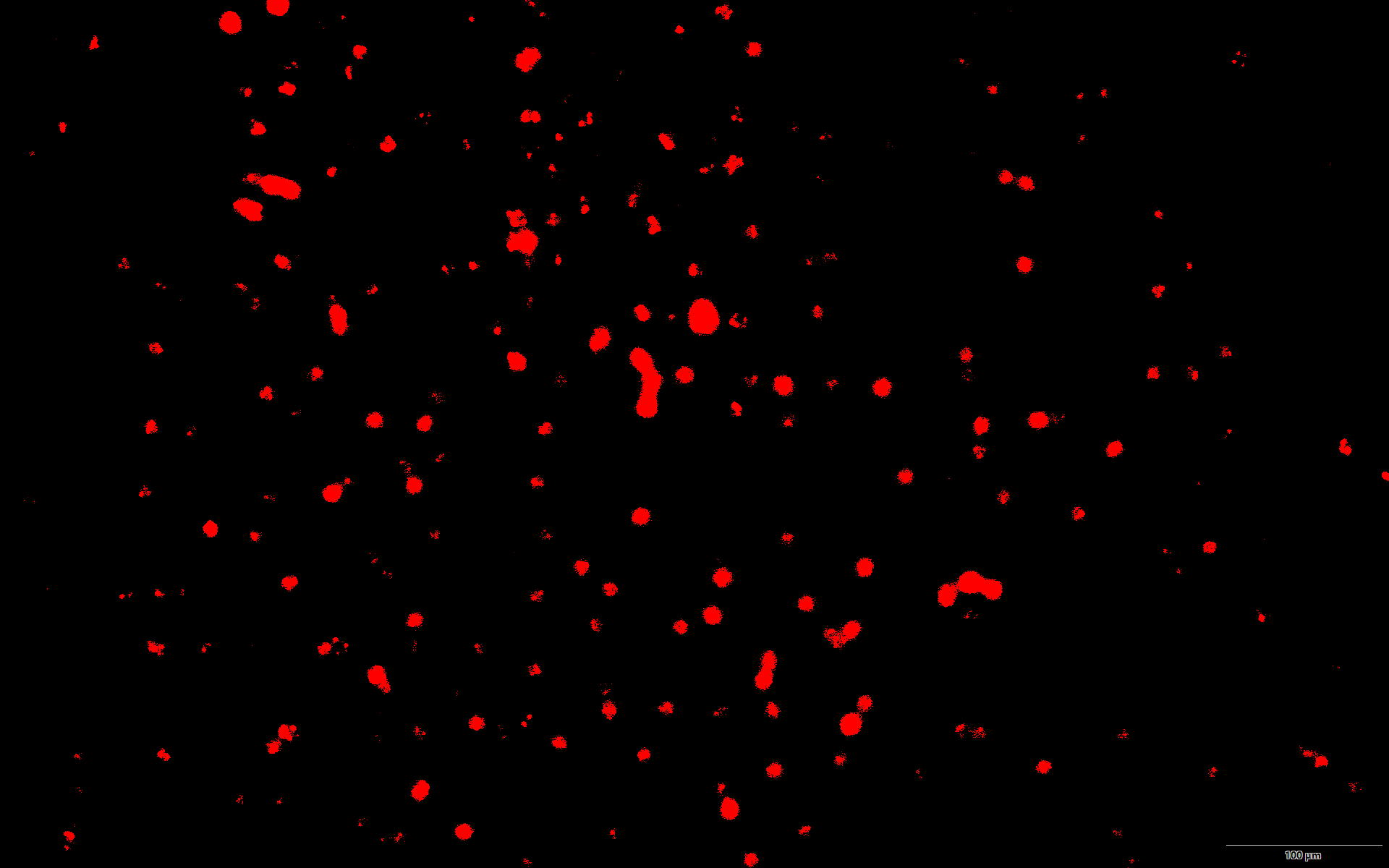

Supplement: Supplementary file 5 — Source data Fig. 3 [file 44319_2024_271_MOESM5_ESM.zip › Figure 3/Fig. 3J LPS-DHE.tif]

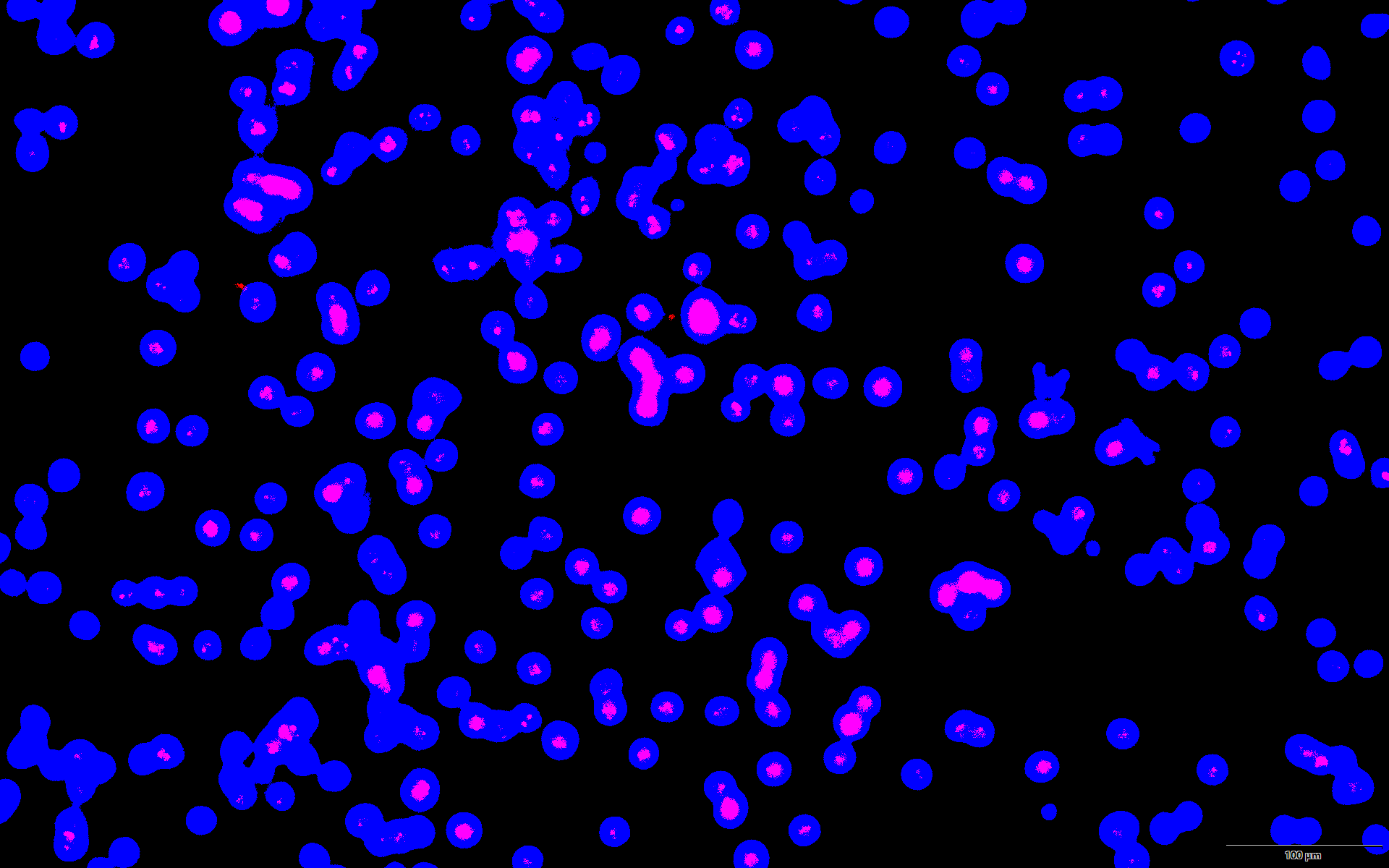

Supplement: Supplementary file 5 — Source data Fig. 3 [file 44319_2024_271_MOESM5_ESM.zip › Figure 3/Fig. 3J LPS-Merge.tif]

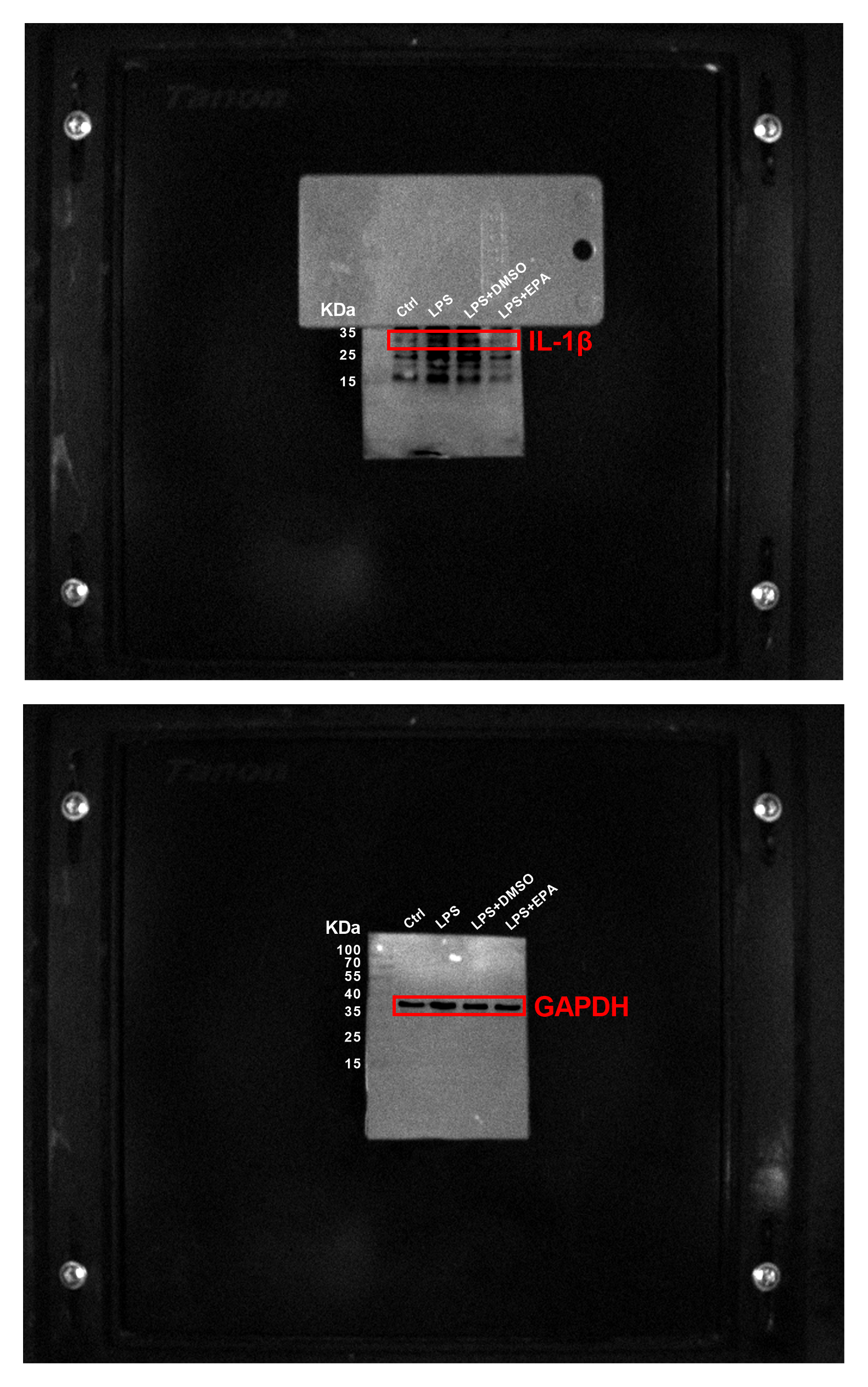

Supplement: Supplementary file 6 — Source data Fig. 4 [file 44319_2024_271_MOESM6_ESM.zip › Figure 4/Fig. 4A IL-1¦Â&GAPDH.tif]

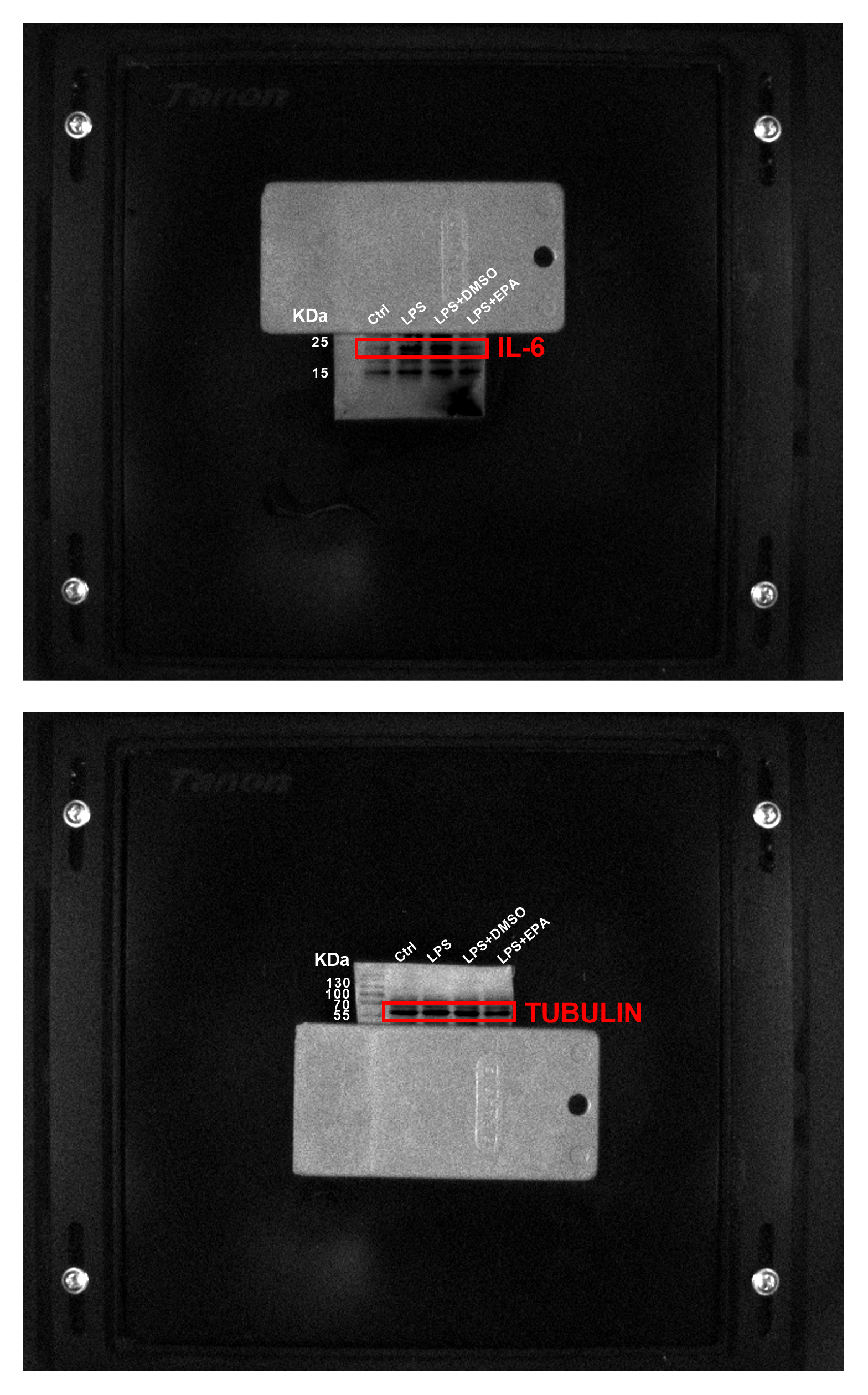

Supplement: Supplementary file 6 — Source data Fig. 4 [file 44319_2024_271_MOESM6_ESM.zip › Figure 4/Fig. 4A IL-6&TUBULIN.tif]

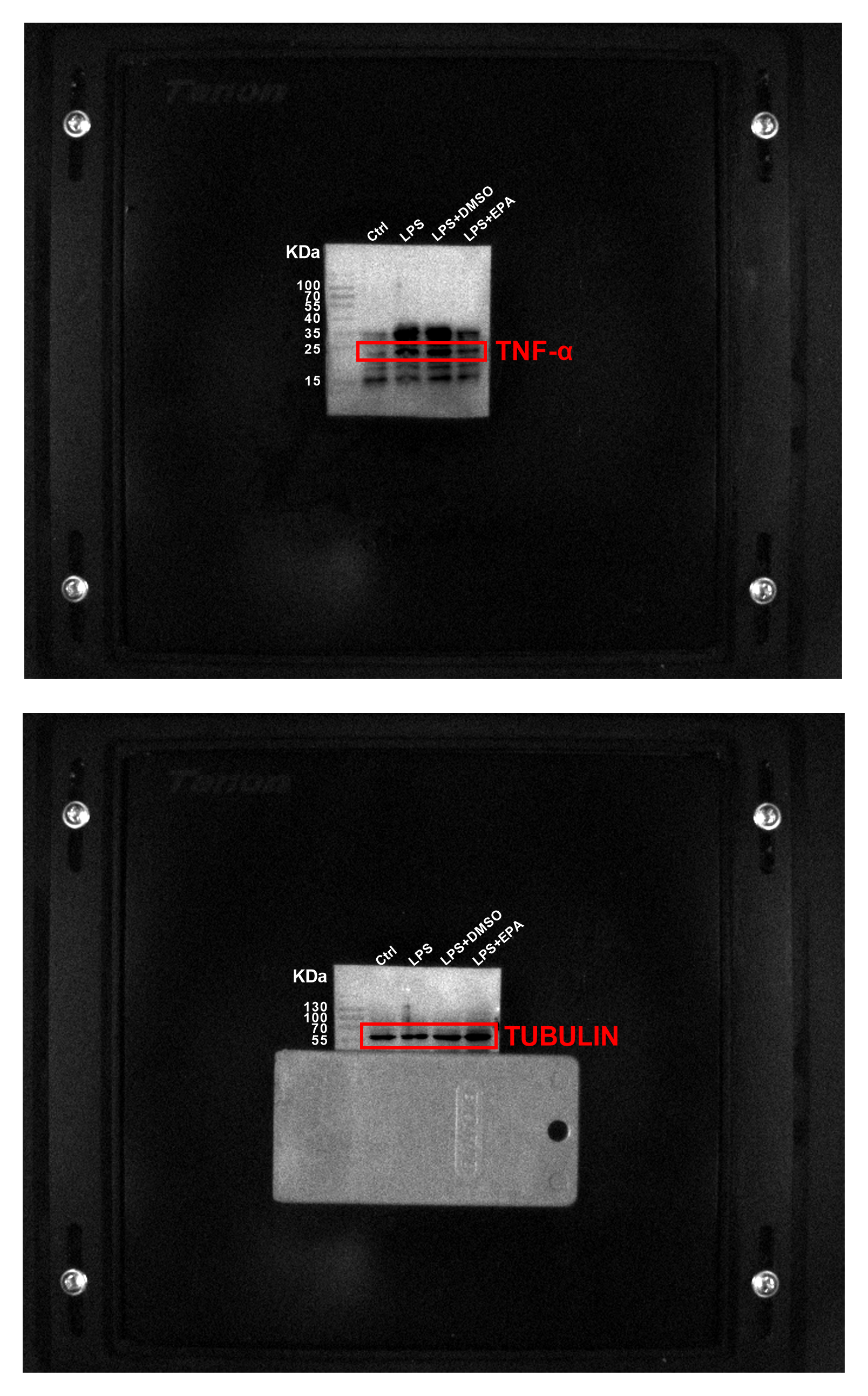

Supplement: Supplementary file 6 — Source data Fig. 4 [file 44319_2024_271_MOESM6_ESM.zip › Figure 4/Fig. 4A TNF-¦Á&TUBULIN.tif]

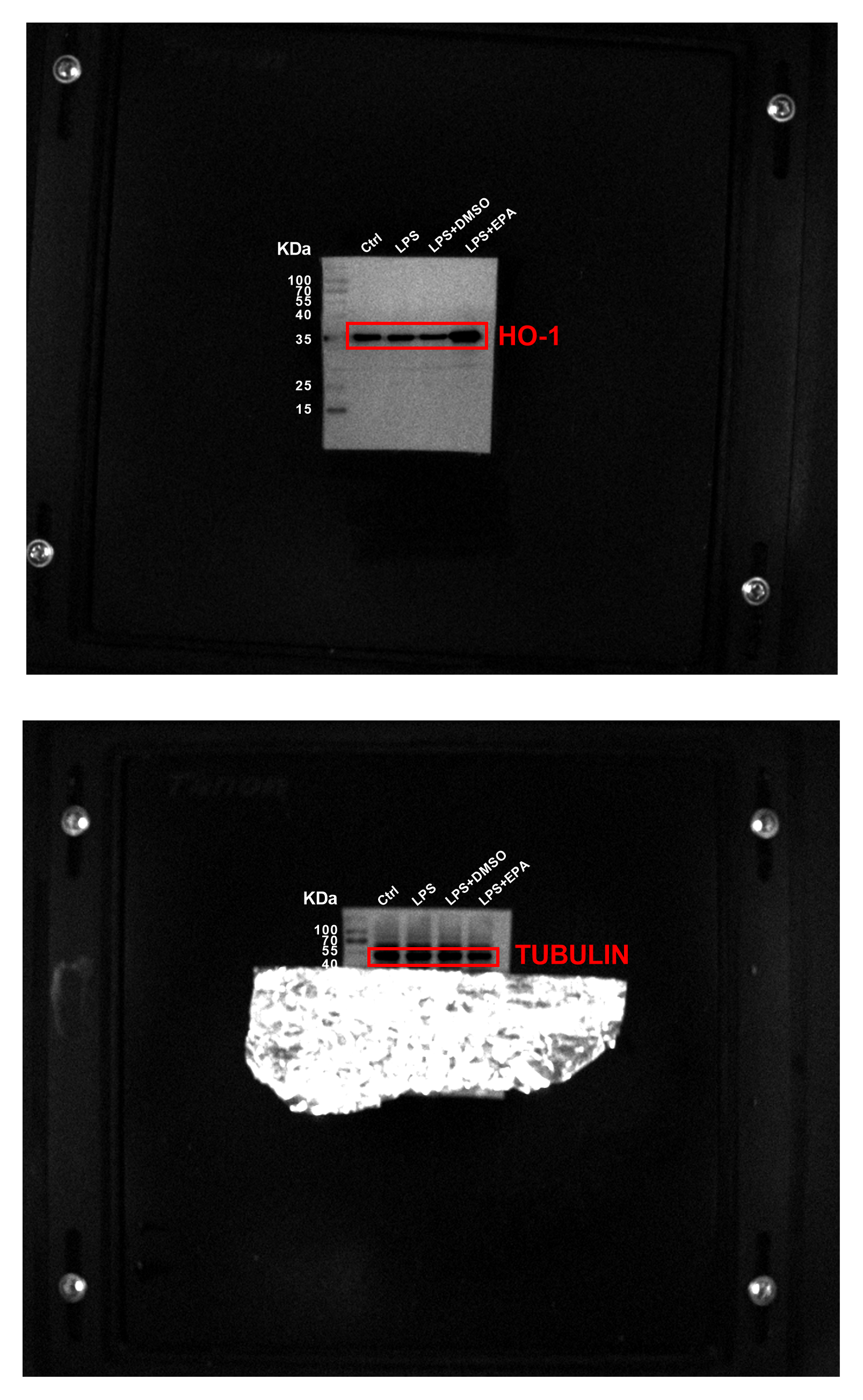

Supplement: Supplementary file 7 — Source data Fig. 5 [file 44319_2024_271_MOESM7_ESM.zip › Figure 5/Fig. 5A HO-1&TUBULIN.tif]

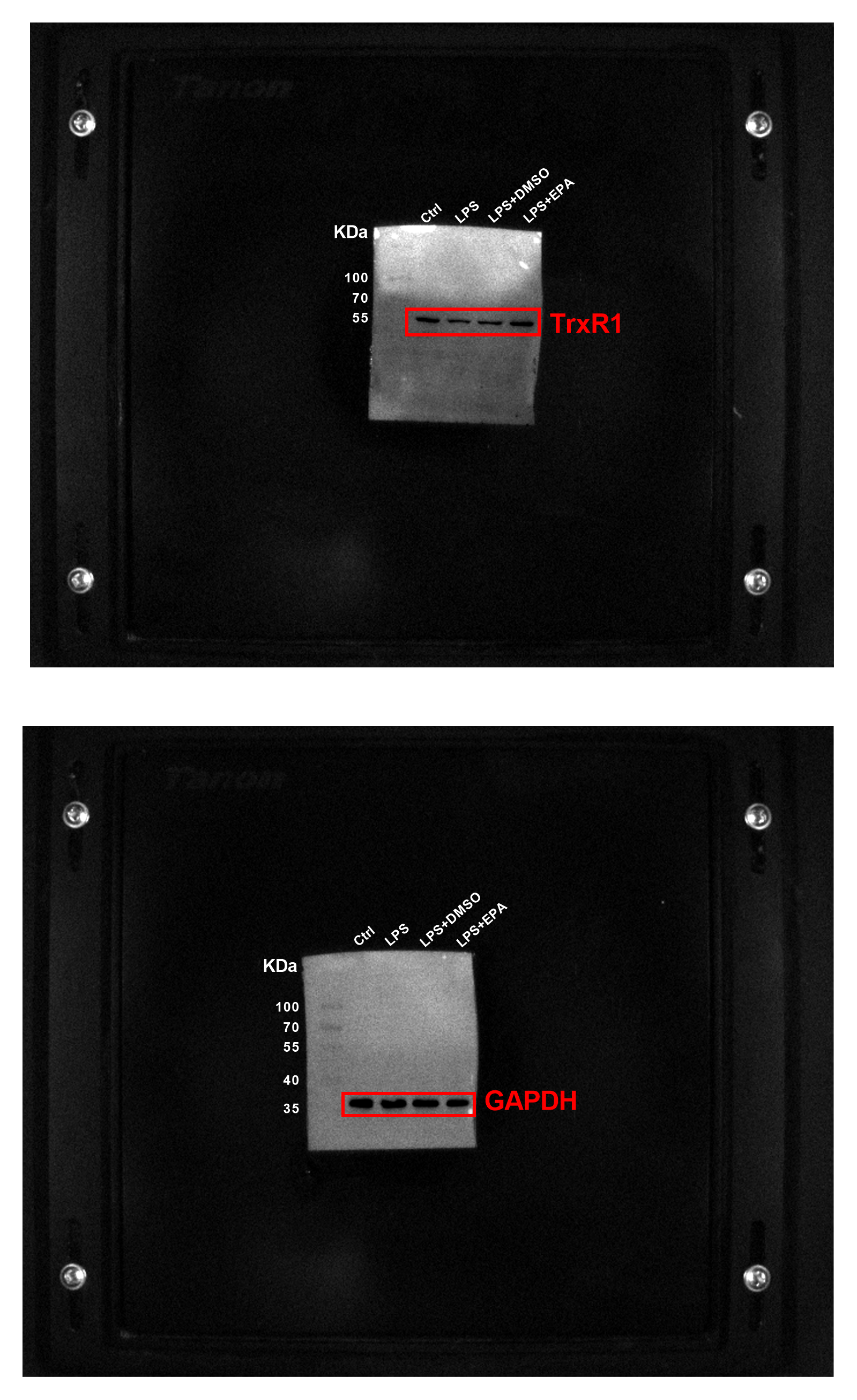

Supplement: Supplementary file 7 — Source data Fig. 5 [file 44319_2024_271_MOESM7_ESM.zip › Figure 5/Fig. 5A TrxR1&GAPDH.tif]

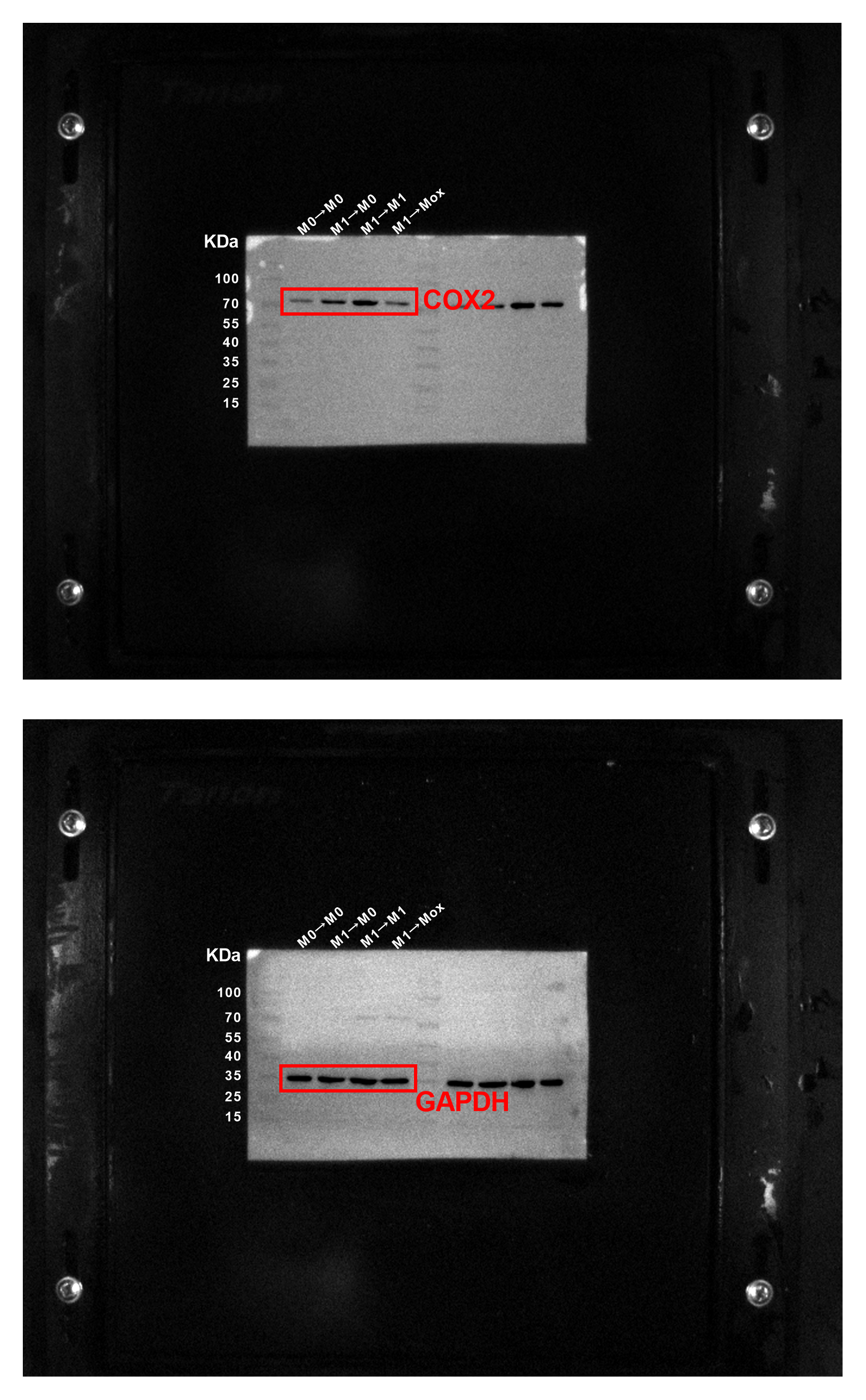

Supplement: Supplementary file 7 — Source data Fig. 5 [file 44319_2024_271_MOESM7_ESM.zip › Figure 5/Fig. 5G COX2&GAPDH.tif]

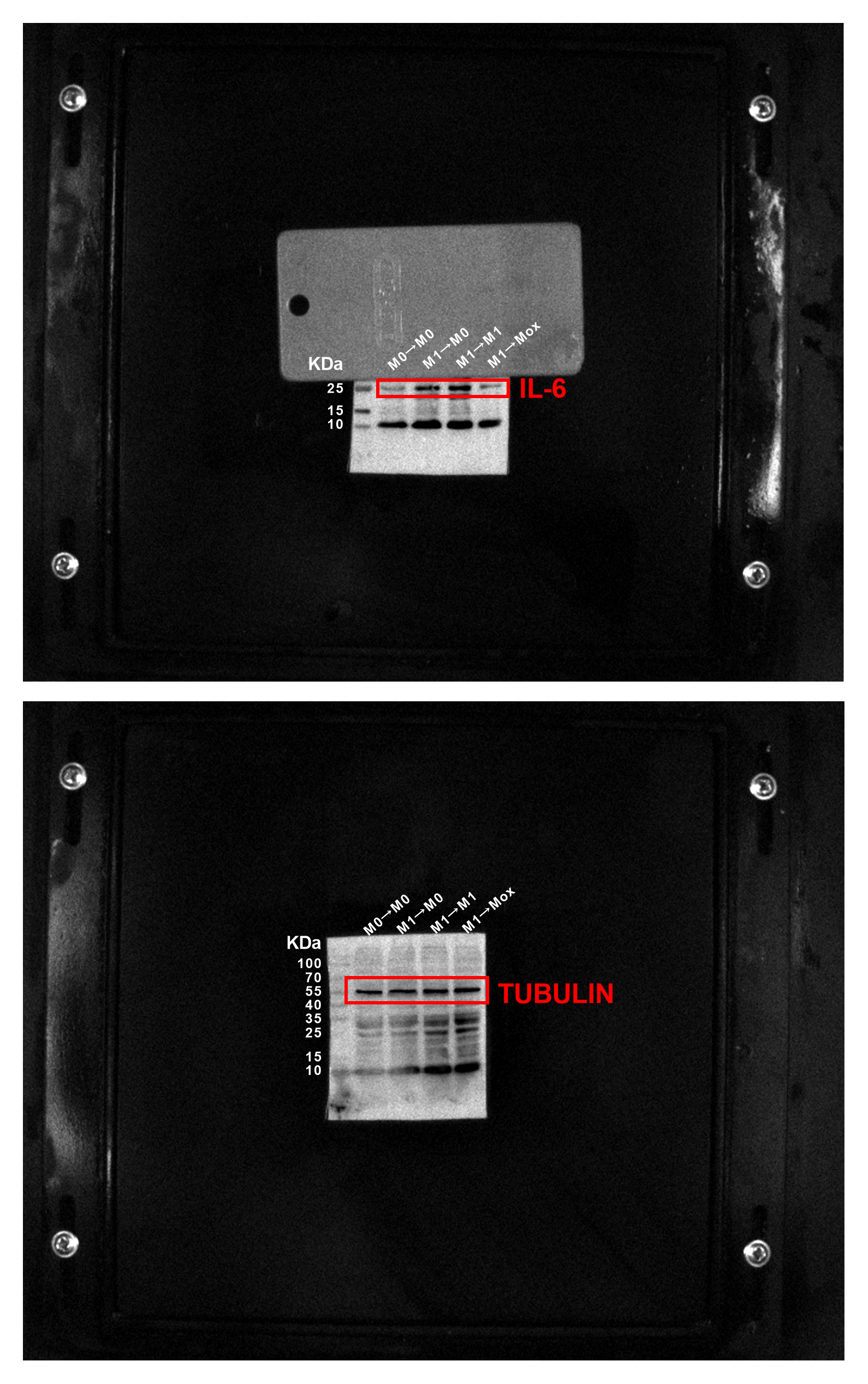

Supplement: Supplementary file 7 — Source data Fig. 5 [file 44319_2024_271_MOESM7_ESM.zip › Figure 5/Fig. 5G IL-6&TUBULIN.tif]

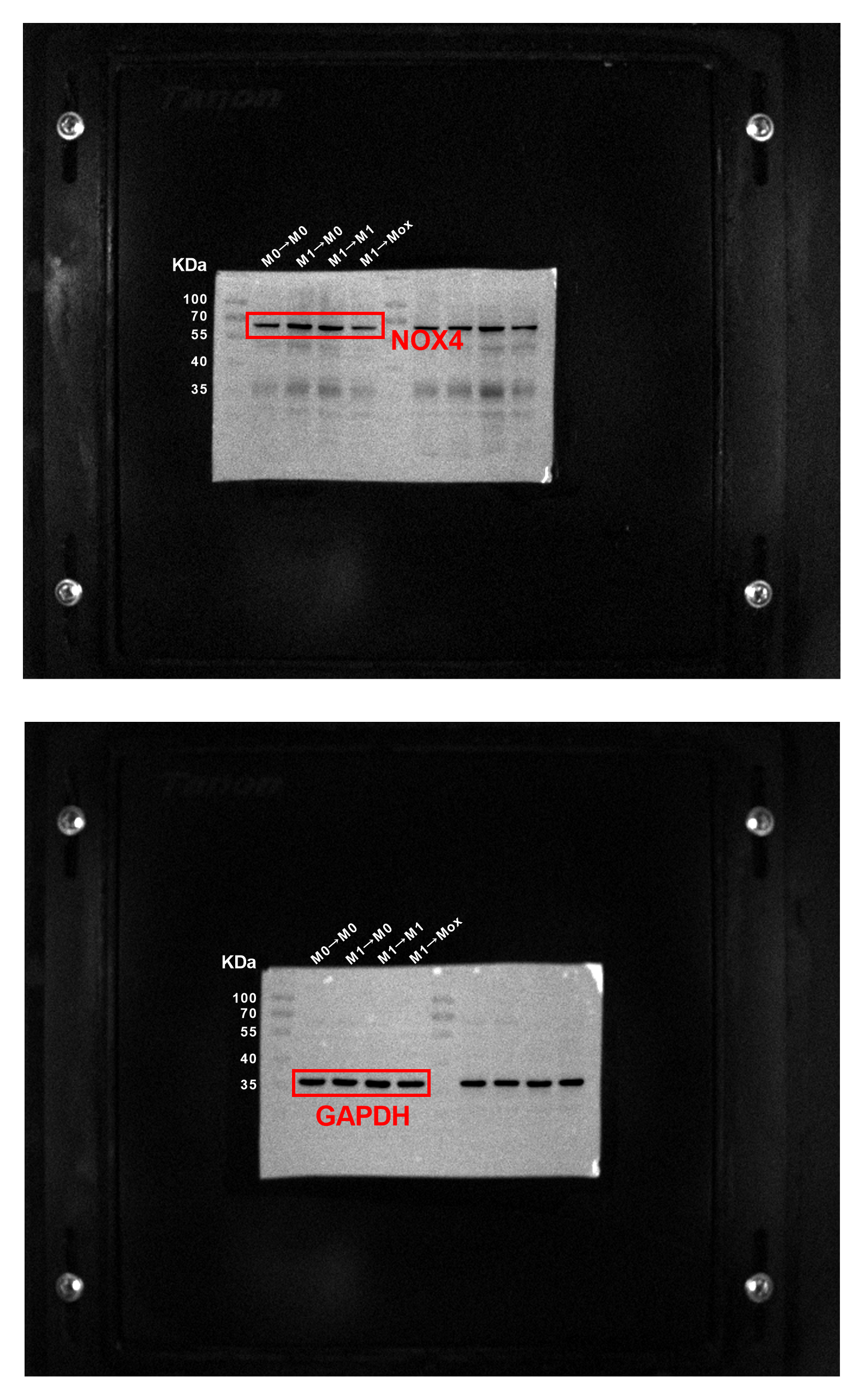

Supplement: Supplementary file 7 — Source data Fig. 5 [file 44319_2024_271_MOESM7_ESM.zip › Figure 5/Fig. 5G NOX4&GAPDH.tif]

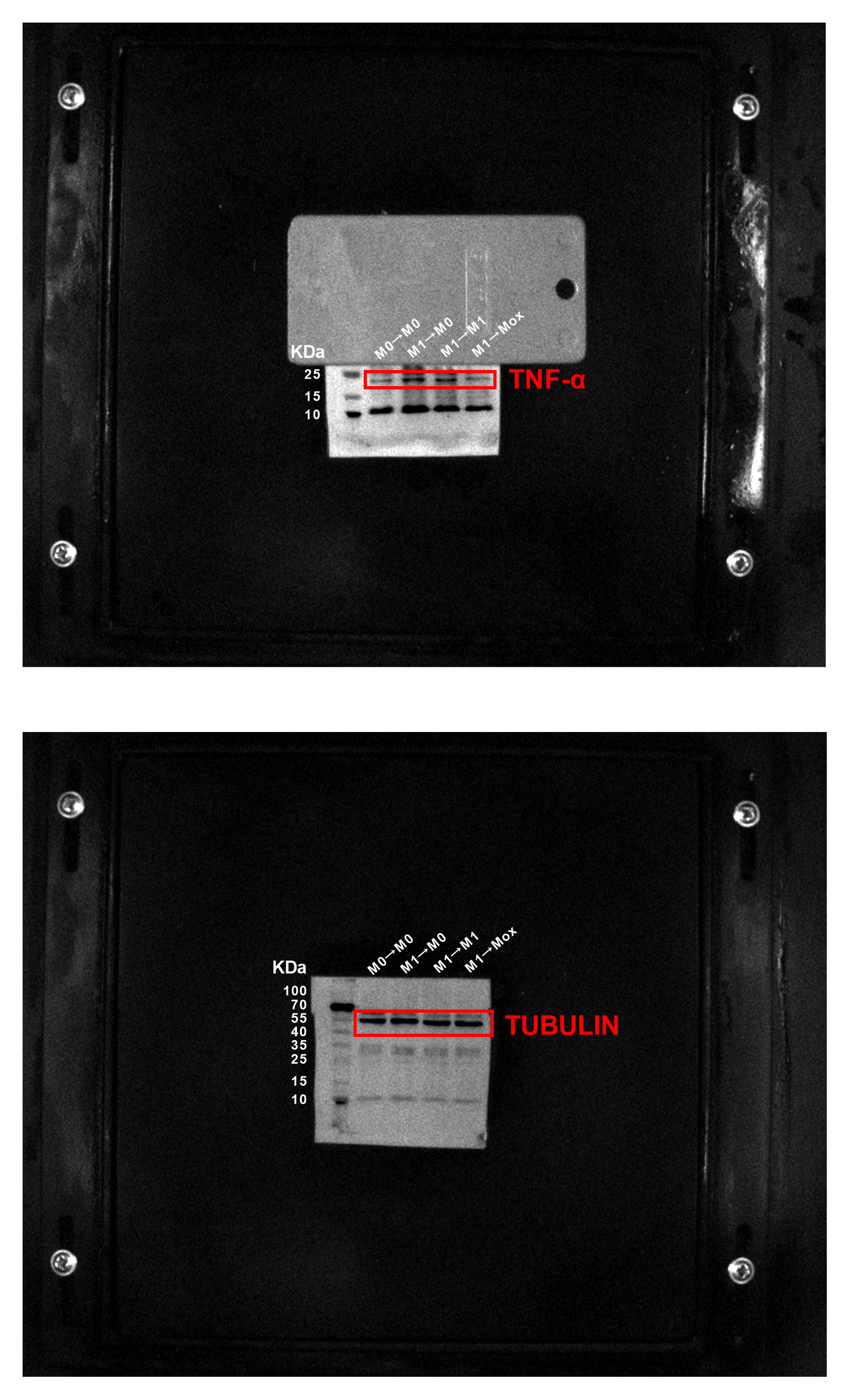

Supplement: Supplementary file 7 — Source data Fig. 5 [file 44319_2024_271_MOESM7_ESM.zip › Figure 5/Fig. 5G TNF-¦Á&TUBULIN.tif]

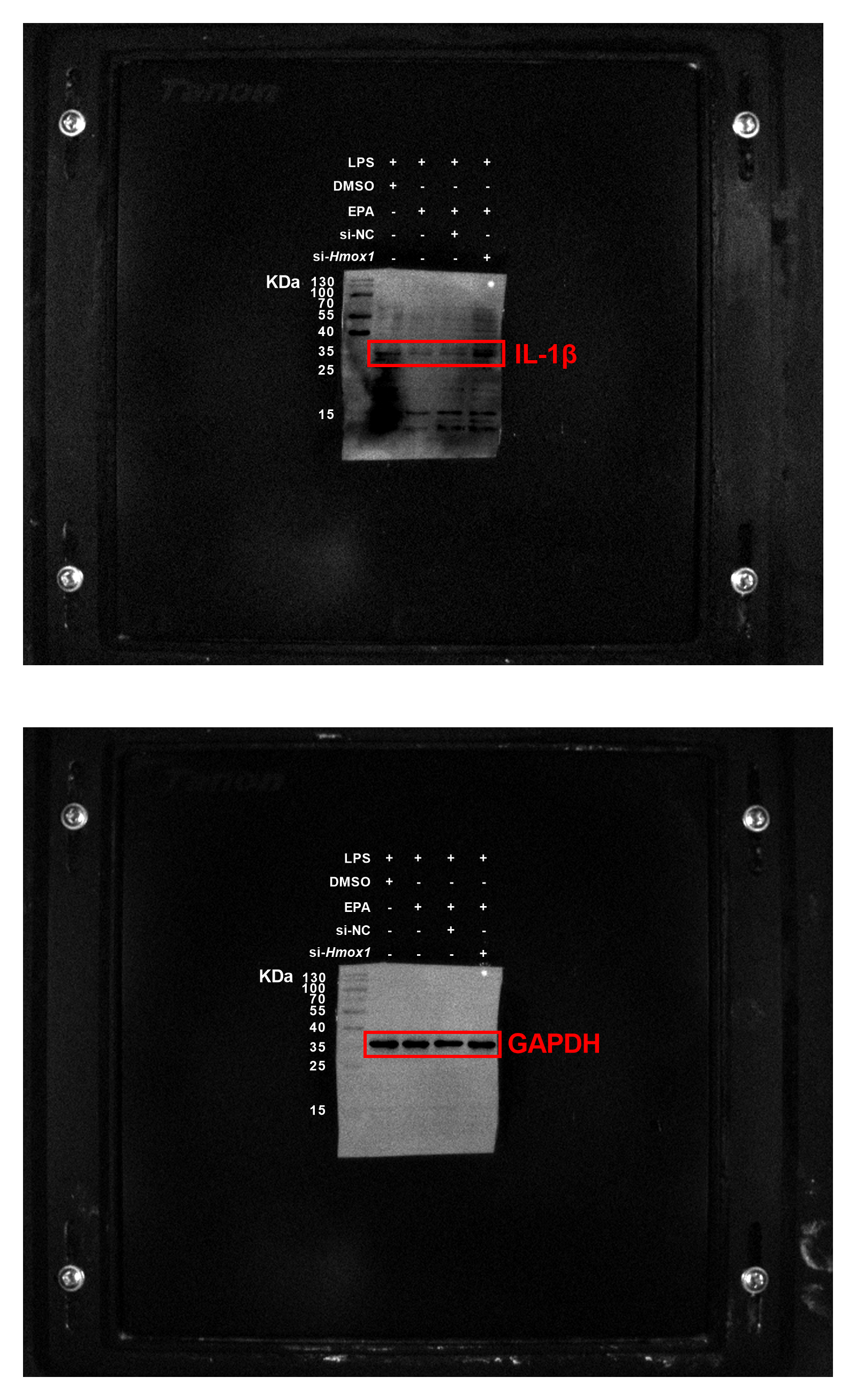

Supplement: Supplementary file 8 — Source data Fig. 6 [file 44319_2024_271_MOESM8_ESM.zip › Figure 6/Fig. 6A IL-1¦Â&GAPDH.tif]

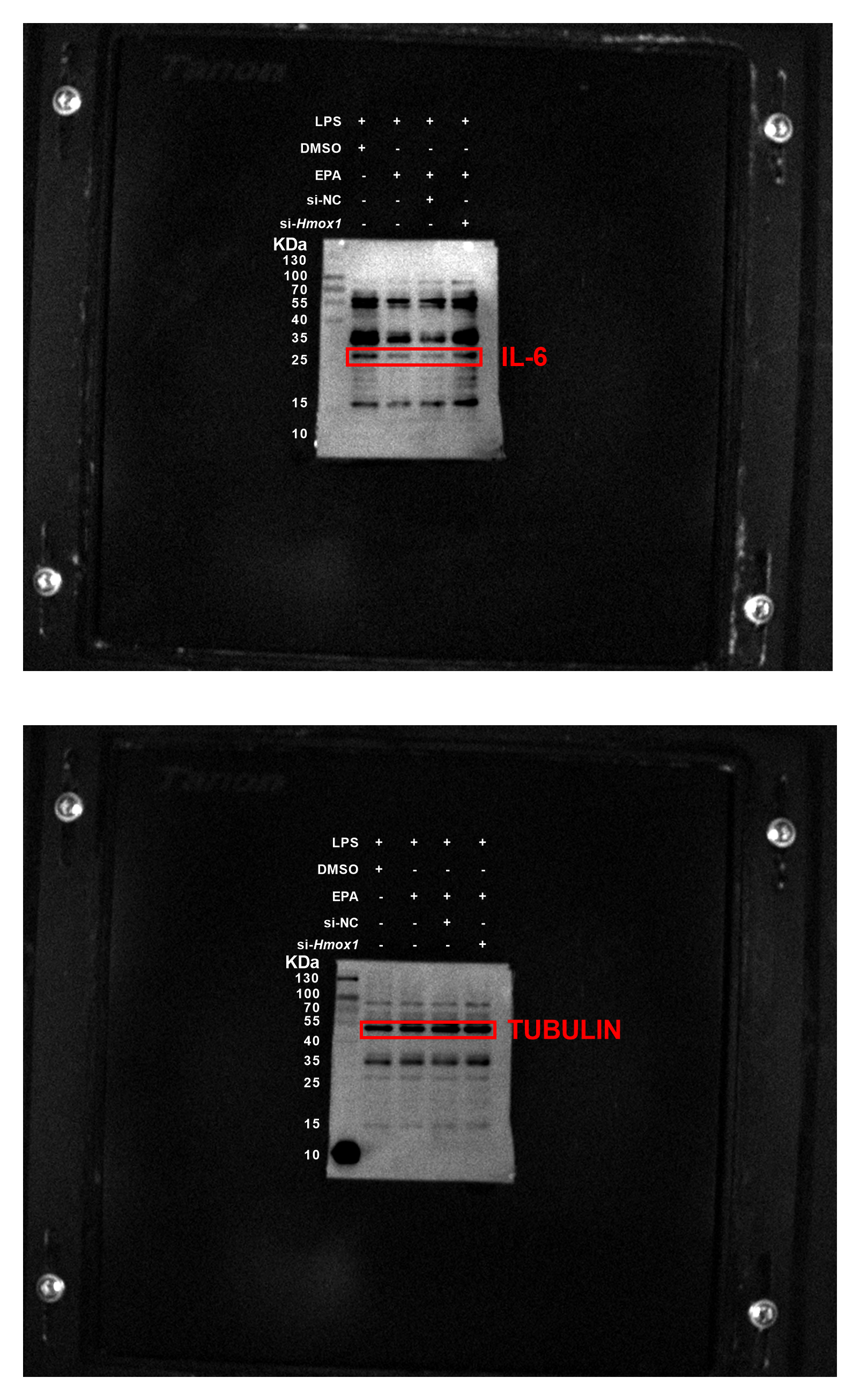

Supplement: Supplementary file 8 — Source data Fig. 6 [file 44319_2024_271_MOESM8_ESM.zip › Figure 6/Fig. 6A IL-6&TUBULIN.tif]

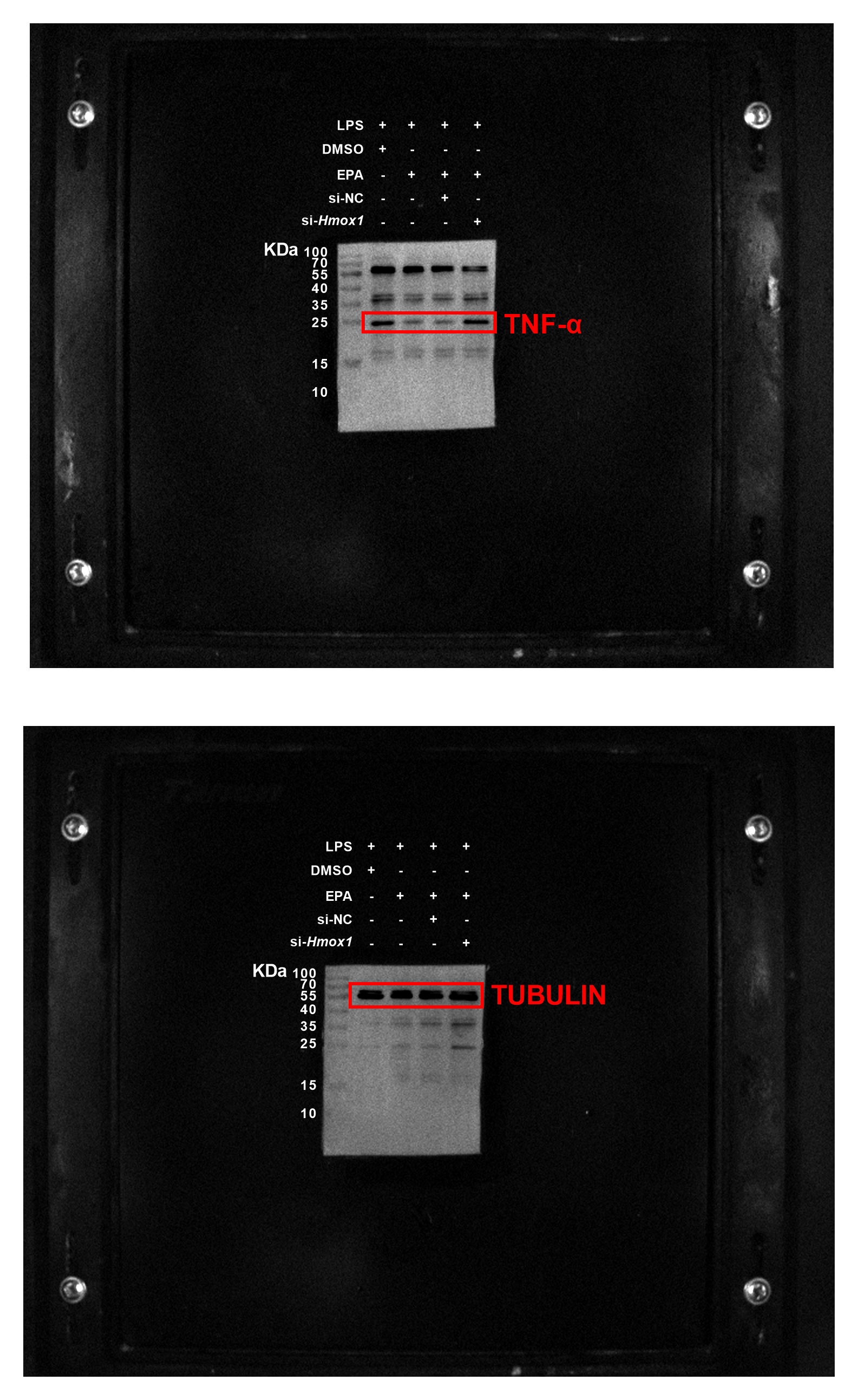

Supplement: Supplementary file 8 — Source data Fig. 6 [file 44319_2024_271_MOESM8_ESM.zip › Figure 6/Fig. 6A TNF-¦Á&TUBULIN.tif]

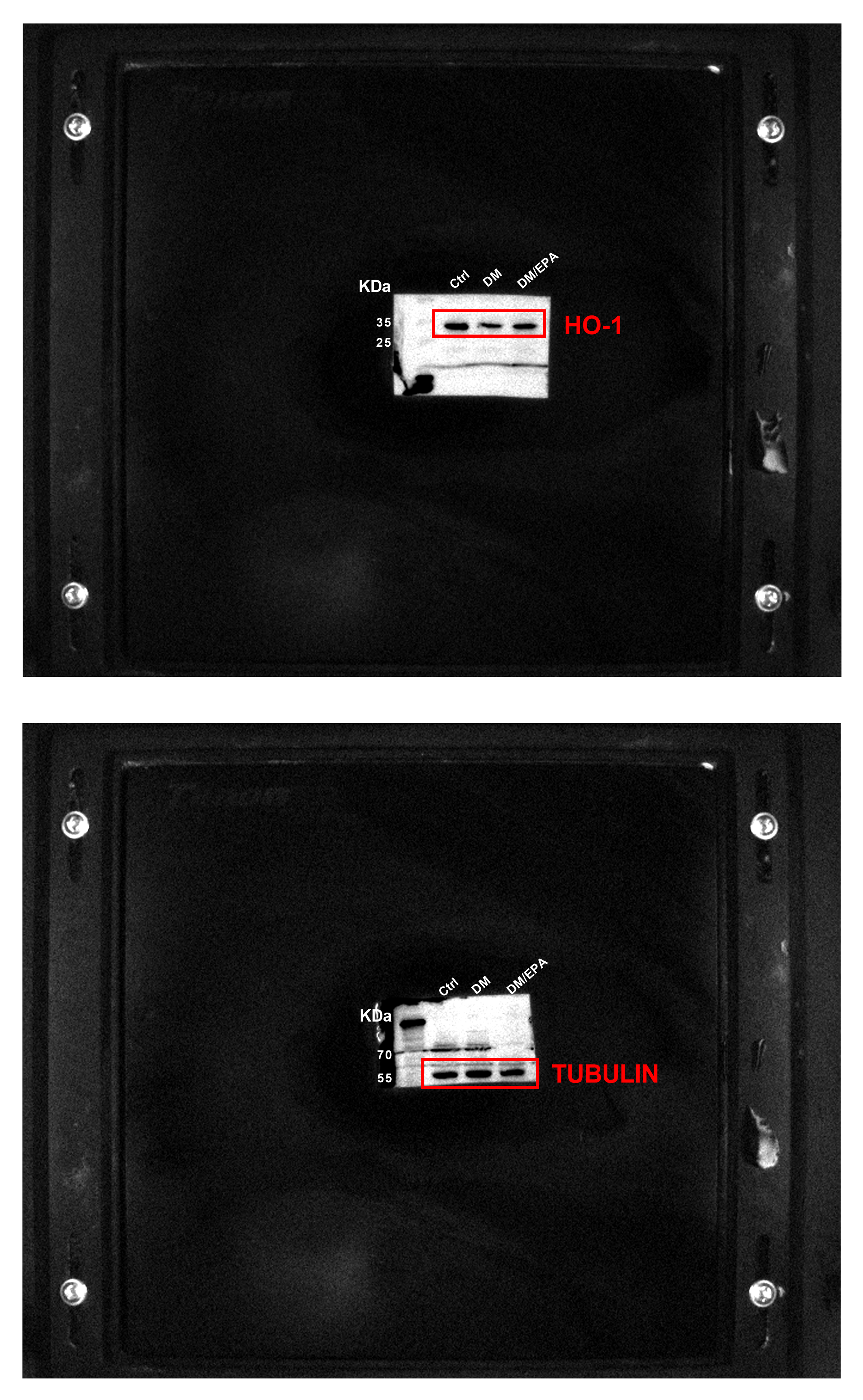

Supplement: Supplementary file 9 — Source data Fig. 7 [file 44319_2024_271_MOESM9_ESM.zip › Figure 7/Fig. 7C HO-1&TUBULIN.tif]

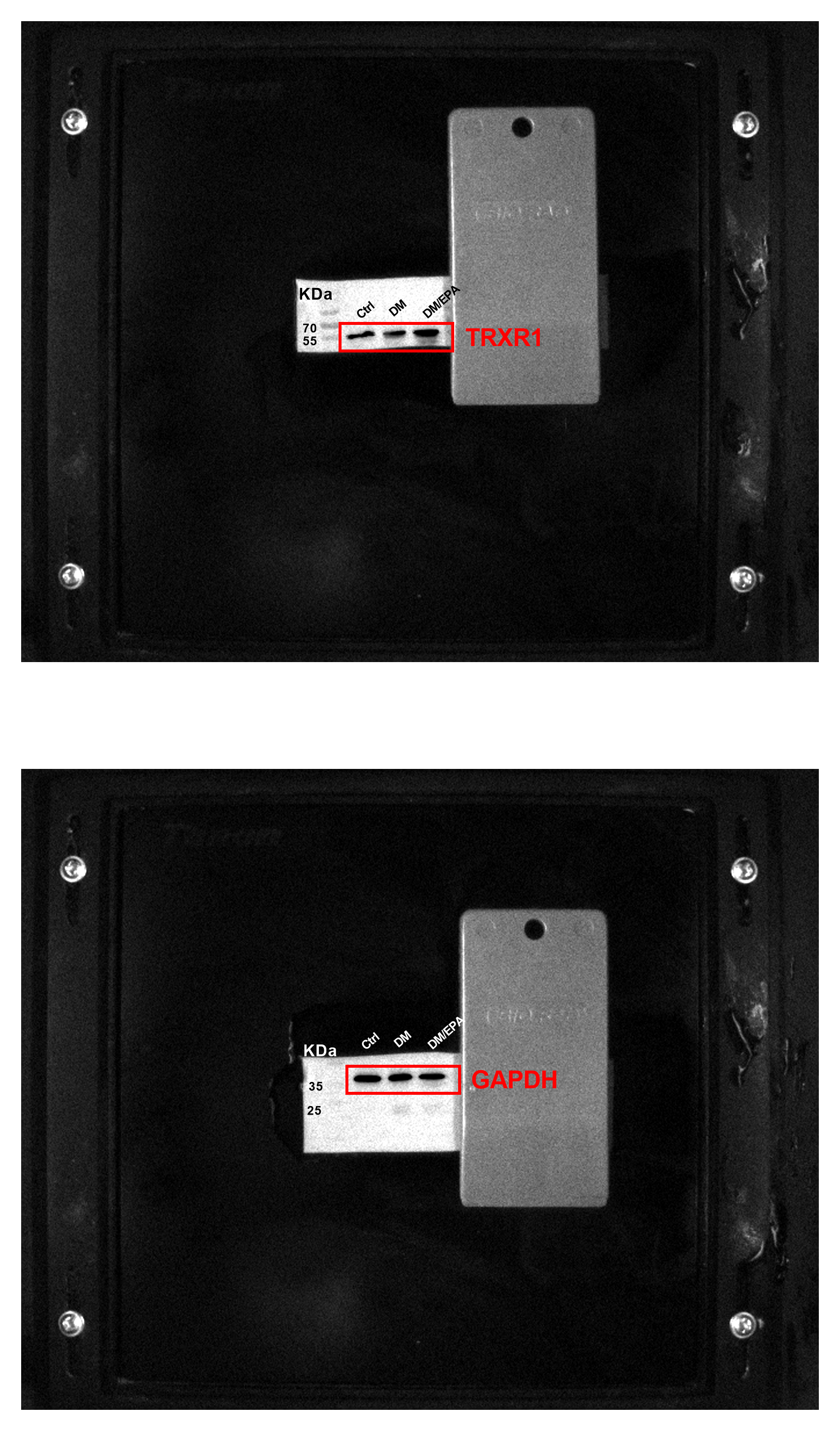

Supplement: Supplementary file 9 — Source data Fig. 7 [file 44319_2024_271_MOESM9_ESM.zip › Figure 7/Fig. 7D TRXR1&GAPDH.tif]

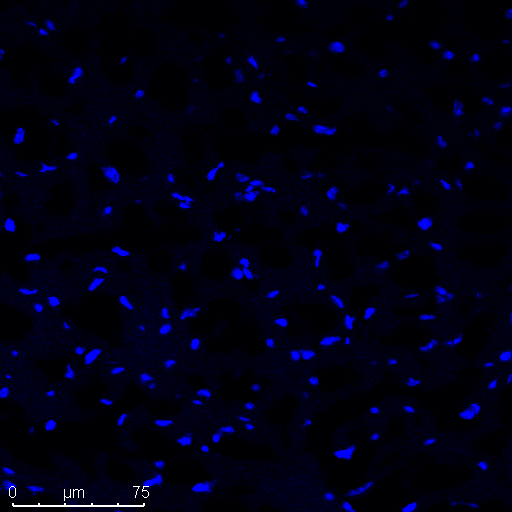

Supplement: Supplementary file 9 — Source data Fig. 7 [file 44319_2024_271_MOESM9_ESM.zip › Figure 7/Fig. 7E Ctrl-DAPI.tif]

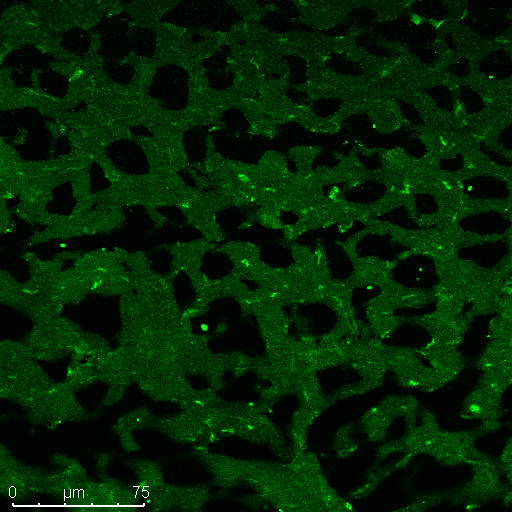

Supplement: Supplementary file 9 — Source data Fig. 7 [file 44319_2024_271_MOESM9_ESM.zip › Figure 7/Fig. 7E Ctrl-F480.tif]

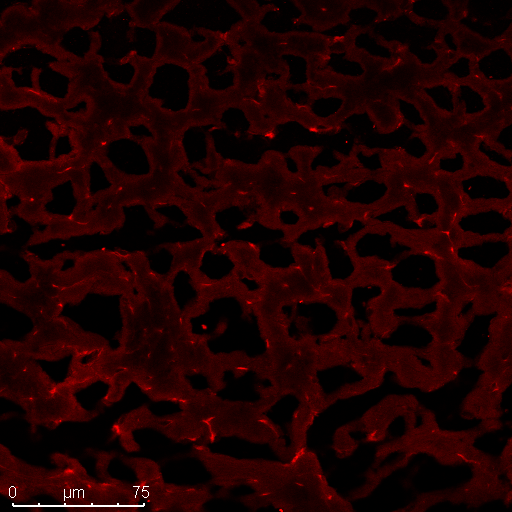

Supplement: Supplementary file 9 — Source data Fig. 7 [file 44319_2024_271_MOESM9_ESM.zip › Figure 7/Fig. 7E Ctrl-HO-1.tif]

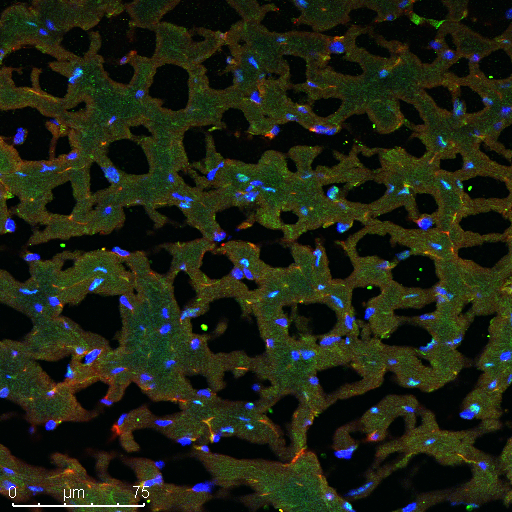

Supplement: Supplementary file 9 — Source data Fig. 7 [file 44319_2024_271_MOESM9_ESM.zip › Figure 7/Fig. 7E Ctrl-Merge.tif]

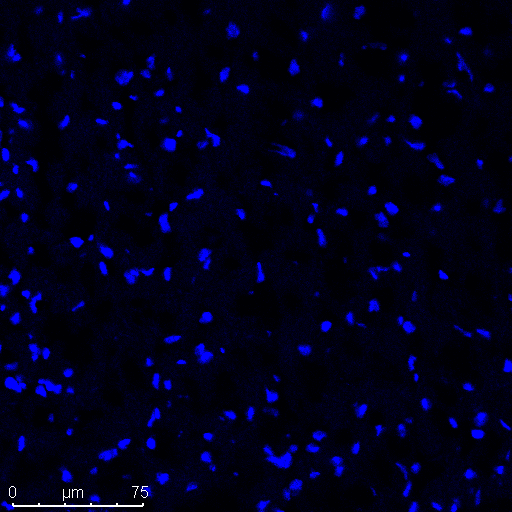

Supplement: Supplementary file 9 — Source data Fig. 7 [file 44319_2024_271_MOESM9_ESM.zip › Figure 7/Fig. 7E DM&EPA-DAPI.tif]

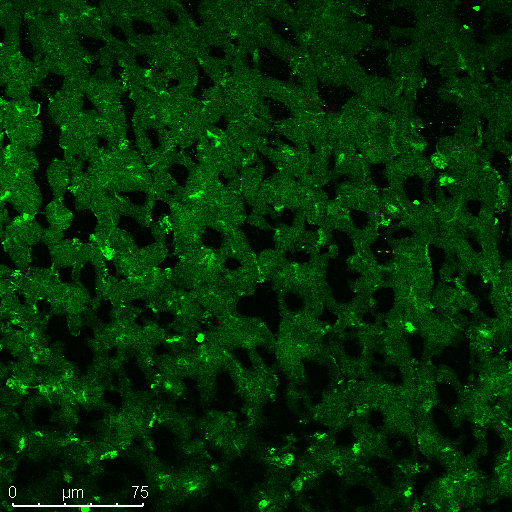

Supplement: Supplementary file 9 — Source data Fig. 7 [file 44319_2024_271_MOESM9_ESM.zip › Figure 7/Fig. 7E DM&EPA-F480.tif]

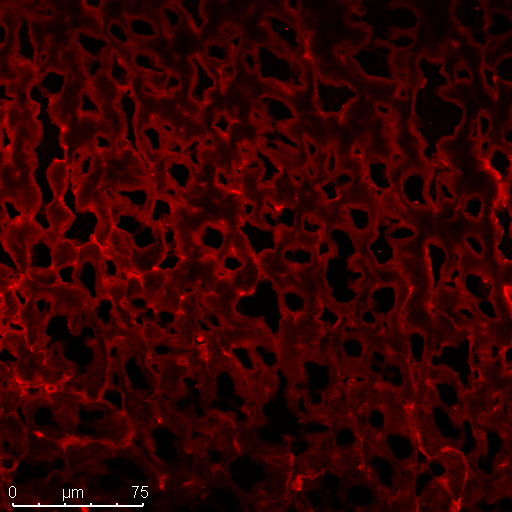

Supplement: Supplementary file 9 — Source data Fig. 7 [file 44319_2024_271_MOESM9_ESM.zip › Figure 7/Fig. 7E DM&EPA-HO-1.tif]

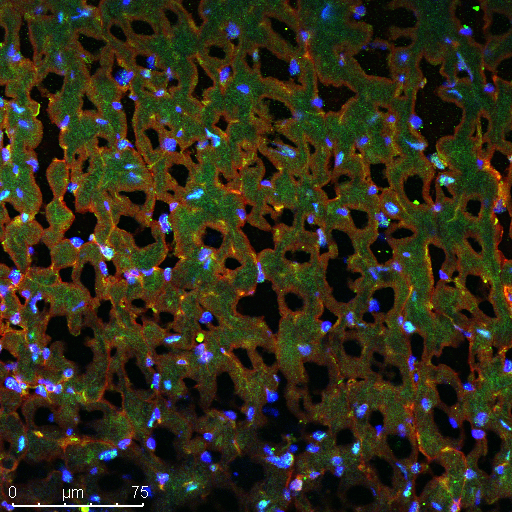

Supplement: Supplementary file 9 — Source data Fig. 7 [file 44319_2024_271_MOESM9_ESM.zip › Figure 7/Fig. 7E DM&EPA-Merge.tif]

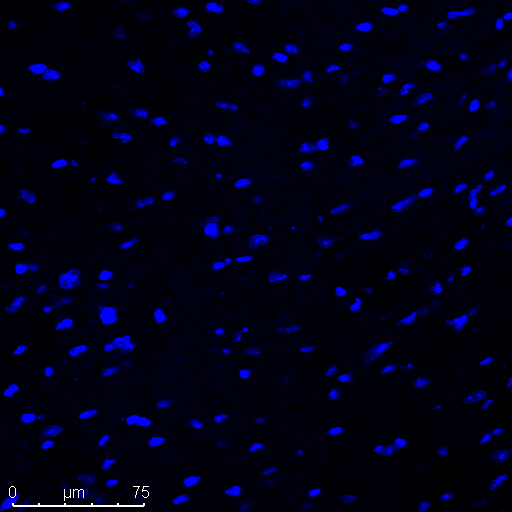

Supplement: Supplementary file 9 — Source data Fig. 7 [file 44319_2024_271_MOESM9_ESM.zip › Figure 7/Fig. 7E DM-DAPI.tif]

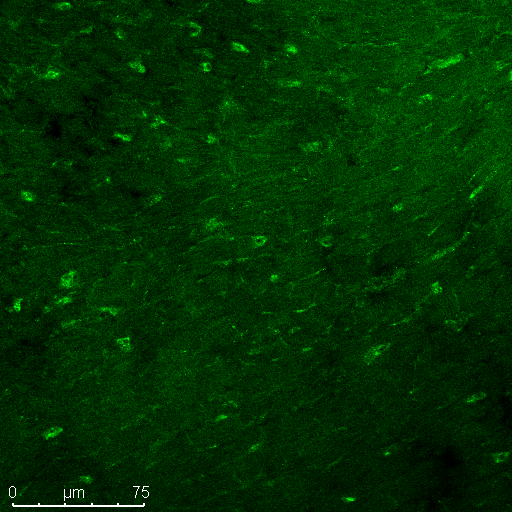

Supplement: Supplementary file 9 — Source data Fig. 7 [file 44319_2024_271_MOESM9_ESM.zip › Figure 7/Fig. 7E DM-F480.tif]

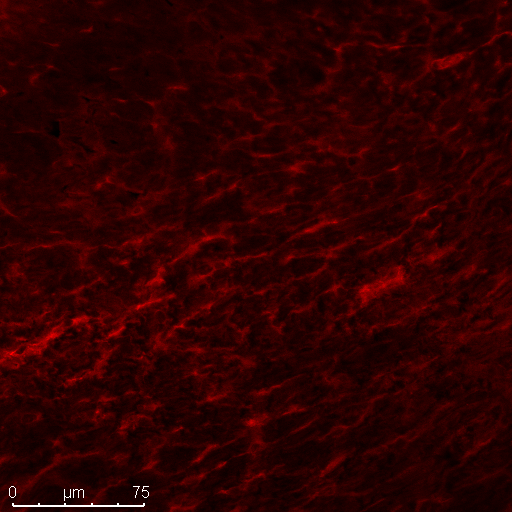

Supplement: Supplementary file 9 — Source data Fig. 7 [file 44319_2024_271_MOESM9_ESM.zip › Figure 7/Fig. 7E DM-HO-1.tif]

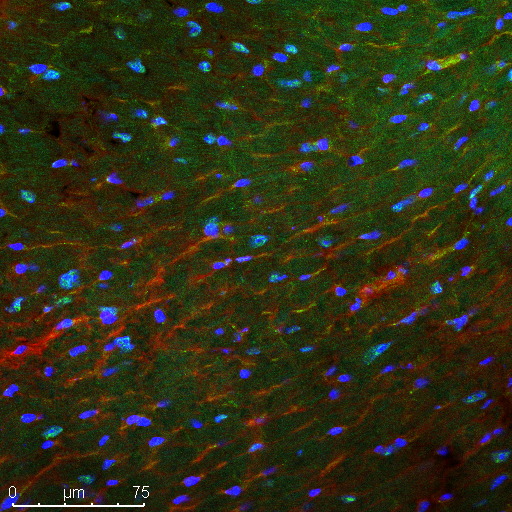

Supplement: Supplementary file 9 — Source data Fig. 7 [file 44319_2024_271_MOESM9_ESM.zip › Figure 7/Fig. 7E DM-Merge.tif]

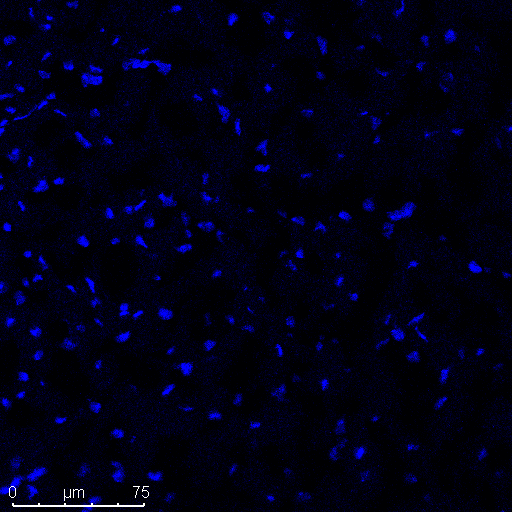

Supplement: Supplementary file 9 — Source data Fig. 7 [file 44319_2024_271_MOESM9_ESM.zip › Figure 7/Fig. 7F Ctrl-DAPI.tif]

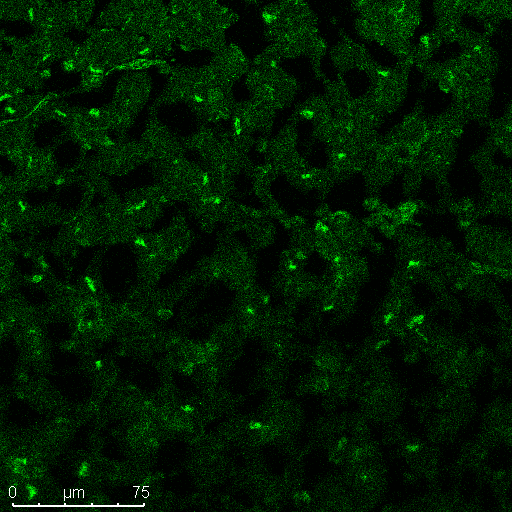

Supplement: Supplementary file 9 — Source data Fig. 7 [file 44319_2024_271_MOESM9_ESM.zip › Figure 7/Fig. 7F Ctrl-F480.tif]

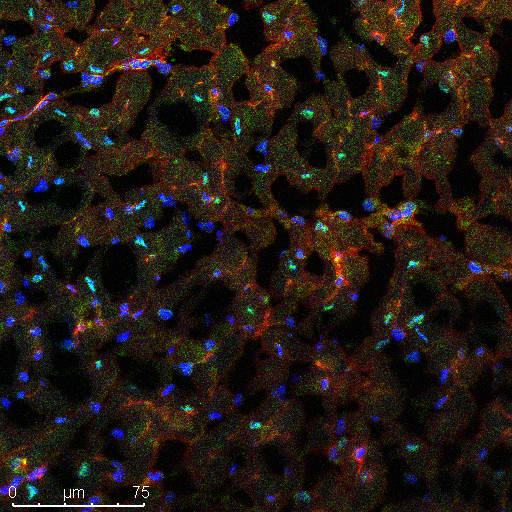

Supplement: Supplementary file 9 — Source data Fig. 7 [file 44319_2024_271_MOESM9_ESM.zip › Figure 7/Fig. 7F Ctrl-Merge.tif]

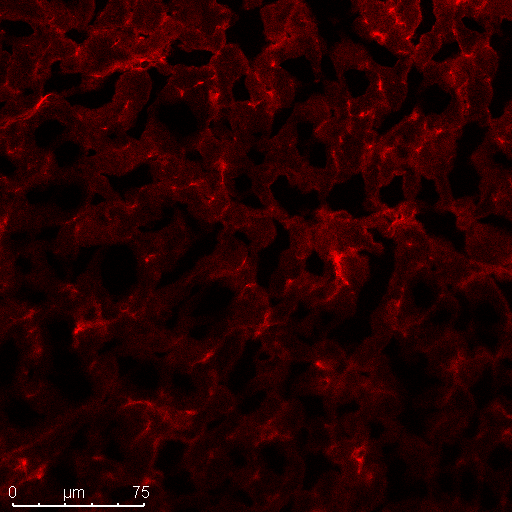

Supplement: Supplementary file 9 — Source data Fig. 7 [file 44319_2024_271_MOESM9_ESM.zip › Figure 7/Fig. 7F Ctrl-TRXR1.tif]

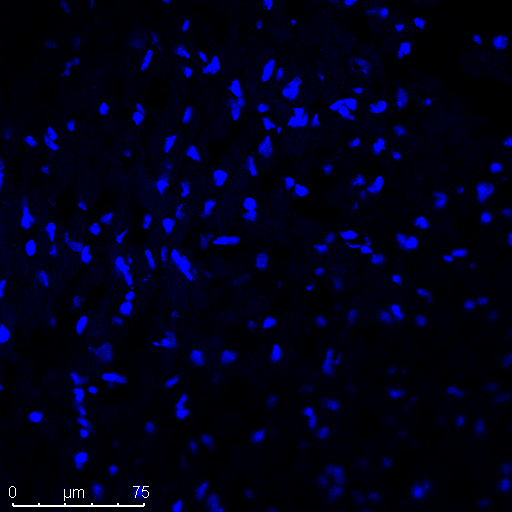

Supplement: Supplementary file 9 — Source data Fig. 7 [file 44319_2024_271_MOESM9_ESM.zip › Figure 7/Fig. 7F DM&EPA-DAPI.tif]

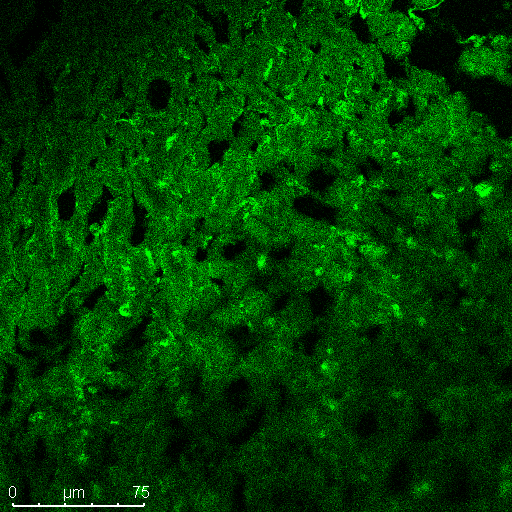

Supplement: Supplementary file 9 — Source data Fig. 7 [file 44319_2024_271_MOESM9_ESM.zip › Figure 7/Fig. 7F DM&EPA-F480.tif]

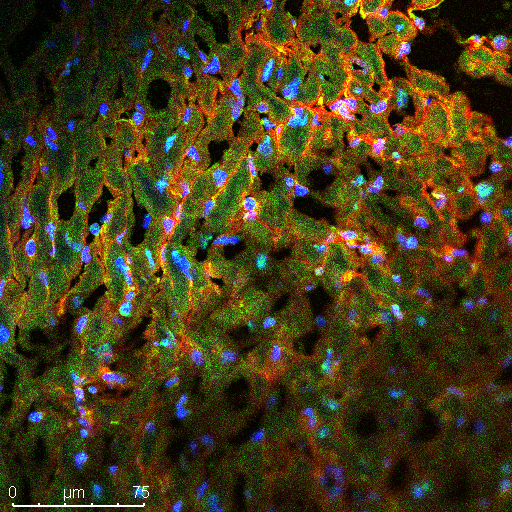

Supplement: Supplementary file 9 — Source data Fig. 7 [file 44319_2024_271_MOESM9_ESM.zip › Figure 7/Fig. 7F DM&EPA-Merge.tif]

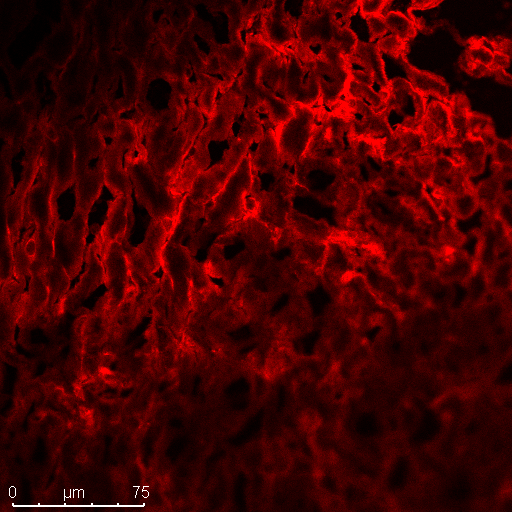

Supplement: Supplementary file 9 — Source data Fig. 7 [file 44319_2024_271_MOESM9_ESM.zip › Figure 7/Fig. 7F DM&EPA-TRXR1.tif]

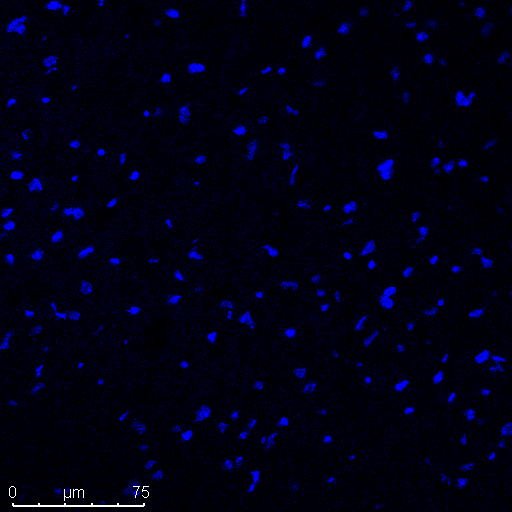

Supplement: Supplementary file 9 — Source data Fig. 7 [file 44319_2024_271_MOESM9_ESM.zip › Figure 7/Fig. 7F DM-DAPI.tif]

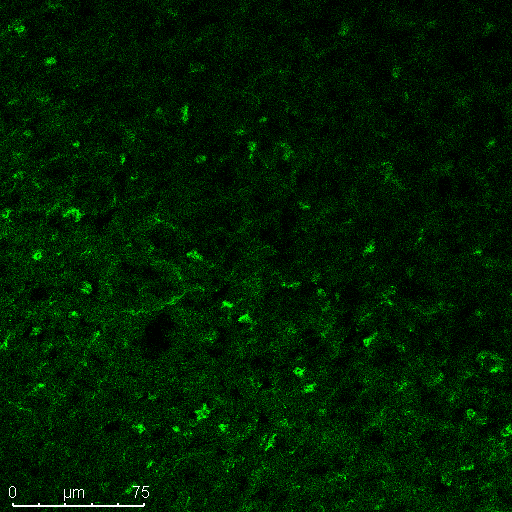

Supplement: Supplementary file 9 — Source data Fig. 7 [file 44319_2024_271_MOESM9_ESM.zip › Figure 7/Fig. 7F DM-F480.tif]

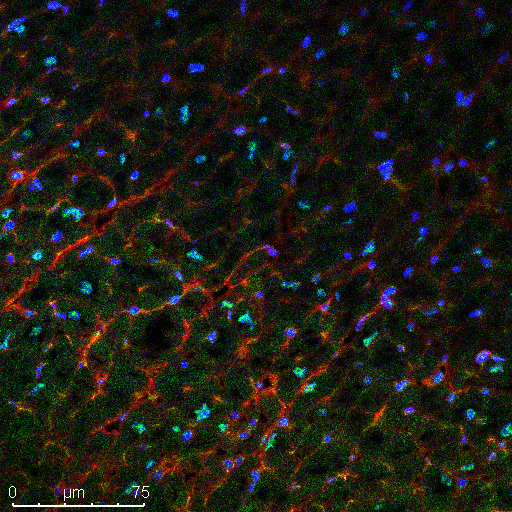

Supplement: Supplementary file 9 — Source data Fig. 7 [file 44319_2024_271_MOESM9_ESM.zip › Figure 7/Fig. 7F DM-Merge.tif]

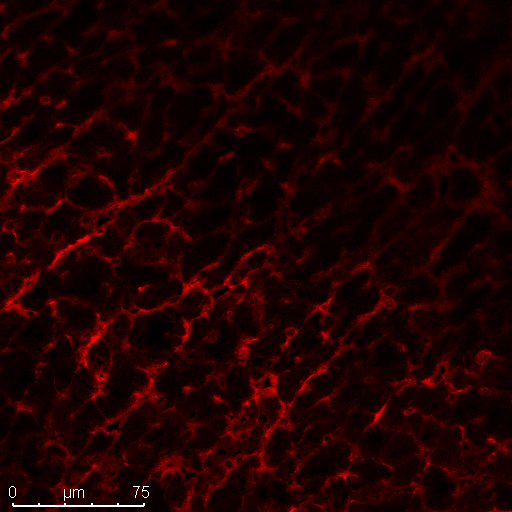

Supplement: Supplementary file 9 — Source data Fig. 7 [file 44319_2024_271_MOESM9_ESM.zip › Figure 7/Fig. 7F DM-TRXR1.tif]

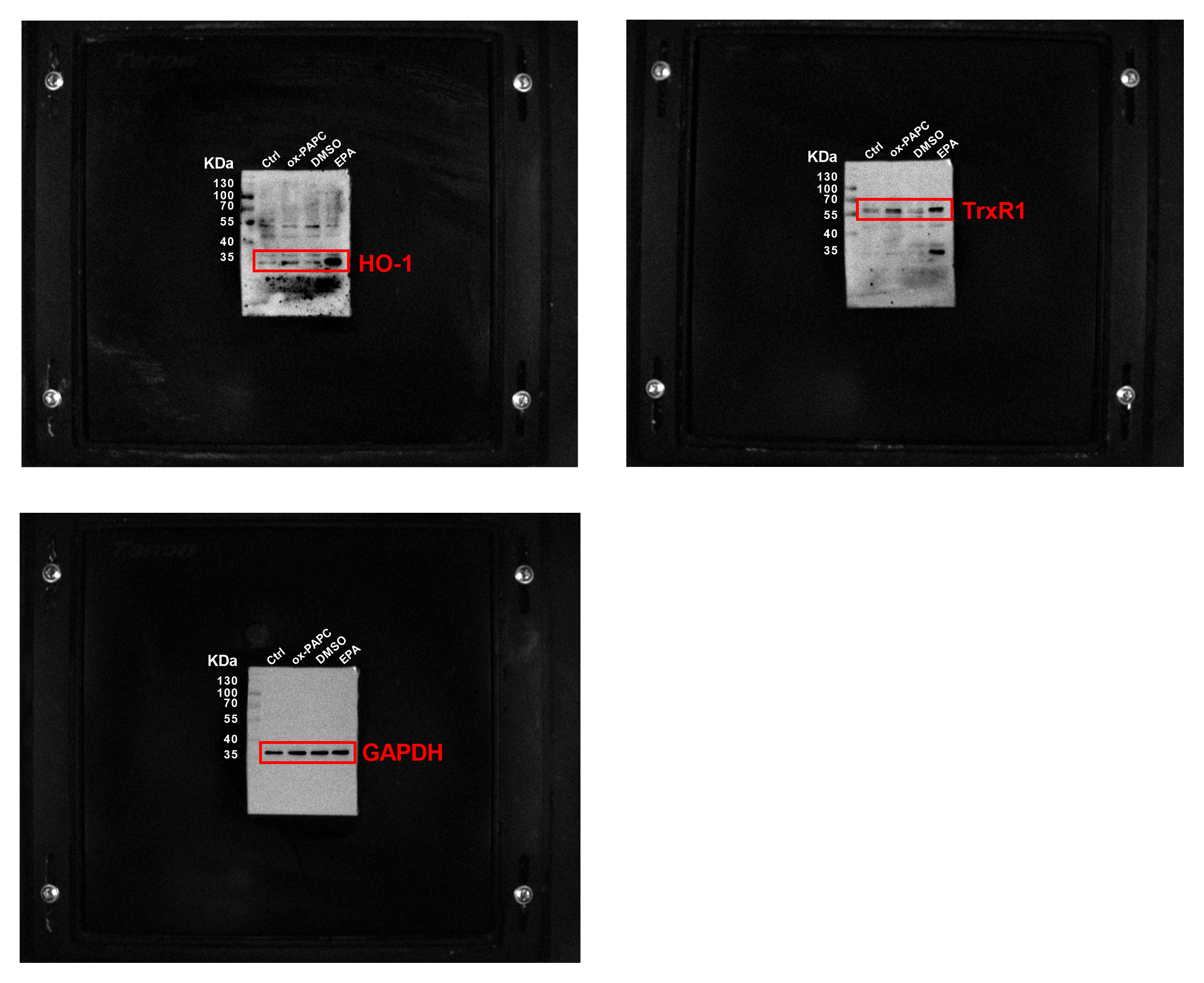

Supplement: Supplementary file 9 — Source data Fig. 7 [file 44319_2024_271_MOESM9_ESM.zip › Figure 7/Fig. 7I HO-1&TrxR1&GAPDH.tif]

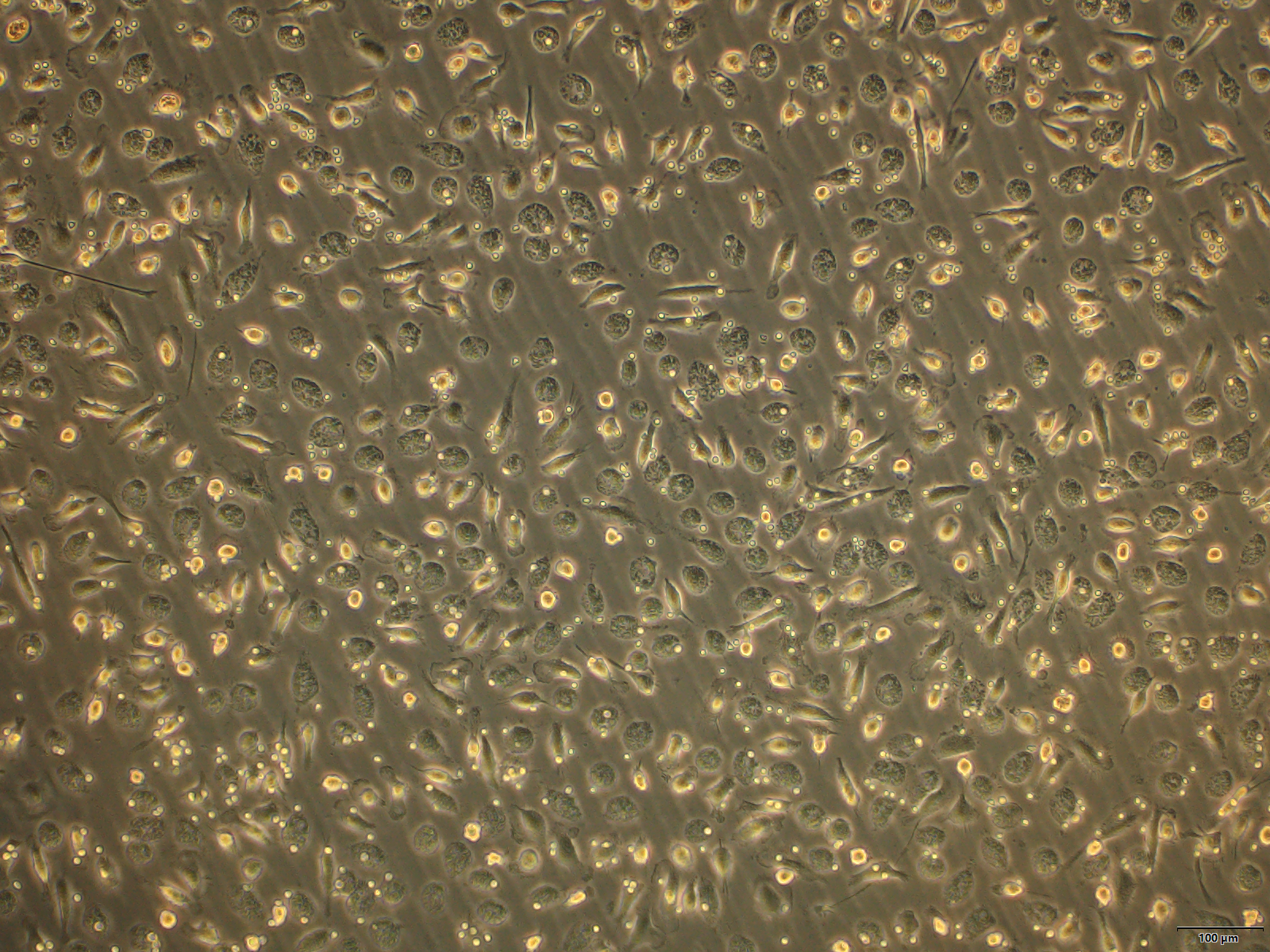

Supplement: Supplementary file 9 — Source data Fig. 7 [file 44319_2024_271_MOESM9_ESM.zip › Figure 7/Fig. 7I Macrophages.tif]

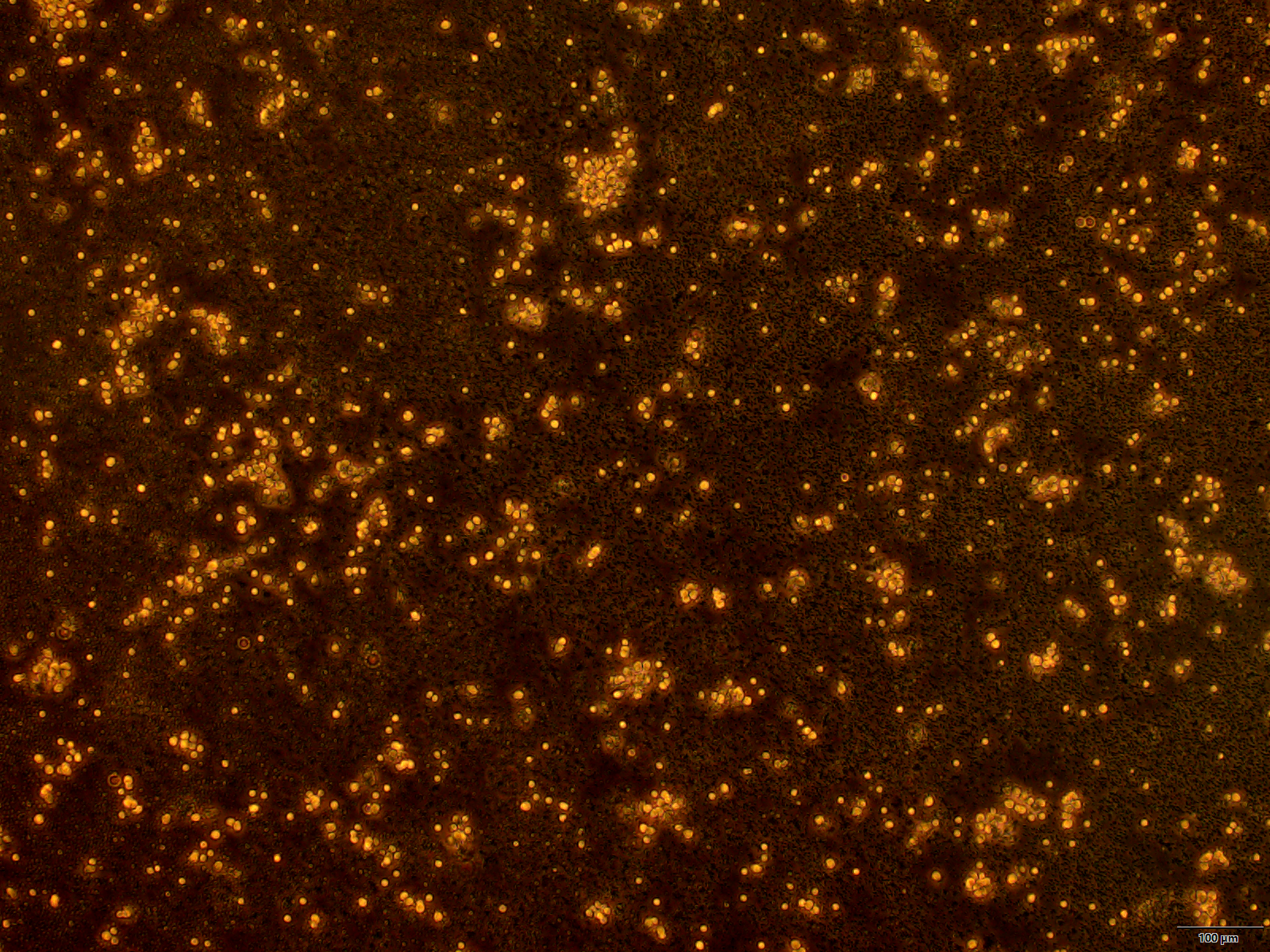

Supplement: Supplementary file 9 — Source data Fig. 7 [file 44319_2024_271_MOESM9_ESM.zip › Figure 7/Fig. 7I Monocytes.tif]
