# Supplementary figures and images for: Eicosapentaenoic acid induces macrophage Mox polarization to prevent diabetic cardiomyopathy (part 2 of 2)
Source: EMBO Rep. 2024 Oct 31;25(12):5507–36. doi: 10.1038/s44319-024-00271-x (PMC11624267; doi:10.1038/s44319-024-00271-x)

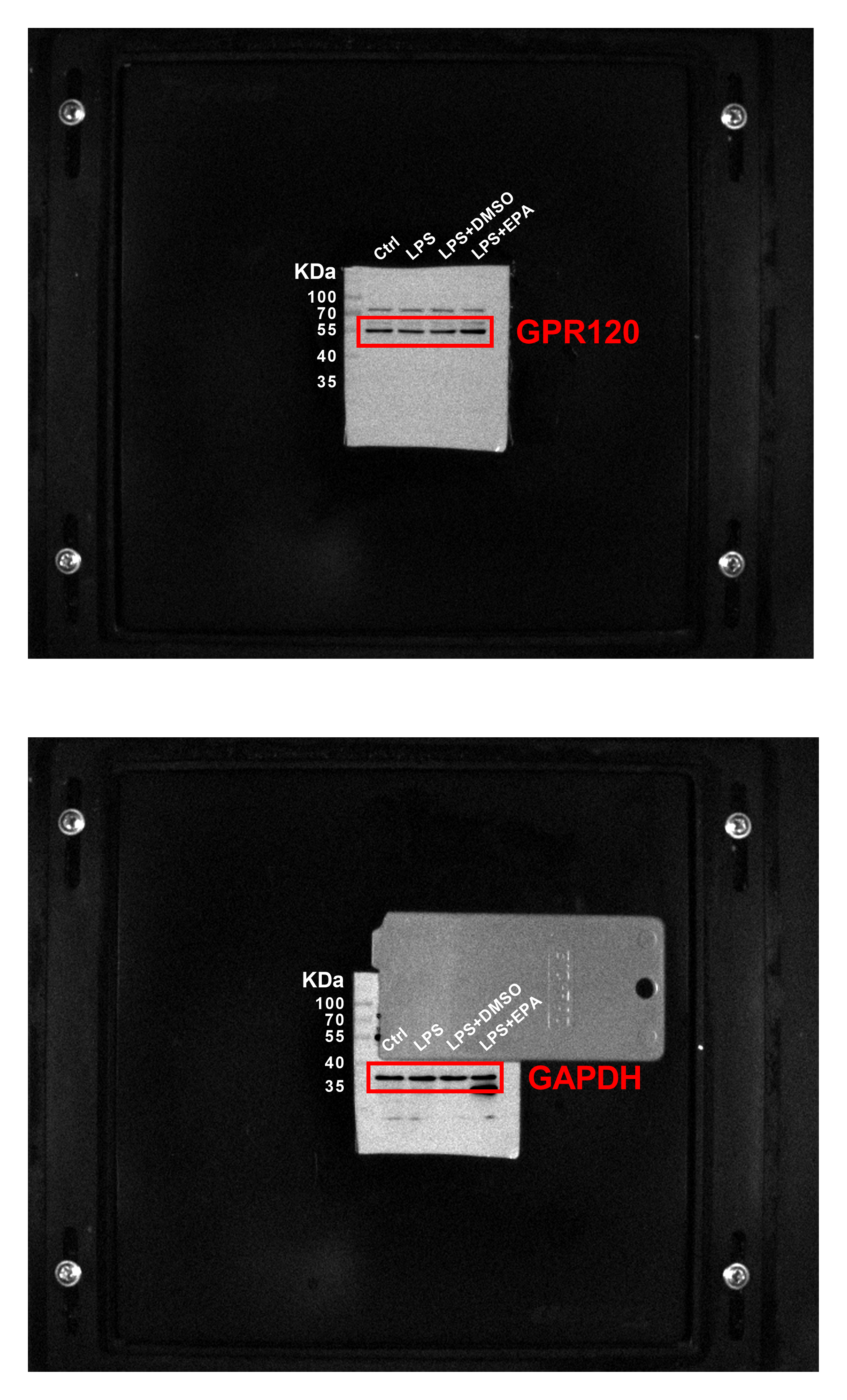

Supplement: Supplementary file 10 — Source data Fig. 8 [file 44319_2024_271_MOESM10_ESM.zip › Figure 8/Fig. 8A GPR120&GAPDH.tif]

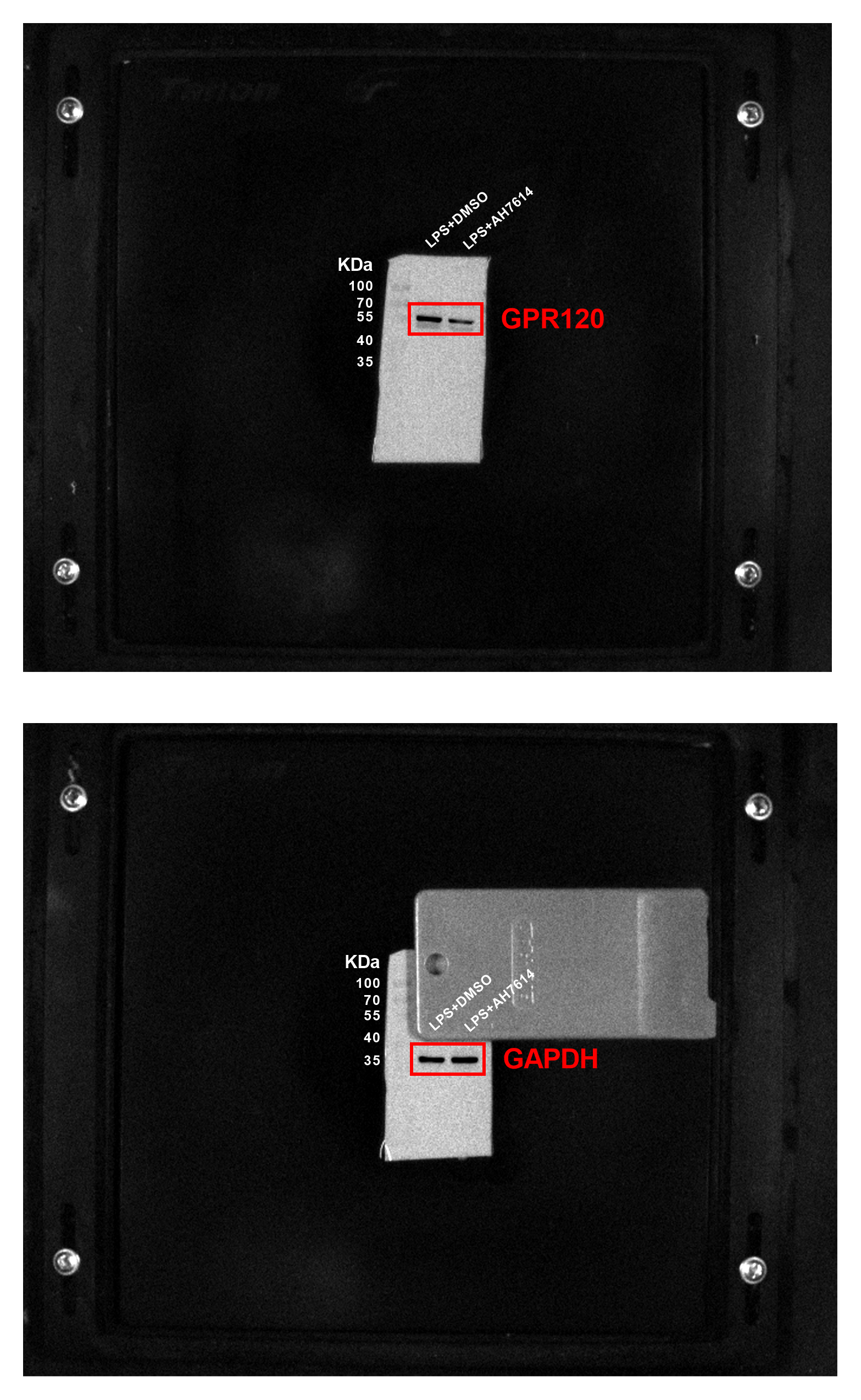

Supplement: Supplementary file 10 — Source data Fig. 8 [file 44319_2024_271_MOESM10_ESM.zip › Figure 8/Fig. 8B GPR120&GAPDH.tif]

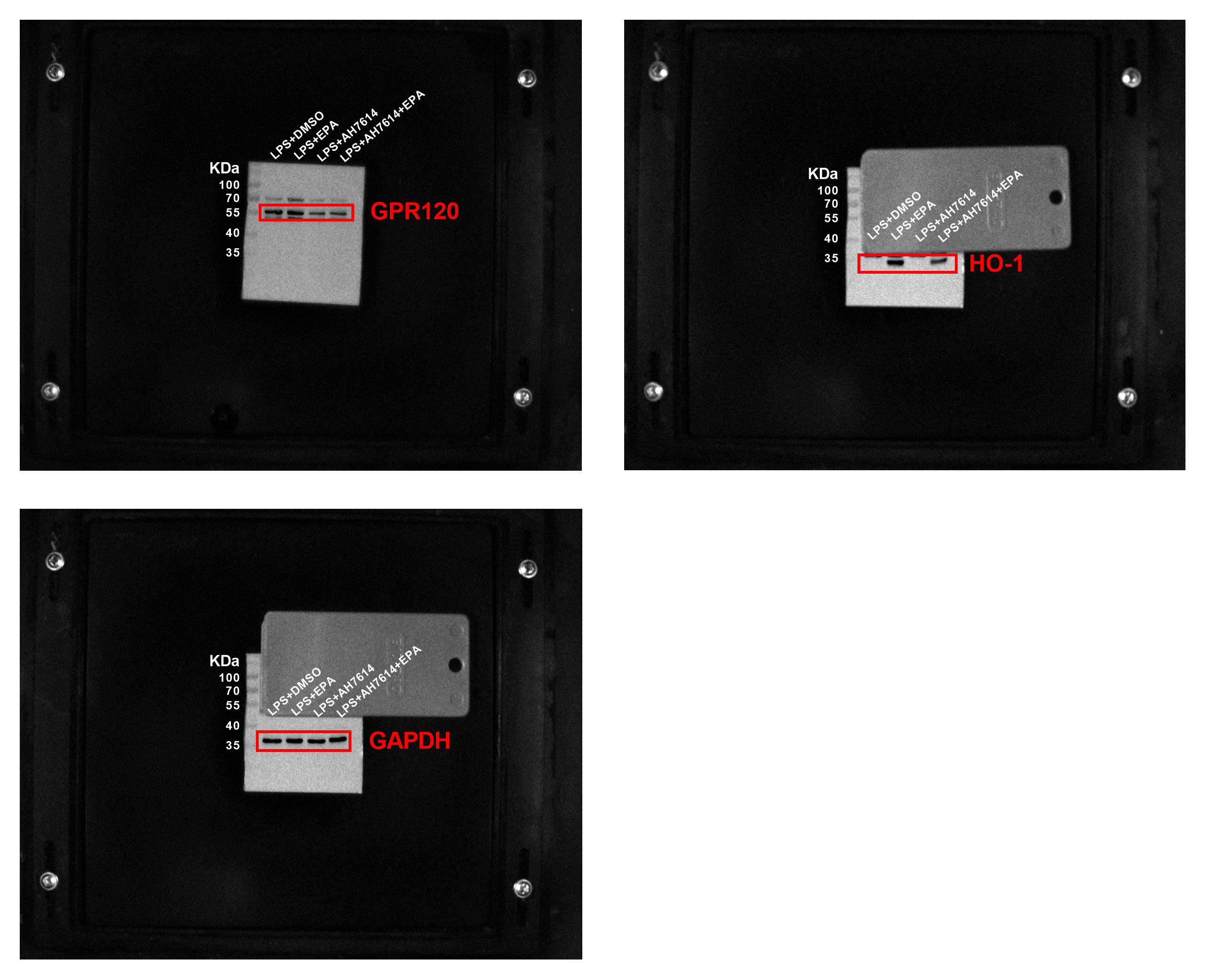

Supplement: Supplementary file 10 — Source data Fig. 8 [file 44319_2024_271_MOESM10_ESM.zip › Figure 8/Fig. 8C GPR120&HO-1&GAPDH.tif]
