# Supplementary material for: Eicosapentaenoic acid induces macrophage Mox polarization to prevent diabetic cardiomyopathy
Source: EMBO Rep. 2024 Oct 31;25(12):5507–36. doi: 10.1038/s44319-024-00271-x (PMC11624267; doi:10.1038/s44319-024-00271-x)
Supplement: Supplementary file 11 — Expanded View Figures [file 44319_2024_271_MOESM11_ESM.pdf]

Expanded View Figures

Figure EV1. EPA lowered fasting blood glucose level, and improved glucose tolerance and insulin sensitivity in the diabetic mice.

(**A**) Schematic diagram for the induction of DM and EPA treatment. (**B**) Fasting blood glucose levels. At the 20th week, ***$P < 0.001$, DM vs. Ctrl; $^{\dagger}P = 0.015$, DM/EPA vs. DM. At the 24th week, ***$P < 0.001$, DM vs. Ctrl; $^{\dagger\dagger\dagger}P < 0.001$, DM/EPA vs. DM. (**C**) Body weight of the mice. Body weight was recorded every week, and presented every 2 weeks. At the 16th week, *$P = 0.044$, DM vs. Ctrl. At the 18th week, *$P = 0.027$, DM vs. Ctrl; $^{\dagger}P = 0.011$, DM/EPA vs. DM. At the 20th week, **$P = 0.003$, DM vs. Ctrl. At the 22th week, *$P = 0.041$, DM vs. Ctrl; $^{\dagger}P = 0.032$, DM/EPA vs. DM. At the 24th week, *$P = 0.026$, DM vs. Ctrl. (**D**, **E**) Curve of blood glucose levels during GTT, and area under the curve. For (**D**), at the start, ***$P < 0.001$, DM vs. Ctrl; $^{\dagger\dagger}P = 0.002$, DM/EPA vs. DM. At the 15th minute, ***$P < 0.001$, DM vs. Ctrl. At the 30th minute, ***$P < 0.001$, DM vs. Ctrl; $^{\dagger\dagger}P = 0.009$, DM/EPA vs. DM. At the 60th minute, ***$P < 0.001$, DM vs. Ctrl; $^{\dagger\dagger}P = 0.001$, DM/EPA vs. DM. At the 90th minute, ***$P < 0.001$, DM vs. Ctrl; $^{\dagger\dagger}P = 0.001$, DM/EPA vs. DM. At the 120th minute, ***$P < 0.001$, DM vs. Ctrl; $^{\dagger\dagger\dagger}P < 0.001$, DM/EPA vs. DM. For (**E**), ***$P < 0.001$, DM vs. Ctrl; $^{\dagger\dagger\dagger}P < 0.001$, DM/EPA vs. DM. (**F**, **G**) Curve of blood glucose levels during ITT, and area under the curve. For (**F**), at the start, ***$P < 0.001$, DM vs. Ctrl; $^{\dagger\dagger\dagger}P < 0.001$, DM/EPA vs. DM. At the 15th minute, ***$P < 0.001$, DM vs. Ctrl. $^{\dagger}P = 0.01$, DM/EPA vs. DM. At the 30th, 60th, 90th, and 120th minute, ***$P < 0.001$, DM vs. Ctrl; $^{\dagger\dagger\dagger}P < 0.001$, DM/EPA vs. DM. For (**G**), ***$P < 0.001$, DM vs. Ctrl; $^{\dagger\dagger\dagger}P < 0.001$, DM/EPA vs. DM. Data information: Data are represented as individual data points of n = 10, 14, 14 (**B**), n = 10, 14, 14 (**C**), n = 10, 10, 10 (**D**, **E**), n = 7, 7, 7 (**F**, **G**) biological replicates and means ± SEM. Analysis by one-way ANOVA. AUC area under curve, EPA eicosapentaenoic acid, FBG fasting blood glucose, GTT glucose tolerance test, HFD high-fat diet, ITT insulin tolerance test, STZ streptozotocin. Groups: Ctrl control, DM diabetes mellitus, DM/EPA diabetic mice supplemented with EPA.

© The Author(s)

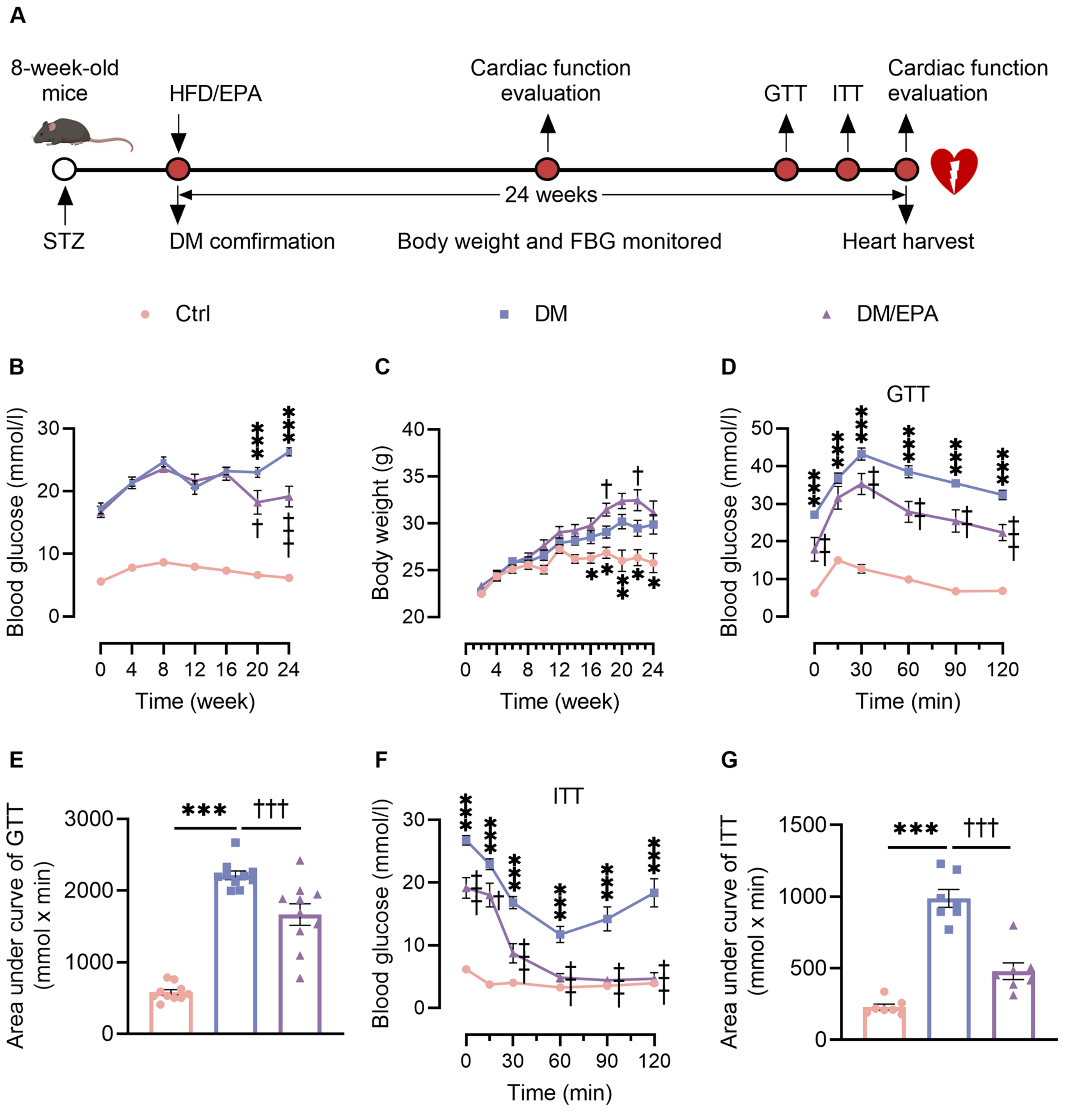
A
8-week-old mice
HFD/EPA
Cardiac function evaluation
GTT
ITT
Cardiac function evaluation
24 weeks
STZ
DM comfirmation
Body weight and FBG monitored
Heart harvest
Ctrl
DM
DM/EPA
B
Blood glucose (mmol/l)
Time (week)
C
Body weight (g)
Time (week)
D
GTT
Blood glucose (mmol/l)
Time (min)
E
Area under curve of GTT (mmol x min)
F
ITT
Blood glucose (mmol/l)
Time (min)
G
Area under curve of ITT (mmol x min)

 © The Author(s)

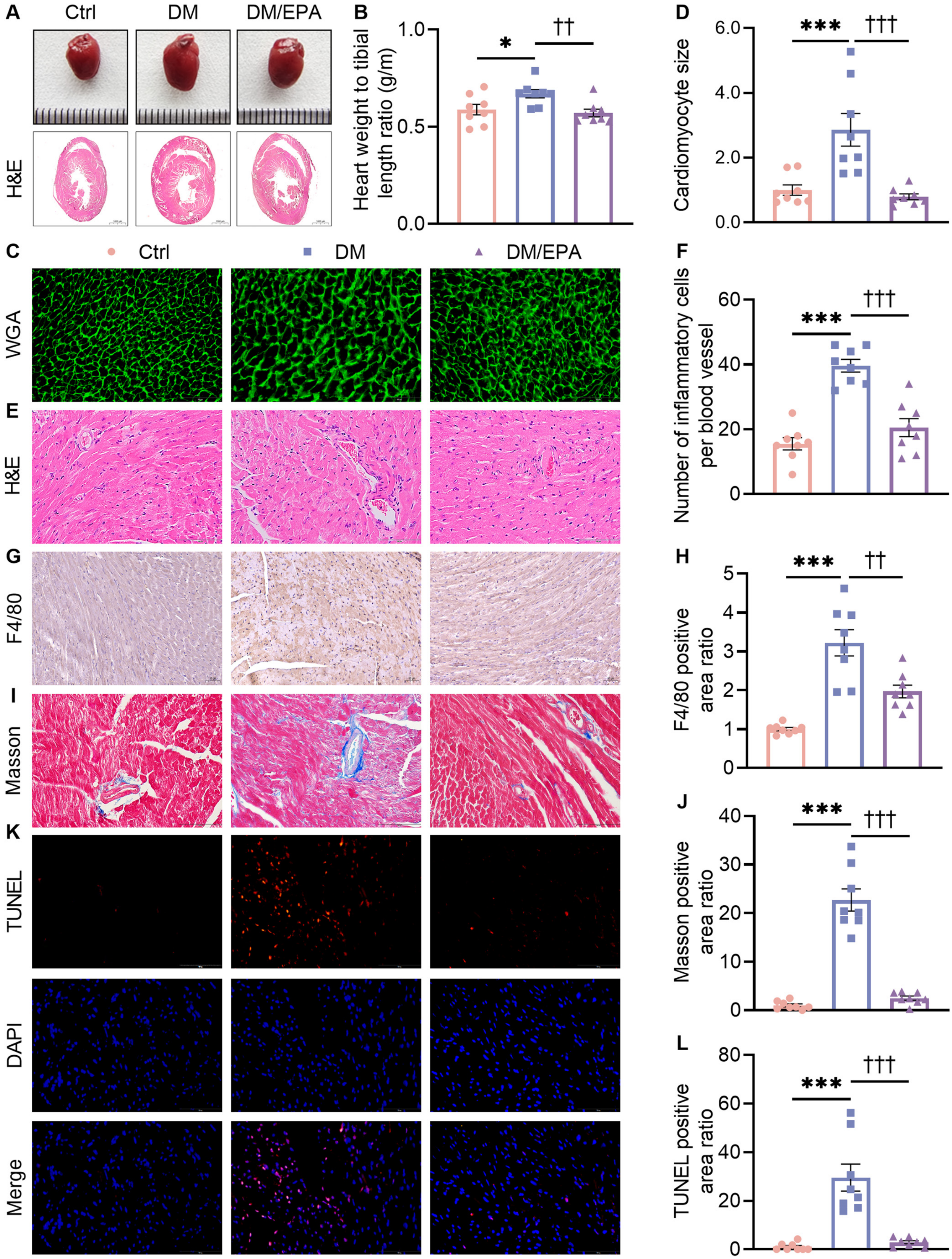
A
Ctrl
DM
DM/EPA
H&E
B
Heart weight to tibial length ratio (g/m)
1.0
0.5
0.0
*
††
C
Ctrl
DM
DM/EPA
WGA
D
Cardiomyocyte size
6.0
4.0
2.0
0.0
***
†††
E
H&E
F
Number of inflammatory cells per blood vessel
60
40
20
0
***
†††
G
F4/80
H
F4/80 positive area ratio
5
4
3
2
1
0
***
††
I
Masson
J
Masson positive area ratio
40
30
20
10
0
***
†††
K
TUNEL
DAPI
Merge
L
TUNEL positive area ratio
80
60
40
20
0
***
†††

Figure EV2. EPA prevented DM-induced cardiac pathological injuries.

(**A**) Representative gross images of the entire hearts (upper panel, bar = 1 mm), and cross-section images of H&E staining of the hearts (lower panel, bar = 1000 μm). (**B**) The ratio of heart-weight-to-tibia length. *$P = 0.021$, DM vs. Ctrl; ††$P = 0.006$, DM/EPA vs. DM. (**C**, **D**) WGA staining (bar = 100 μm) and quantification of cardiac hypertrophy. For (**D**), ***$P < 0.001$, DM vs. Ctrl; †††$P < 0.001$, DM/EPA vs. DM. (**E**, **F**) H&E staining (bar = 100 μm) and the number of inflammatory cells infiltrated per vessel. For (**F**), ***$P < 0.001$, DM vs. Ctrl; †††$P < 0.001$, DM/EPA vs. DM. (**G**, **H**) Immunohistochemical staining of F4/80 and quantification of positive area (bar = 50 μm). For (**H**), ***$P < 0.001$, DM vs. Ctrl; ††$P = 0.001$, DM/EPA vs. DM. (**I**, **J**) Masson's trichrome staining (bar = 100 μm) with the positive area quantified. For (**J**), ***$P < 0.001$, DM vs. Ctrl; †††$P < 0.001$, DM/EPA vs. DM. (**K**, **L**) TUNEL assay (bar = 100 μm) by double staining with DAPI (blue) and TUNEL (red), and ratio of the TUNEL positive nuclei. For (**L**), ***$P < 0.001$, DM vs. Ctrl; †††$P < 0.001$, DM/EPA vs. DM. Data information: For (**D**, **H**, **J**, **L**), the data are normalized to Ctrl. Data are represented as individual data points of $n = 8$ (**B**, **D**, **F**, **H**, **J**, **L**) biological replicates and means ± SEM. Analysis by one-way ANOVA. DAPI 4′,6-diamidino-2-phenylindole, TUNEL terminal deoxynucleotidyl transferase (TdT)-mediated dUTP nick-end labeling, WGA wheat germ agglutinin. Other abbreviations are the same as in Fig. EV1. Groups: Ctrl control, DM diabetes mellitus, DM/EPA diabetic mice supplemented with EPA.

© The Author(s)

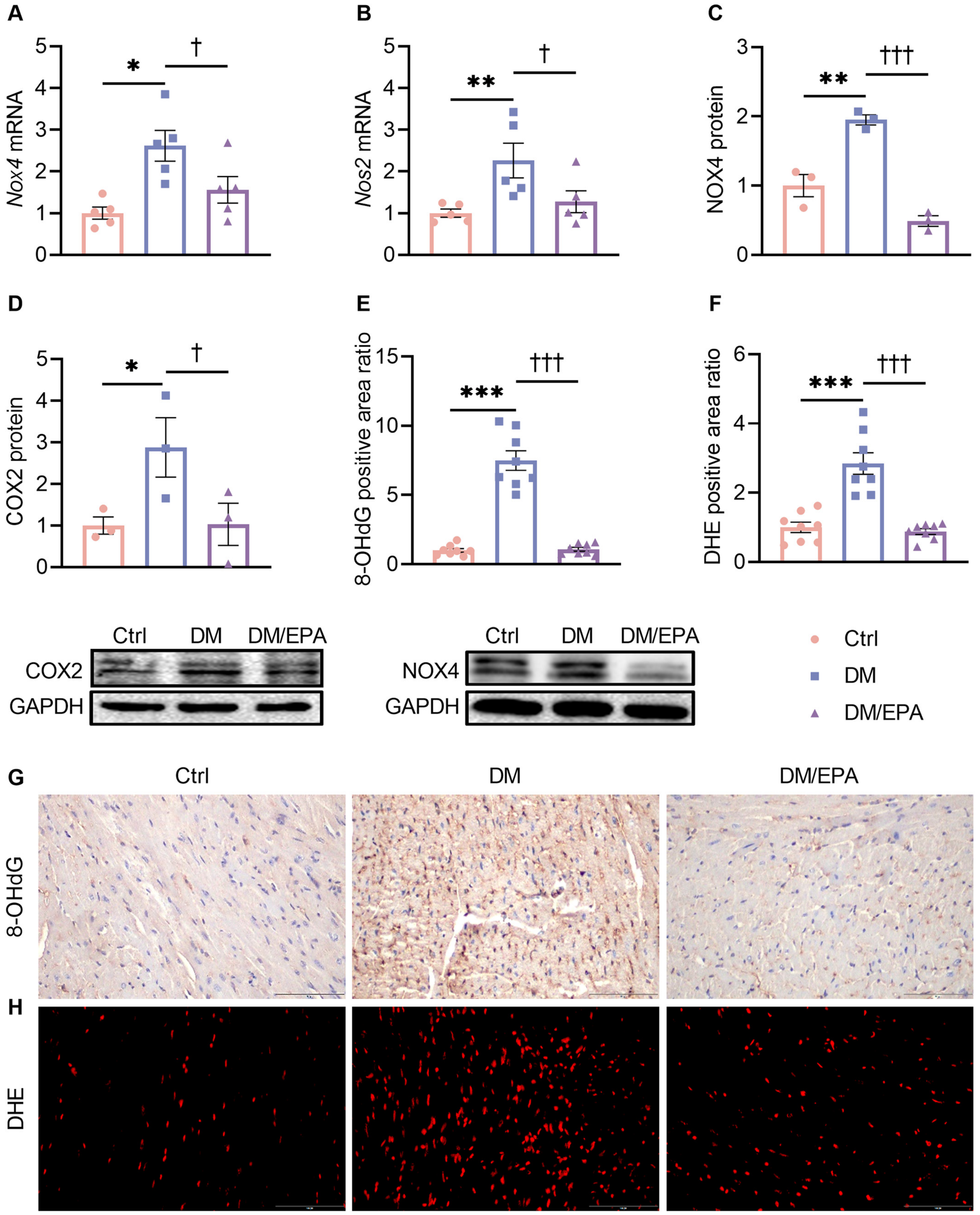
A
Nox4 mRNA
*
†
B
Nos2 mRNA
**
†
C
NOX4 protein
**
†††
D
COX2 protein
*
†
E
8-OHdG positive area ratio
***
†††
F
DHE positive area ratio
***
†††
Ctrl DM DM/EPA
COX2
GAPDH
Ctrl DM DM/EPA
NOX4
GAPDH
Ctrl
DM
DM/EPA
G
Ctrl DM DM/EPA
8-OHdG
H
DHE

Figure EV3. EPA attenuated DM-induced cardiac oxidative stress.

(**A**, **B**) mRNA expression of *Nox4* and *Nos2* were determined by qRT-PCR. For (**A**), *$P = 0.02$, DM vs. Ctrl; †$P = 0.025$, DM/EPA vs. DM. For (**B**), **$P = 0.009$, DM vs. Ctrl; †$P = 0.032$, DM/EPA vs. DM. (**C**, **D**) Protein levels of NOX4 and COX2 were determined by Western blot. For (**C**), **$P = 0.001$, DM vs. Ctrl; †††$P < 0.001$, DM/EPA vs. DM. For (**D**), *$P = 0.043$, DM vs. Ctrl; †$P = 0.045$, DM/EPA vs. DM. (**E**) Quantification of 8-OHdG positive area of the hearts. ***$P < 0.001$, DM vs. Ctrl; †††$P < 0.001$, DM/EPA vs. DM. (**F**) Quantification of DHE positive area of the hearts. ***$P < 0.001$, DM vs. Ctrl; †††$P < 0.001$, DM/EPA vs. DM. (**G**) Immunohistochemical staining of 8-OHdG (bar = 100 μm). (**H**) DHE staining of heart sections (bar = 100 μm). Data information: For (**A–F**), the data are normalized to Ctrl. Data are represented as individual data points of n = 5, 5, 5 (**A**, **B**), n = 3, 3, 3 (**C**, **D**), n = 8, 8, 8 (**E**), n = 8, 8, 8 (**F**) biological replicates and means ± SEM. Analysis by one-way ANOVA. 8-OHdG 8-hydroxy-2′-deoxyguanosine, COX2 cyclooxygenase-2, DHE dihydroethidium, *Nos2* nitric oxide synthase 2, *Nox4*/NOX4 NADPH oxidase 4. Other abbreviations are the same as in Fig. EV1. Groups: Ctrl control, DM diabetes mellitus, DM/EPA diabetic mice supplemented with EPA.

 © The Author(s)

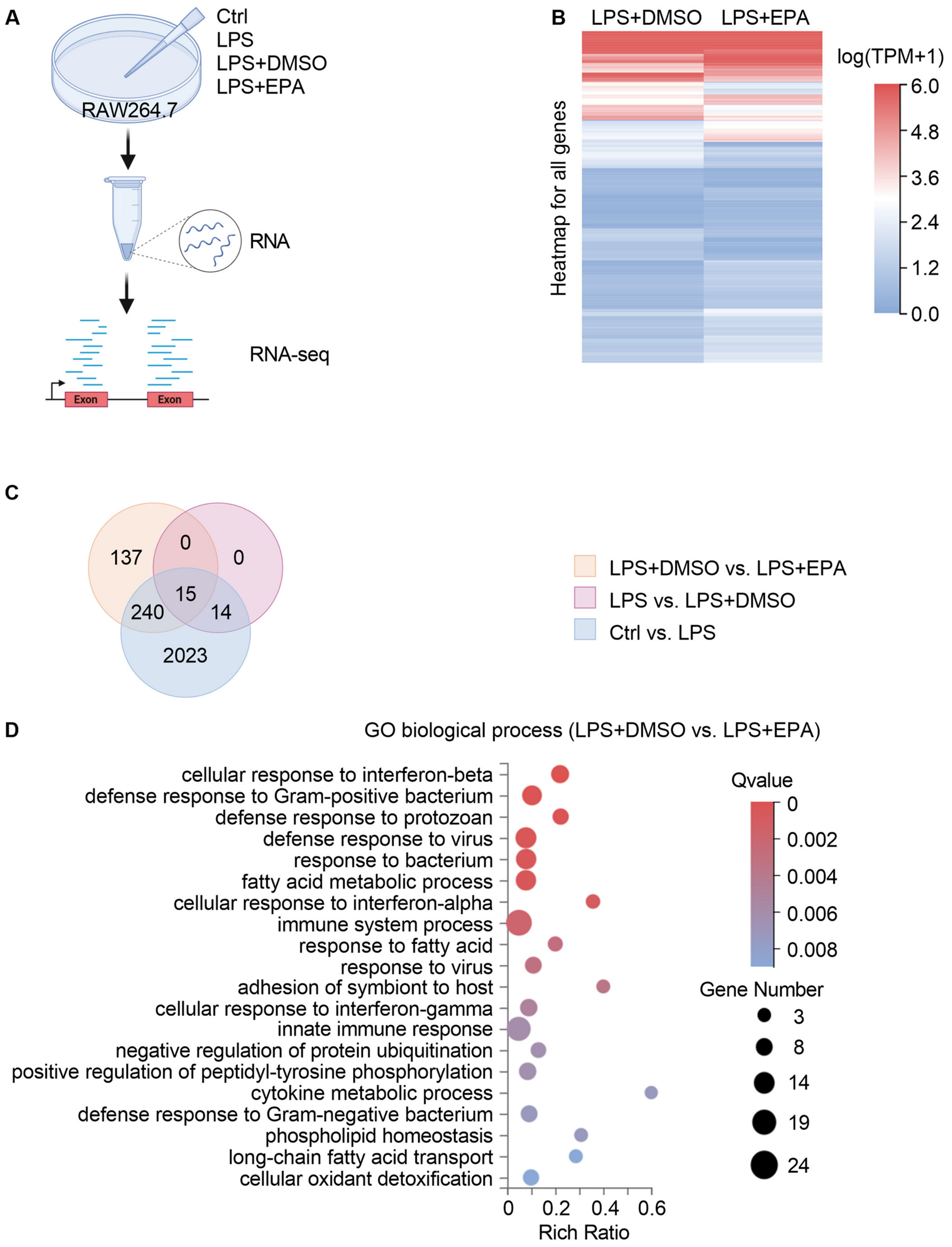
A
Ctrl
LPS
LPS+DMSO
LPS+EPA
RAW264.7
RNA
RNA-seq
Exon
Exon
B
LPS+DMSO
LPS+EPA
Heatmap for all genes
log(TPM+1)
6.0
4.8
3.6
2.4
1.2
0.0
C
137
0
0
15
240
14
2023
LPS+DMSO vs. LPS+EPA
LPS vs. LPS+DMSO
Ctrl vs. LPS
D
GO biological process (LPS+DMSO vs. LPS+EPA)
cellular response to interferon-beta
defense response to Gram-positive bacterium
defense response to protozoan
defense response to virus
response to bacterium
fatty acid metabolic process
cellular response to interferon-alpha
immune system process
response to fatty acid
response to virus
adhesion of symbiont to host
cellular response to interferon-gamma
innate immune response
negative regulation of protein ubiquitination
positive regulation of peptidyl-tyrosine phosphorylation
cytokine metabolic process
defense response to Gram-negative bacterium
phospholipid homeostasis
long-chain fatty acid transport
cellular oxidant detoxification
0
0.2
0.4
0.6
Rich Ratio
Qvalue
0
0.002
0.004
0.006
0.008
Gene Number
3
8
14
19
24

Figure EV4. EPA regulated immune-associated signal pathways in M1-polarized macrophages.

(**A**) Schematic representation of the RNA-seq protocol. (**B**) Heat map comparison of the global mRNA expression profiles in LPS + DMSO and LPS + EPA groups. (**C**) Venn diagram showing comparisons of altered gene numbers among the groups. (**D**) GO analysis for top biological processes altered by EPA compared with DMSO in LPS-challenged RAW264.7 cells. GO Gene Ontology, LPS lipopolysaccharides, RNA-seq RNA-sequencing. Other abbreviations are the same as in Fig. EV1. Groups: Ctrl control, LPS LPS-stimulated RAW264.7 cells, LPS + DMSO LPS-stimulated RAW264.7 cells treated with DMSO, LPS + EPA LPS-stimulated RAW264.7 cells treated with EPA.

 © The Author(s)

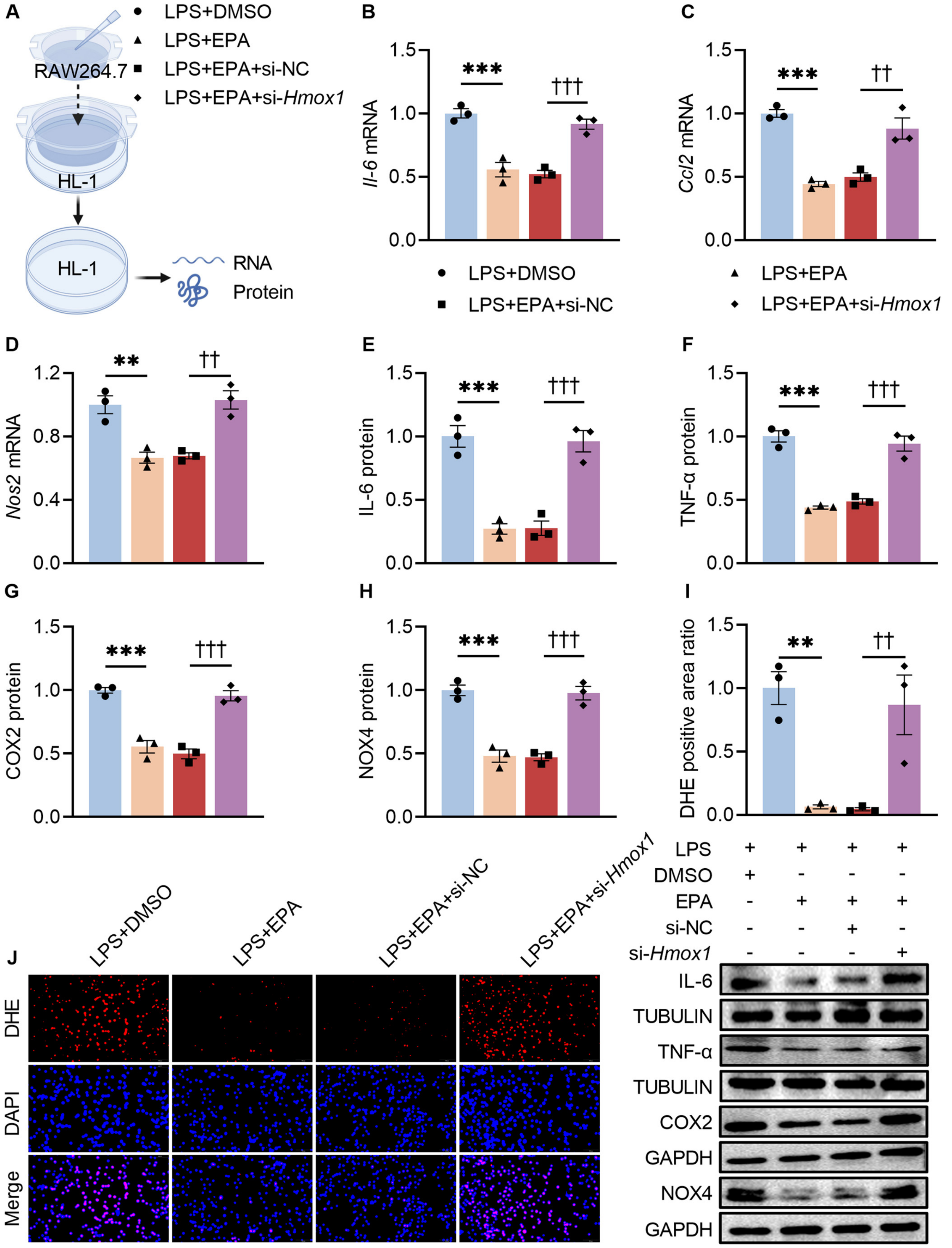
A
LPS+DMSO
LPS+EPA
LPS+EPA+si-NC
LPS+EPA+si-Hmox1
RAW264.7
HL-1
HL-1
RNA
Protein
B
Il-6 mRNA
***
†††
C
Ccl2 mRNA
***
††
LPS+DMSO
LPS+EPA
LPS+EPA+si-NC
LPS+EPA+si-Hmox1
D
Nos2 mRNA
**
††
E
IL-6 protein
***
†††
F
TNF-α protein
***
†††
G
COX2 protein
***
†††
H
NOX4 protein
***
†††
I
DHE positive area ratio
**
††
J
LPS+DMSO
LPS+EPA
LPS+EPA+si-NC
LPS+EPA+si-Hmox1
DHE
DAPI
Merge
LPS + + + +
DMSO + - - -
EPA - + + +
si-NC - - + -
si-Hmox1 - - - +
IL-6
TUBULIN
TNF-α
TUBULIN
COX2
GAPDH
NOX4
GAPDH

Figure EV5. HO-1 was required for EPA repression of cardiomyocyte injury induced by M1 polarized macrophages.

(**A**) Schematic representation of the experimental protocol. (**B–D**) mRNA levels of *Il-6*, *Ccl2*, and *Nos2* in HL-1 cells. For (**B**), ***$P < 0.001$, LPS + EPA vs. LPS + DMSO; †††$P < 0.001$, LPS + EPA+si-*Hmox1* vs. LPS + EPA+si-NC. For (**C**), ***$P < 0.001$, LPS + EPA vs. LPS + DMSO; ††$P = 0.001$, LPS + EPA+si-*Hmox1* vs. LPS + EPA+si-NC. For (**D**), **$P = 0.001$, LPS + EPA vs. LPS + DMSO; ††$P = 0.001$, LPS + EPA+si-*Hmox1* vs. LPS + EPA+si-NC. (**E–H**) Protein levels of IL-6, TNF-α, COX2, and NOX4. ***$P < 0.001$, LPS + EPA vs. LPS + DMSO; †††$P < 0.001$, LPS + EPA+si-*Hmox1* vs. LPS + EPA+si-NC. (**I**, **J**) DHE assay (bar = 100 μm) by double staining with DAPI (blue) and DHE (red), and ratio of DHE positive cells. For (**I**), **$P = 0.001$, LPS + EPA vs. LPS + DMSO; ††$P = 0.003$, LPS + EPA+si-*Hmox1* vs. LPS + EPA+si-NC. Data information: For (**B–I**), the data are normalized to LPS + DMSO. Data are represented as individual data points of $n = 3$ (**B–I**) biological replicates and means ± SEM. Analysis by one-way ANOVA. *Ccl2* C-C motif chemokine ligand 2, *Il-6*/IL-6 interleukin-6, si-*Hmox1 Hmox1* siRNA, si-NC negative control siRNA, TNF-α tumor necrosis factor-α. Other abbreviations are the same as in Figs. EV1–EV4. Groups: LPS + DMSO HL-1 cells co-cultured with LPS-stimulated RAW264.7 cells and treated with DMSO, LPS + EPA HL-1 cells co-cultured with LPS-stimulated RAW264.7 cells and treated with EPA, LPS + EPA+si-NC LPS + EPA treated with si-NC, LPS + EPA+si-*Hmox1* LPS + EPA treated with si-*Hmox1*.

© The Author(s)
